# Bioactivity-Guided Isolation of Secondary Metabolites from *Camellia fascicularis*: Antioxidative Antibacterial Activities and Anti-Inflammatory Hypoglycemic Molecular Docking

**DOI:** 10.3390/foods13213435

**Published:** 2024-10-28

**Authors:** Jiandong Tang, Jingjing Li, Boxiao Wu, Ruonan Li, Junrong Tang, Huan Kan, Ping Zhao, Yingjun Zhang, Weihua Wang, Yun Liu

**Affiliations:** 1 Key Laboratory of Forest Resources Conservation and Utilization in the Southwest Mountains of China Ministry of Education, Southwest Forestry University, Kunming 650224, China; hdyknctjd23@swfu.edu.cn (J.T.); kiki0908222@swfu.edu.cn (J.L.); hypzhao@swfu.edu.cn (P.Z.); 2 State Key Laboratory of Phytochemistry and Plant Resources in West China, Kunming Institute of Botany, Chinese Academy of Sciences, Kunming 650224, China; 3 Yunnan Key Laboratory of Gastrodia and Fungi Symbiotic Biology, Zhaotong University, Zhaotong 657000, China

**Keywords:** *Camellia fascicularis*, bioactivity-guided isolation, antioxidative, antibacterial, secondary metabolites, molecular docking

## Abstract

*Camellia fascicularis* is a valuable ornamental, edible, and medicinal plant with promising prospects for bioactivity development. We screened the bioactivity of eight fractions (Fr. A−I) obtained from the ethyl acetate phase of *C. fascicularis* via silica gel column chromatography. The results indicated that the anti-inflammatory, antioxidative, and antimicrobial active components were mainly found in Fr. B*, E, A, and H; Fr. A–G; and Fr. D–I, respectively. Bioactivity-guided isolation identified 18 secondary metabolites. Compounds **1**, **3**–**5**, **7**, and **15**–**18** were isolated from the genus *Camellia* for the first time in this study, whereas the other compounds were also isolated from this plant for the first time. The structures of these compounds were elucidated through comprehensive spectroscopic techniques. Compounds **1**, **9**−**11**, **28**, **30**, and **31** demonstrated antioxidative activities comparable to those of ascorbic acid, whereas the remaining compounds exhibited diminished antioxidative activity. In terms of antimicrobial activity, compounds **7**, **18**, **22**, and **27** exerted inhibitory potency against *Pseudomonas aeruginosa*, similar to tetracycline (MIC: 125 µg/mL). Other compounds showed moderate to weak inhibitory effects against *Staphylococcus aureus* and *Escherichia coli* (MIC: 250–500 µg/mL). Molecular docking revealed that compounds **2**, **36**, **41**, and **65** showed strong binding affinity for **8ET0**, whereas compounds **2**, **36**, **38**, **40**, **63,** and **65** showed strong binding affinity for **3A4A**. This research further increased the diversity of the secondary metabolites of *C. fascicularis*, laying a foundation for the subsequent development and utilization of this species.

## 1. Introduction

*Camellia fascicularis,* a member of the Theaceae family, is endemic to Yunnan Province in China. This rare plant, characterized by its unique golden petals and often referred to as the giant panda of the plant kingdom, queen of the tea family, and living fossil of plants, was first identified in Hekou County and is exclusively found in Gejiu, Maguan, and Hekou Counties [1]. The leaves of *C. fascicularis* are highly valued edible plant materials with high nutritional and health value due to their rich amino acid and mineral contents [2,3]. The main chemical components isolated from *C. fascicularis* are terpenoids, phenylpropanoids, indoles, phenolic acids, flavonoids and flavonoid glycosides, galactose glycerol derivatives, and lignans [4,5,6]. The leaves of *C. fascicularis*, in addition to their use as tea, exhibit a range of pharmacological properties, including antioxidative [2,3,7], antibacterial [5], antitumor [8,9], and anti-inflammatory activities [10]. While *C. fascicularis* is a valued endemic plant of Yunnan, the study of its chemical constituents started relatively late. Nevertheless, it has potentially significant biological activities.

Natural Products Chemistry Spotlight: The isolation and characterization of biologically active individual compounds from complex plant matrices must be accurately performed [11]. Activity-oriented assessment strategies performed to isolate and purify phytoconstituents are key pathways for the efficient discovery and identification of phytochemical active ingredients. Activity evaluation identifies the specific parts or fractions containing phytochemical active ingredients, enabling the isolation and purification of these active parts or fluids, which ultimately become biologically active chemical constituents of Traditional Chinese Medicine concoctions [12,13]. Forty-nine compounds have been isolated from *C. fascicularis* using bioactivity guidance, including polyphenols, flavonoids, saponins, terpenoids, lignans, galactose derivatives, and other active ingredients. Furthermore, antioxidative and antimicrobial activities are merely a small part of the problem, and the full diversity of the bioactivities of these phytochemicals is not explored [4,5,6]. The aqueous phase of *C. fascicularis* exerts antioxidative activity superior to the ethyl acetate phase [14]. Aqueous extracts of *C. fascicularis* are abundant in bioactive compounds. However, the separation process is complicated by the nature of water as a highly polar solvent, which effectively solubilizes relatively small molecules, such as polysaccharides, tannins, amino acids, proteins, organic acids, alkaloids, and glycosides. Consequently, achieving efficient separation is significantly challenging. To address the complexities affecting the accurate estimation of the abundance of bioactive compounds and characterization of solvents, future studies should explore the development of more sophisticated separation and purification methodologies to improve the isolation of these valuable constituents.

The bioactivity of *C. fascicularis* is noteworthy; however, it is limited by the low quality of the isolated secondary metabolites, which does not fully meet the criteria for bioactivity assessment. Molecular docking technology utilizes computational simulations to rapidly screen potential active compounds with high affinity for specific targets, such as enzymes and receptors. This virtual screening approach not only conserves substantial experimental time and resources but also significantly increases the efficiency of active compound discovery. The predictive outcomes of molecular docking depend on the accuracy of the model and the selection of computational methods, which are limited by current scientific advancements and computational capabilities. Consequently, predictions may sometimes not precisely reflect real-world scenarios, requiring further experimental validation [15,16,17]. Our research aims to provide a robust theoretical foundation and serve as a valuable reference for future researchers.

Current research on *C. fascicularis* focuses on cultivation, quality analysis, preliminary exploration of crude extract activity, etc. However, although *C. fascicularis* has been studied as an object of study, this paper aims to make an in-depth study of camellia from a new perspective (bioactivity-guided secondary metabolite separation) in order to discover aspects or phenomena not covered by previous studies and enrich the species of *C. fascicularis* secondary metabolites. While these data may not be entirely novel in some respects, we hope that our study will still have important implications for further understanding of the plant’s secondary metabolites, biological activity or potential applications.

Previous findings on the bioactivity of *C. fascicularis* guide current investigations. In this study, we investigated the bioactivity-directed isolation of secondary metabolites from *C. fascicularis*, extensively applied multiple separation techniques, and explored structure–activity relationships. The integration of multiple technologies enhances the research on complex secondary metabolites. The findings of this study provide a foundational basis for enhancing the diversity of secondary metabolites in *C. fascicularis* and for investigating its pharmacological effects and biological activities. Deepening our understanding of the chemical diversity inherent in *C. fascicularis* is important. The activity-guided isolation strategy we adopted successfully identified the active site of *C. fascicularis* and traced the isolation of active monomer compounds. The results of this study provide a scientific basis for the subsequent development and utilization of *C. fascicularis*. Furthermore, the identification and isolation of secondary metabolites would enable researchers to more accurately characterize their structures and functions, thereby facilitating further exploration of their potential applications across various domains, such as nutraceuticals, agriculture, and biotechnology.

## 2. Materials and Methods

### 2.1. Instrumentation

The instruments used in this study were a XEVO G2-XS Q-Tof High-Resolution Mass Spectrometer (HR-MS) (Waters, Taunton, MA, USA), Bruker AV 500 MHz Nuclear Magnetic Resonance (NMR) System (Bruker, Saarbrucken, Germany), SpectraMax 190 Microplate Reader (Molecular Devices, San Jose, CA, USA), NP7000 Semi-preparative Liquid Phase system (Jiangsu Hanbang Technology Co., Ltd., Huaian, China), AX224ZH\E Electronic Balance (Ohaus Instruments, Changzhou Co., Ltd., Changzhou, China), N-1300 rotary evaporator, (Shanghai Ailang Instrument Co., Ltd., Shanghai, China), SpectraMax 190 enzyme labeler (Molecular Devices Co., Ltd., Shanghai, China), ZQZY-CF9.9 oscillating incubator (Shanghai Zhichu Instrument Co., Ltd., Shanghai, China), and a ZF-7 triple-use UV analyzer (Shanghai Jiapeng Science and Technology Co., Ltd., Shanghai, China).

### 2.2. Chemicals and Reagents

Diclofenac sodium was provided by Shanghai Yuanye Biotechnology Co., Ltd. (Shanghai, China), 2,2′-azino-bis (3-ethylbenzothiazoline-6-sulfonic acid) (ABTS) and ascorbic acid was obtained from Beijing Solarbio Science & Technology Co., Ltd. (Beijing, China). Methanol (HPLC grade) was purchased from Shanghai Xingke High Purity Solvent Co., Ltd. (Shanghai, China). Dimethyl sulfoxide (DMSO) and all other chemicals of analytical grade were purchased from Sinopharm Chemical Reagent Co., Ltd. (Shanghai, China). Macroporous resin D101 and Sephadex LH–20 were purchased from Shanghai Yuanye Biotechnology Co., Ltd. (Shanghai, China). Column chromatography silica gel (200–300 and 300–400 mesh) and thin-layer chromatography silica gel plates were purchased from Qingdao Ocean Chemical Co., Ltd. (Qingdao, China), and middle chromatogram isolated (MCI) was purchased from Beijing Lvbaicao Technology Development Co., Ltd. (Beijing, China). The reagents (industrial grade) used in the column chromatography process were purchased from Yunnan Liyan Technology Co., Ltd. (Kunming, China).

### 2.3. Plant Material

The voucher specimen (52,860) of *C. fascicularis* was identified by taxonomist Min Tianlu and preserved in the Herbarium of Kunming Institute of Botany, Chinese Academy of Sciences. The leaves used in this study were collected from Dawei Mountain Nature Reserve in Hekou County, Yunnan Province, China, in December 2019 and were confirmed as *C. fascicularis* by Prof. Xiang Jianying of Southwest Forestry University.

### 2.4. Extraction and Isolation

**Figure 1:**
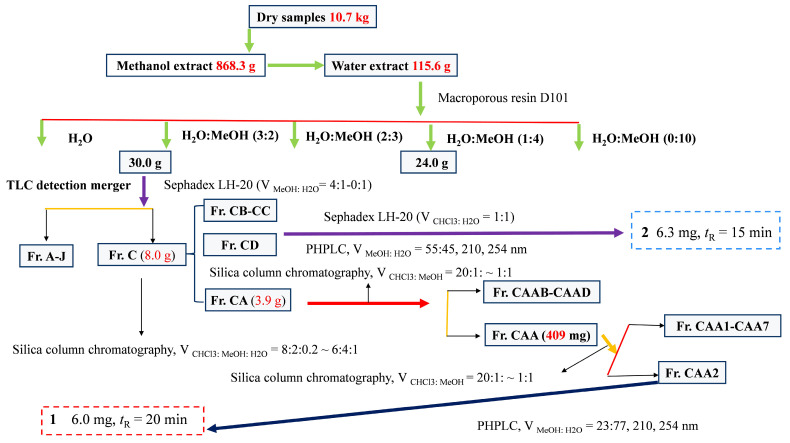
Diagram for the isolation and purification of compounds **1** and **2**.

In total, 10.7 kg of dried *C. fascicularis* samples were ground to approximately 40 mesh and extracted with 95% methanol (100 L, synergies are exploited to further improve extraction efficiency) under stirring at 50 °C) for 3, 2, and 1 h, respectively. This will increase the solubility and diffusion rate of the solvent, thereby increasing the extraction rate. After combining the extracts and evaporating the solvents under reduced pressure at 50 °C, we obtained the *C. fascicularis* methanolic extract (868.3 g). The extract was thoroughly mixed with 5 L distilled water and extracted three times using an equal volume of industrial-grade ethyl acetate [5]. The resulting ethyl acetate phase extract (177.8 g) was obtained via low-pressure rotary evaporation at 40 °C. Following the concentration of the remaining aqueous phase under high temperature and low pressure, the *C. fascicularis* aqueous phase extract (115.6 g) was acquired. The separation and purification flowchart for compounds **1** and **2** are illustrated in Figure 1.

The ethyl acetate phase extract was combined with 267.0 g of Macroporous resin (MR) D101 and loaded onto the column, followed by elution using a gradient of CH_3_OH:H_2_O (0:1 → 1:0). The resulting fractions were pooled into four fractions (Fr. I–IV) by thin-layer chromatography (TLC) analysis. After conducting preliminary screening for TLC and antioxidative activity, Fr. II–III exhibited superior TLC site formation and stronger antioxidative activity compared with the other fractions: DPPH, half-maximal inhibitory concentration [IC_50_] 69.44 ± 2.92/µg/mL; and ABTS, IC_50_ 151.23 ± 12.75/µg/mL [6]. The combined portion of 45.0 g (Fr. II–III) of the extract was subjected to Silica gel (SG) column chromatography using a CHCl_3_:CH_3_OH gradient elution system (1:0 → 0:1). Eight fractions (Fr. A–I) were collected using TLC analysis and pooled as part of the combined fractions [5].

Fr. A (V_CHCl3:MeOH_ = 50:1) SG eluted (6.0 g). The separation was conducted using MCI reversed-phase column chromatography with gradient elution of CH_3_OH–H_2_O (*v*/*v* 2:3, 1:1, 3:2, 7:3, 4:1, 9:1, 10:0), resulting in the collection of nine flow Fr. (Aa–Ai). Fr. Aa underwent further purification to remove impurities using Sephadex LH−20 (CH_2_Cl_2_:CH_3_OH = 95:5), succeeded by separation via elution using SG (CH_2_Cl_2_:CH_3_OH). The final purification was achieved using preparative high-performance liquid chromatography (PHPLC), resulting in the isolation of monomeric compound **3** (7.4 mg, V_MeOH:H2O_ = 26:74, *t*_R_ = 25 min). Fr. Ab underwent further purification to remove impurities using Sephadex LH−20 (CH_2_Cl_2_:CH_3_OH = 95:5), succeeded by separation via elution SG (CH_2_Cl_2_:CH_3_OH). The final purification was conducted using PHPLC, resulting in the isolation of monomeric compounds **4** (4.7 mg, V_MeOH:H2O_ = 31:69, *t*_R_ = 25 min), **5** (5.7 mg, V_MeOH:H2O_ = 31:69, *t*_R_ = 32 min), **6** (5.3 mg, V_MeOH:H2O_ = 35:65, *t*_R_ = 22 min). Fr. Ac underwent further purification to remove impurities using Sephadex LH−20 (CH_2_Cl_2_:CH_3_OH = 95:5), succeeded by separation via elution using SG (CH_2_Cl_2_:CH_3_OH). The final purification was conducted using PHPLC, resulting in the isolation of monomeric compounds **7** (2.3 mg, V_MeOH–H2O_ = 35–95, *t*_R_ = 16 min), **8** (2.1 mg, V_MeOH–H2O_ = 35–95, *t*_R_ = 19 min). Fr. A was combined to produce Fraction A* (2.2 g), which underwent further purification to eliminate impurities using Sephadex LH–20 (CH_2_Cl_2_:CH_3_OH = 1:1). This process yielded compounds **9** (8.7 mg), **10** (6.1 mg), and **11** (4.3 mg).

Fr. G (V_CHCl3:MeOH_ = 2:1) SG eluted (9.0 g). The separation was performed using MCI reversed-phase column chromatography with a gradient elution of CH_3_OH–H_2_O (*v*/*v* 2:3, 1:1, 3:2, 7:3, 4:1, 9:1, 10:0), resulting in the collection of nine fractions (Ga–Gi). Fr. Gi underwent further purification to remove impurities utilizing Sephadex LH–20 (CH_2_Cl_2_:CH_3_OH = 1:1), succeeded by separation via elution using SG (CH_2_Cl_2_:CH_3_OH). The final purification was conducted using PHPLC, yielding monomeric compounds **12** (7.6 mg, V_MeOH:H2O_ = 86:14, *t*_R_ = 22 min), **13** (1.7 mg, V_MeOH:H2O_ = 83:17, *t*_R_ = 15 min), **14** (1.2 mg, V_MeOH:H2O_ = 83:17, *t*_R_ = 16 min).

Fr. F (V_CHCl3:MeOH_ = 5:1) SG eluted (5.0 g). The separation was conducted using MCI reversed-phase column chromatography with a gradient elution of CH_3_OH–H_2_O (*v*/*v* 2:3, 1:1, 3:2, 7:3, 4:1, 9:1, 10:0), resulting in the collection of nine fractions (Fa–Fi). Fr. Ff underwent further purification to remove impurities utilizing Sephadex LH−20 (CH_2_Cl_2_:CH_3_OH = 95:5), succeeded by separation via elution using SG (CH_2_Cl_2_:CH_3_OH) to yield compound **15** (3.9 mg). The final purification was performed using preparative PHPLC, resulting in the isolation of monomeric compound **16** (3.1 mg, V_MeOH:H2O_ = 68:32, *t*_R_ = 15 min).

Fr. B (V_CHCl3:MeOH_ = 30:1) SG eluted (2.0 g). Further purification was performed using Sephadex LH−20 (CH_2_Cl_2_:CH_3_OH = 95:5), resulting in the removal of impurities and yielding seven fractions (Fr. Ba–Bf). Fr. Be was subsequently purified by PHPLC, leading to the isolation of monomeric compound **17** (2.3 mg, V_MeOH:H2O_ = 26:74, *t*_R_ = 21 min).

**Figure 2:**
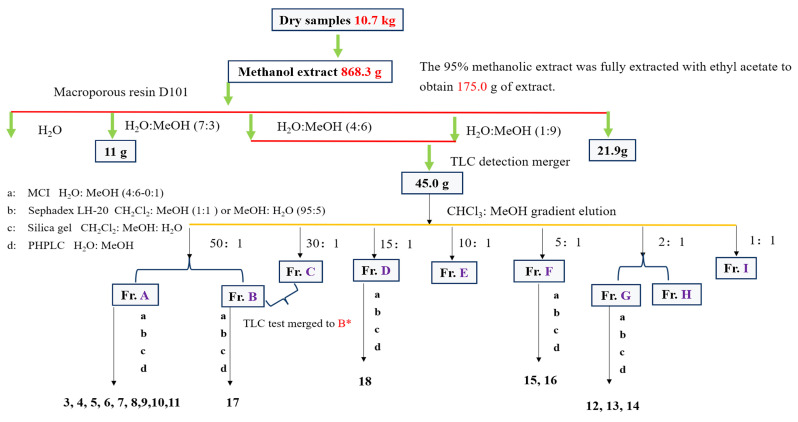
Diagram for the isolation and purification of compounds **3**–**18**.

Fr. D (V_CHCl3:MeOH_ = 15:1) SG eluted (4.0 g), Separation was achieved through MCI reversed-phase column chromatography with a gradient elution of CH_3_OH–H_2_O (*v*/*v* 2:3, 1:1, 3:2, 7:3, 4:1, 9:1, 10:0), resulting in the collection of eight fractions (Da–Dh). Fr. Dc underwent further purification to remove impurities utilizing Sephadex LH−20 (CH_2_Cl_2_:CH_3_OH = 95:5), succeeded by separation via elution using SG (CH_2_Cl_2_:CH_3_OH). The final purification was conducted using PHPLC, yielding monomeric compound **18** (3.8 mg, V_MeOH:H2O_ = 43:57, *t*_R_ = 14 min). A flow chart illustrating the separation and purification processes for compounds **3**–**18** is presented in Figure 2. Following the isolation and purification steps of secondary metabolites, and after confirming that the TLC results show a single clear spot, the organic solvent is evaporated off and the substance is finally weighed accurately. Select a suitable deuterated reagent to dissolve and send for testing, using tetramethylsilane (TMS) as the internal standard. NMR spectroscopy and HR-MS were conducted as described by Li et al. [6].

### 2.5. Bioactivity-Guided Analysis

#### 2.5.1. Antioxidative and Antibacterial Activities

The methods and screening results for antioxidative and antibacterial activities screening of the secondary metabolites in *C. fascicularis* were performed as described previously [6].

#### 2.5.2. Anti-Inflammatory Activity

The anti-inflammatory activity of Fr. (A–I) was evaluated using a method established before, with some modifications [18,19]. In a 96-well microplate, the reaction mixture was prepared by combining 100 μL of the sample, 50 μL of egg albumin (from fresh hen eggs), and 100 μL of phosphate-buffered saline at pH 7.2 (0.1 M). The reaction mixture was incubated at 37 °C for 15 min, followed by heat treatment at 70 °C for 6 min to terminate the reaction. Absorbance was measured at a wavelength of 600 nm using a microplate reader. A standard curve was generated using diclofenac sodium, with final concentrations ranging from 50 to 2500 μg/mL. The results were reported as diclofenac sodium equivalent capacity (DSEC, μg/mL) of the sample.

### 2.6. Activitive of Secondary Metabolites

#### 2.6.1. Antioxidative Activity

All isolated compounds were evaluated for ABTS free radical-scavenging capacity using a previously established method with minor adaptations [5,20]. The study utilized a 96-well plate, with each well holding 210 µL. Equal amounts of ABTS solution (7 mM) and potassium persulfate solution (5 mM) were combined and allowed to react in the dark at room temperature for 12 h to produce the ABTS radical cation. This mixture was subsequently diluted with anhydrous methanol to reach an absorbance of about 0.7 ± 0.02 units at a wavelength of 734 nm. Following this, 180 µL of the ABTS working solution was added into each well, along with samples (30 µL) of varying concentrations (10.0–500.0 µg/mL). After mixing thoroughly, the samples were incubated for six minutes at room temperature, away from light exposure. The absorbance readings were taken at 734 nm using a microplate reader, ensuring that results came from no fewer than three independent trials. Ascorbic acid acted as the positive control; DMSO served as the blank by replacing sample solutions, while absolute methanol substituted for the ABTS solution as a control.

#### 2.6.2. Antimicrobial Activity

The antimicrobial activity of all isolated secondary metabolites was assessed using slightly modified established methods [5,21]. Preparation of the antibacterial compound solution involves dissolving the drug in DMSO to reach a concentration of 500 µg/mL. For bacterial assays, the frozen bacteria stored at −80 °C was thawed at room temperature and then cultured overnight in sterilized Nutrient Broth (NB) medium at 37 °C with shaking. Then, 2 mL of this overnight culture was inoculated into fresh NB medium and incubated at 37 °C until A _600_ = 0.5; subsequently, this culture was diluted by a factor of 100 with NB medium and set aside. In the microdilution process, a sterile 96-well plate was used; 75 µL of NB dilution was added to wells A2–A11 and 75 µL of the compound solution was placed into wells A1–A2. Gradient dilutions were conducted, starting from well A2; an eight-channel pipette was used to transfer 75 µL from each dilution into the plate so that final concentrations varied between 500 and 1.95 µg/mL. The plates were incubated containing inoculated samples at 37 °C for an initial period of 12 h followed by another observation phase lasting an additional 16 h; MIC was determined by measuring A _600_ using an enzyme marker.

#### 2.6.3. Molecular Docking

The molecular docking technique is applicable in modeling the atomic-level interaction between a small molecule and a protein [22]. The structures of COX-2 (PDB ID: 8ET0) and α-glucosidase (PDB ID: 3A4A) were sourced from the Online Protein Data Bank (https://www.rcsb.org/, Date of access: 10 August 2024). The 3D structures of the ligands were generated using ChemBio3D Ultra 12.0.

Complexed ligands: Water molecules in the crystal structures of COX-2 and α-glucosidase were virtually removed using PyMOL Win (PyMOL 2.6.3, version: 2.4.0, DeLano Scientific LLC, New York, NY, USA). Gasteiger charges and essential hydrogen atoms were added using AutoDock tools (version: 1.5.6). The semi-flexible docking mode was used, and docking was performed 20 times. The affinity value (kcal/mol) indicates the binding capacity of the two interactions, with lower values signifying a more stable ligand-receptor binding. Dexamethasone (DXMS) and DSEC were designated as positive controls for docking anti-inflammatory compounds, while acarbose was selected as a control for docking hypoglycemic agents. PyMOL was used for visualization. 2D plots were visualized using the Discovery Studio 2020 Client (BIOVIA, San Diego, CA, USA) download for construction and analysis.

### 2.7. Statistical Analysis

Statistical analysis was performed using IBM SPSS Statistics 27. All experiments were performed in triplicate. The data obtained were analyzed using analysis of variance at a 95% confidence level (*p* ≤ 0.05). The results are expressed as the mean ± standard deviation (SD).

## 3. Results and Discussion

### 3.1. Bioactivity-Guided Evaluations

#### 3.1.1. Screening for Antioxidative and Antibacterial Activities

Antioxidative activity was evaluated using the DPPH, ABTS, and FRAP assays, whereas antimicrobial activity was assessed through MIC values against *Pseudomonas aeruginosa*, *Escherichia coli*, and *Staphylococcus aureus*. The bioactivity-guided isolation results indicated that Fr. A–G of the *C. fascicularis* ethanolic extracts may contain primary antioxidants. Furthermore, Fr. D–I exhibited antimicrobial properties [6].

#### 3.1.2. Screening for Anti-Inflammatory Activity

Excessive reactive oxygen species contribute to protein and DNA damage, leading to inflammation or mutations [23]. Furthermore, inflammation induces the release of multiple mediators and the production of cytokines. This progression causes direct injury to cells and tissues [24]. The use of glucocorticoids may cause certain adverse effects, which can be observed with some anti-inflammatory inhibitors [25]. Thus, active and safe antioxidative and anti-inflammatory inhibitors for the treatment of diseases must be identified. We herein evaluated the anti-inflammatory activity of the EtOAc extract of *C. fascicularis* and Fr. A–I (500 μg/mL) using assays evaluating in vitro anti-inflammatory activity. We used the DSEC standard curve regression equation and correlation coefficient of y = 0.5915x + 0.5217 (*R*^2^ = 0.9976). The DSEC mass concentration ranged from 50 to 2500 μg/mL. The absorbance showed good linear correlation. The anti-inflammatory results are shown in Table 1. The order of fractions by the strength of anti-inflammatory activity was Fr. A > E > H > G > B* > F > I > D. The anti-inflammatory activities of the other fractions were also more prominent than those of *C. fascicularis* EtOAc extract.

Interestingly, Fr. A–G of *C. fascicularis* EtOAc extract may contain the predominant antioxidative component, which is consistent with the observed anti-inflammatory effects and indicates a potential association with active constituents, such as phenolic acids, flavonoids, lignans, and terpenoids [6]. Notably, Fr. D exhibited the highest antioxidative activity and the least anti-inflammatory activity, which indicates the presence of intriguing active components that warrant further investigation.

### 3.2. Analysis of Secondary Metabolites

As confirmed by previous studies, the primary active constituents in *C. fascicularis* are predominantly present in the aqueous and ethyl acetate extracts. The ethyl acetate and aqueous fractions from the ethanolic extract were separated and purified using MR D101, SG, MCI, Sephadex LH–20, and PHPLC. The structures of secondary metabolites were determined by NMR and HR-MS analyses in conjunction with relevant literature. Of these metabolites, compounds **1** and **2** were isolated from the aqueous extract, while compounds **3**–**17** were obtained from the ethyl acetate extract. The extracted compounds included three phenylpropanoids (**1**, **15**, and **16**), a saponin (**2**), five terpenoids (**3**–**5**, **7**, and **8**), four phenolic acids (**6** and **9**–**11**), three phthalates (**12**–**14**), a fatty acid (**17**), and a lignin (**18**). Compounds **1**, **3**–**5**, **7**, and **15**–**18** were isolated from the genus *Camellia* for the first time, while the remaining compounds were isolated from *C. fascicularis* for the first time. The main chemical constituents isolated and identified from *C. fascicularis* include phenylpropanoids (**1**, **15**, **16**, **23**, **25**–**27**, and **52**), terpenoids (**3**–**5**, **7, 19**–**21**, **42**, and **55**–**57**), triterpene and saponins (**2**, **62**, and **65**), indole (**24**), phthalates (**12**–**14**), phenolic acids (**6**, **9**–**11**, **22**, **28**, **29**, **47**–**51, 53**, **54**, **61**, and **64**), fatty acid (**17**), flavonoids and flavonoid glycosides (**30**–**40**, **58**–**60**, and **67**), galactosylglycerol derivatives (**41**), and lignans (**18**, **43**–**46**, **63**, and **66**) [4,5,6]. Compounds **12**–**14** are phthalic acid components, which are unlikely to be of natural origin (from plants), and we considered them as isolates since the experimental procedure did not involve exposure to plastics, and such components have been identified in gas chromatography–mass spectrometry studies of *Camellia sinensis*. Other studies have also previously isolated the analogs of these compounds [26,27].

**Figure 3:**
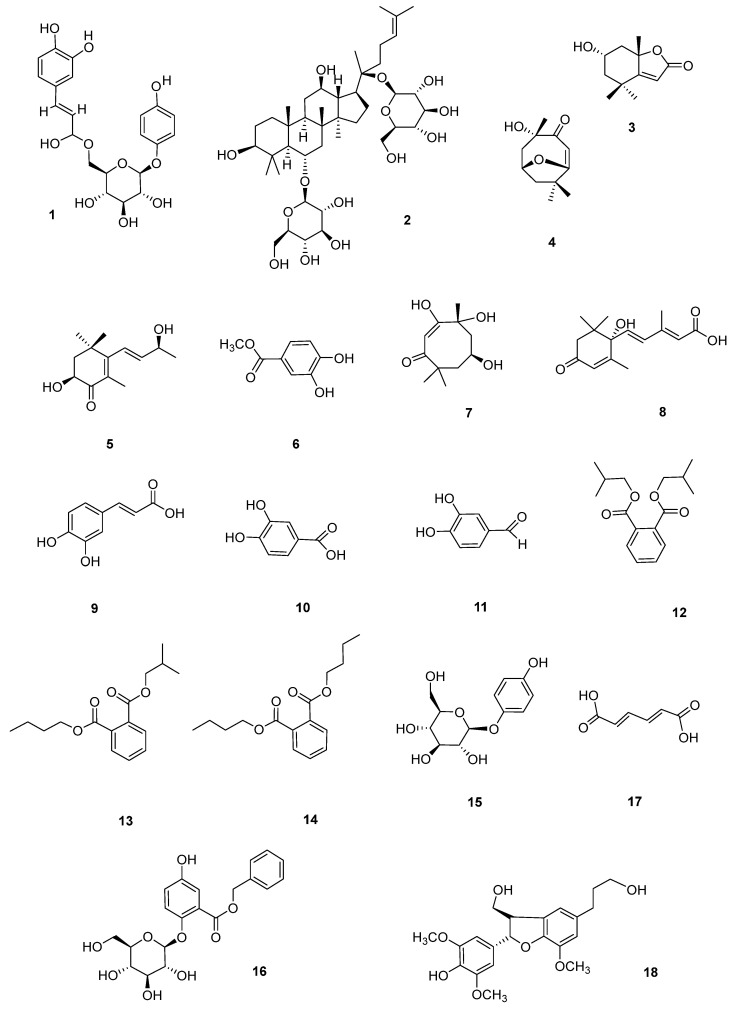
Structures of compounds **1**–**18**.

The structures of the identified compounds (Figure 3) were identified as 6′-*O*-caffeoylarbutin (CA) (**1**) [28], ginsenoside Rg1 (**2**) [29], (+)-epiloliolide (**3**) [30], pubinernoid A (**4**) [31], 3,9-dihydroxy-5,7-megastigmadien-4-one (**5**) [32], protocatechuic acid methyl ester (**6**) [33], chakyunglupulin B (**7**) [34], (2-*trans*,4-*trans*)-abscisic acid (**8**) [35], caffeic acid (**9**) [36], protocatechuic acid (**10**) [37], 3,4-dihydroxybenzaldehyde (**11**) [38], diisobutyl phthalate (**12**) [39], phthalic acid butyl isobutyl ester (**13**) [40], dibutyl phthalate (**14**) [41], arbutin (**15**) [42], trichocarpin (**16**) [43], adiadienedioic acid (**17**) [44], and (7*S*,8*R*)-3,3′,5-trimethoxy-4′,7-epoxy-8,5′-neolignan-4,9,9′-triol (**18**) [45], respectively. The NMR spectrum can be found in the Supplementary Materials.

CA (**1**): Amorphous powder, HRESIMS *m*/*z* 457.1180 [M + Na]^+^ (calcd for C_21_H_22_O_10_). ^1^H NMR (500 MHz, Methanol-*d*_4_) δ 7.54 (H-b, d, *J* = 15.8 Hz, 1H), 7.02 (H-2, d, *J* = 2.1 Hz, 1H), 6.91 (H-6, 2″, 6″, d, *J* = 8.9 Hz, 3H), 6.76 (H-5, d, *J* = 8.1 Hz, 1H), 6.62 (H-3″, 5″, d, *J* = 8.9 Hz, 2H), 6.25 (H-a, d, *J* = 15.9 Hz, 1H), 4.69 (H-1′, d, *J* = 7.1 Hz, 1H), 4.49 (H-6′b, d, *J* = 11.9 Hz, 1H), 4.31 (H-6′a, dd, *J* = 11.9, 6.6 Hz, 1H), 3.65–3.58 (H-5′, m, 1H), 3.44–3.36 (H-2′, 3′, 4′, m, 3H). ^13^C NMR (126 MHz, Methanol-*d*_4_) δ 169.3 (C-9′), 154.2 (C-1), 152.7 (C-4), 150.0 (C-3″), 147.6 (C-7″), 147.2 (C-4″), 128.0 (C-1″), 123.5 (C-1′), 119.9 (C-2, 6), 116.9 (C-3, 5), 116.8 (C-5″), 115.4 (C-2″), 115.1 (C-8″), 104.0 (C-1′), 78.1 (C-3′), 75.8 (C-2′), 75.3 (C-5′), 72.1 (C-4′), 64.9 (C-6′).

Ginsenoside Rg1 (**2**): White powder, HRESIMS *m*/*z* 799.4832 [M − H]^–^ (calcd for C_42_H_72_O_14_). ^1^H NMR (500 MHz, Methanol-*d*_4_) δ 5.14–5.10 (H-24, m, 1H), 4.62 (H-1″, d, *J* = 7.8 Hz, 2H), 4.37 (H-1′, d, *J* = 7.8 Hz, 1H), 1.70 (H-26, s, 3H), 1.64 (H-27, s, 3H), 1.36 (H-21, s, 3H), 1.34 (H-28, s, 3H), 1.11 (H-18, s, 3H), 1.02 (H-19, s, 3H), 1.01 (H-18, s, 3H), 0.97 (H-29, s, 3H). ^13^C NMR (126 MHz, Methanol-*d*_4_) δ 132.3 (C-25), 125.9 (C-24), 105.6 (C-1′), 98.3 (C-1″), 84.9 (C-20), 80.9 (C-6), 79.9 (C-3′), 79.1 (C-3″), 78.2 (C-5′), 77.9 (C-5″), 77.7 (C-3), 75.5 (C-2′), 75.4 (C-2″), 71.9 (C-4′), 71.7 (C-4″), 71.2 (C-12), 62.9 (C-6″), 62.5 (C-6′), 61.8 (C-5), 53.1 (C-7), 52.4 (C-14), 50.6 (C-13), 45.3 (C-7), 41.9 (C-8), 40.5 (C-4), 40.4 (C-10), 40.2 (C-1), 36.6 (C-22), 31.5 (C-28), 31.4 (C-11), 30.9 (C-15), 27.6 (C-6), 27.2 (C-16), 25.9 (C-2), 24.2 (C-23), 22.8 (C-21), 17.9 (C-27), 17.8 (C-19), 17.6 (C-18), 17.1 (C-30), 16.1 (C-29).

(+)-Epiloliolide (**3**): Colorless needle crystal, HRESIMS *m*/*z* 197.1181 [M + H]^+^ (calcd for C_11_H_16_O_3_). ^1^H NMR (500 MHz, Methanol-*d*_4_) δ 5.79 (H-3, s, 1H), 4.15–4.06 (H-6, m, 1H), 2.51–2.44 (H-7b, m, 1H), 2.04–1.98 (H-5b, m, 1H), 1.60 (H-8, s, 3H), 1.40 (H-7a, t, *J* = 11.7 Hz, 1H), 1.34 (H-5a, s, 1H), 1.32 (H-9, s, 3H), 1.29 (H-9, s, 3H), 1.21 (H-10, d, *J* = 6.4 Hz, 1H). ^13^C NMR (126 MHz, Methanol-*d*_4_) δ 184.4 (C-3a), 174.5 (C-2), 114.2 (C-3), 89.1 (C-7a), 65.8 (C-6), 51.2 (C-5), 50.1 (C-7), 36.7 (C-4), 30.8 (C-8), 26.2 (C-9), 25.8 (C-10).

Pubinernoid A (**4**): Amorphous powder, HRESIMS *m*/*z* 197.1238 [M + H]^+^ (calcd for C_11_H_16_O_3_). ^1^H NMR (500 MHz, Methanol-*d*_4_) δ 5.75 (H-6, s, 1H), 4.26–4.15 (H-2, m, 1H), 2.43 (H-3β, dt, *J* = 13.4, 2.6 Hz, 1H), 2.00 (H-1β, dt, *J* = 14.4, 2.6 Hz, 1H), 1.76 (H-3α, H-11, s, 4H), 1.53 (H-1a, dd, *J* = 14.4, 3.7 Hz, 1H), 1.47 (H-9, s, 3H), 1.28 (H-10, s, 3H). ^13^C NMR (126 MHz, Methanol-*d*_4_) δ 183.6 (C-5), 172.8 (C-7), 111.7 (C-6), 87.3 (C-4), 65.8 (C-2), 46.3 (C-3), 44.8 (C-3), 35.6 (C-8), 29.4 (C-10), 25.8 (C-11), 25.4 (C-8).

3,9-Dihydroxy-5,7-megastigmadien-4-one (**5**): Colorless powder, HRESIMS *m*/*z* 225.1481 [M + H]^+^ (calcd for C_13_H_20_O_3_). ^1^H NMR (500 MHz, Methanol-*d*_4_) δ 6.25 (H-7, d, *J* = 16.2 Hz, 1H), 5.75 (H-8, ddd, *J* = 16.2, 5.5, 3.1 Hz, 1H), 4.42–4.35 (H-9, m, 1H), 4.33 (1H, dd, *J* = 13.8, 5.5 Hz, 1H), 2.06 (H-2_eq_, dd, *J* = 12.5, 5.5 Hz, 1H), 1.84 (H-13, s, 3H), 1.79 (H-2_ax_, dd, dd, *J* = 13.5, 12.5 Hz, 1H), 1.30 (H-10, 12, d, *J* = 6.7 Hz, 6H), 1.16 (H-11, d, *J* = 3.1 Hz, 3H). ^13^C NMR (126 MHz, Methanol-*d*_4_) δ 201.5 (C-4), 162.4 (C-6), 142.7 (C-8), 128.5 (C-5), 125.1 (C-7), 70.1 (C-3), 68.6 (C-9), 46.6 (C-2), 37.4 (C-1), 30.4 (C-12), 25.6 (C-11), 23.3 (C-10), 13.4 (C-13).

Protocatechuic acid methyl ester (**6**): White powder, HRESIMS *m*/*z* 167.0451 [M − H]^–^ (calcd for C_8_H_8_O_4_). ^1^H NMR (500 MHz, Methanol-*d*_4_) δ 7.41 (H-2, s, 1H), 7.39 (s, OH), 6.78 (H-6, dd, *J* = 8.8 Hz, 1H), 3.82 (OMe, s, 3H). ^13^C NMR (126 MHz, Methanol-*d*_4_) δ 168.5 (C-O), 151.4 (C-4), 145.9 (C-3), 123.3 (C-6), 122.2 (C-1), 117.1 (C-2), 115.5 (C-5), 51.9 (OMe).

Chakyunglupulin B (**7**): Amorphous powder, HRESIMS *m*/*z* 197.1266 [M − H_2_O + H]^+^ (calcd for C_11_H_18_O_4_). ^1^H NMR (500 MHz, Methanol-*d*_4_) δ 5.74 (H-2, s, 1H), 4.24–4.15 (H-6, m, 1H), 2.41 (H-5β, ddd, *J* = 13.4, 3.1, 2.2 Hz, 1H), 1.98 (H-7β, ddd, *J* = 14.5, 2.7, 2.3 Hz, 1H), 1.75 (H-5α, H-11, s, 4H), 1.52 (H-7α, dd, *J* = 14.4, 3.0, 2.6 Hz, 1H), 1.45 (H-9, s, 3H), 1.26 (H-10, s, 3H). ^13^C NMR (126 MHz, Methanol-*d*_4_) δ 185.4 (C-1), 174.1 (C-3), 113.0 (C-2), 88.6 (C-4), 66.9 (C-6), 47.6 (C-7α, β), 46.1 (C-5α, β), 36.9 (C-8), 30.7 (C-10), 27.1 (C-11), 26.6 (C-9).

(2-*trans*,4-*trans*)-Abscisic acid (**8**): White amorphous powder, HRESIMS *m*/*z* 265.1471 [M + H]^+^ (calcd for C_15_H_20_O_4_). ^1^H NMR (500 MHz, Methanol-*d*_4_) δ 7.74 (H-4, d, *J* = 16.1 Hz, 1H), 6.24 (H-5, d, *J* = 16.1 Hz, 1H), 5.96 (H-8, s, 1H), 5.74 (H-2, s, 1H), 2.53 (H-10a, d, *J* = 16.7 Hz, 1H), 2.18 (H-10b, d, *J* = 16.8 Hz, 1H), 2.02 (H-15, d, *J* = 1.2 Hz, 3H), 1.93 (H-14, d, *J* = 1.4 Hz, 3H), 1.06 (H-12, s, 3H), 1.04 (H-13, s, 3H). ^13^C NMR (126 MHz, Methanol-*d*_4_) δ 201.3 (C-9), 166.6 (C-1, 3), 150.5 (C-7), 137.9 (C-5), 129.8 (C-4), 127.7 (C-8), 120.7 (C-2), 80.9 (C-6), 51.4 (C-10), 43.5 (C-11), 25.1 (C-12), 23.7 (C-13), 21.7 (C-15), 20.3 (C-14).

Caffeic acid (**9**): Yellow powder, calcd for C_9_H_8_O_4_. ^1^H NMR (500 MHz, Methanol-*d*_4_) δ 7.54 (H-7, d, *J* = 15.5 Hz, 1H), 7.07 (H-2, d, *J* = 2.1 Hz, 1H), 6.91 (H-6, d, *J* = 8.3 Hz, 1H), 6.72 (H-5, d, *J* = 8.1 Hz, 1H), 6. 27 (H-8, d, *J* = 15.9 Hz, 1H). ^13^C NMR (500 MHz, Methanol-*d*_4_) δ 171.0 (C-9), 149.5 (C-4), 147.1 (C-7), 146.8 (C-3), 127.8 (C-1), 122.9 (C-6), 116.5 (C-5), 115.5 (C-8), 115.1 (C-2).

Protocatechuic acid (**10**): White powder, calcd for C_7_H_6_O_4_. ^1^H NMR (500 MHz, Methanol-*d*_4_) δ 7.45 (H-2, d, *J* = 2.1 Hz, 1H), 7.38 (H-6, dd, *J* = 8.3, 2.1 Hz, 1H), 6.91 (H-5, d, *J* = 8.2 Hz, 1H). ^13^C NMR (126 MHz, Methanol-*d*_4_) δ 170.4 (C-7), 151.7 (C-4), 146.2 (C-3), 124.1 (C-6), 123.3 (C-1), 117.9 (C-2), 115.9 (C-5).

3,4-Dihydroxybenzaldehyde (**11**): Pale yellow powder, calcd for C_7_H_6_O_3_. ^1^H NMR (500 MHz, Methanol-*d*_4_) δ 9.71 (H-7, s, 1H), 7.30 (H-2, d, *J* = 2.2 Hz, 1H), 7.28 (H-6, dd, *J* = 8.2, 1.9 Hz, 1H), 6.93 (H-5, d, *J* = 8.1 Hz, 1H). ^13^C NMR (126 MHz, Methanol-*d*_4_) δ 193.1 (CHO), 153.7 (C-4), 147.2 (C-3), 130.8 (C-1), 126.4 (C-6), 116.2 (C-2), 115.2 (C-5).

Diisobutyl phthalate (**12**): Pale yellow oil, HRESIMS *m*/*z* 279.1635 [M + H]^+^ (calcd for C_16_H_22_O_4_). ^1^H NMR (500 MHz, Methanol-*d*_4_) *δ* 7.73 (H-2, 5, dd, *J* = 5.7, 3.3 Hz, 2H), 7.62 (H-3, 4, dd, *J* = 5.7, 3.3 Hz, 2H), 4.07 (H-1′, 1″, d, *J* = 6.6 Hz, 4H), 2.36–2.25 (H-2″, m, 1H), 2.08–2.00 (H-2′, m, 1H), 0.99 (H-3′, 3″, 4′, 4″, d, *J* = 6.7 Hz, 12H). ^13^C NMR (126 MHz, Methanol-*d*_4_) *δ* 169.3 (C-7, 8), 133.6 (C-1, 6), 132.4 (C-3, 4), 129.9 (C-2, 5), 72.9 (C-1′, 7″), 29.0 (C-2′, 2″), 19.5 (C-3′, 3″).

Phthalic acid butyl isobutyl ester (**13**): Pale yellow oil, HRESIMS *m*/*z* 301.1406 [M + Na]^+^ (calcd for C_16_H_22_O_4_). ^1^H NMR (500 MHz, Methanol-*d*_4_) *δ* 7.73 (H-3, 6, dd, *J* = 5.7, 3.3 Hz, 2H), 7.62 (H-4, 5, dd, *J* = 5.7, 3.3 Hz, 2H), 4.30 (H-1′, 1″, s, 4H), 1.73 (H-2″, dd, *J* = 8.6, 6.3 Hz, 2H), 1.49–1.42 (H-3″, m, 2H), 0.99 (H-3′, 4′, t, *J* = 7.4 Hz, 6H), 0.91 (H-4″, t, *J* = 6.7 Hz, 3H). ^13^C NMR (126 MHz, Methanol-*d*_4_) *δ* 169.3 (C-1a, 2a), 133.6 (C-1), 132.4 (C-2),131.1 (C-4, 5), 129.9 (C-3, 6), 72.9 (C-1′), 65.8 (C-1″), 30.8 (C-2″), 30.6 (C-3″), 28.9 (C-2′), 19.5 (C-3′, 5′), 14.4 (C-4″).

Dibutyl phthalate (**14**): Pale yellow oil, HRESIMS *m*/*z* 301.1476 [M + Na]^+^ (calcd for C_16_H_22_O_4_). ^1^H NMR (500 MHz, Methanol-*d*_4_) *δ* 7.73 (H-3, 6, dd, *J* = 5.7, 3.3 Hz, 2H), 7.62 (H-4, 5, dd, *J* = 5.7, 3.3 Hz, 2H), 4.07 (H-3′, 3″, d, *J* = 6.6 Hz, 4H), 1.73 (H-4′, 4″, m, *J* = 8.6, 6.3 Hz, 4H), 1.49–1.42 (H-5′, 5″, m, 4H), 0.99 (H-6, 6′, t, *J* = 7.4 Hz, 6H). ^13^C NMR (126 MHz, Methanol-*d*4) *δ* 169.5 (C-1′), 133.8 (C-4), 132.5 (C-1), 130.0 (C-3), 66.8 (C-3′), 31.9 (C-4′), 20.4 (C-5′), 14.2 (C-6′).

Arbutin (**15**): White powder, HRESIMS *m*/*z* 273.0792 [M + H]^+^ (calcd for C_12_H_16_O_7_). ^1^H NMR (500 MHz, Methanol-*d*_4_) δ 6.98 (H-2, 6, d, *J* = 8.9 Hz, 1H), 6.71 (H-3, 5, d, *J* = 8.9 Hz, 2H), 4.75 (H-1′, d, *J* = 7.4 Hz, 1H), 3.94–3.35 (6′α, 6′β, m, H-2′-5′, 6H). ^13^C NMR (126 MHz, Methanol-*d*_4_) δ 153.9 (C-4), 152.6 (C-1), 119.6 (C-3, 5), 116.8 (C-2, 6), 103.8 (C-1′), 78.2 (C-3′), 78.2 (C-5′), 75.2 (C-2′), 71.6 (C-4′), 62.7 (C-6′).

Trichocarpin (**16**): Yellow powder, calcd for C_20_H_22_O_9_. ^1^H NMR (500 MHz, Methanol-*d*_4_) δ 7.60 (H-6, d, *J* = 3.0 Hz, 1H), 7.49 (H-2′, 6′, d, *J* = 7.0 Hz, 2H), 7.43 (H-3′, 5′, d, *J* = 7.3 Hz, 2H), 7.38 (H-4′, t, *J* = 7.1 Hz, 1H), 7.35 (H-4, dd, *J* = 9.1, 3.1 Hz, 1H), 6.93 (H-3, d, *J* = 9.1 Hz, 1H), 5.42 (Hb-7′, s, 2H), 4.73 (H-1″, d, *J* = 7.3 Hz, 1H), 3.74 (Ha-6″, d, *J* = 9.6 Hz, 1H), 3.61 (Hb-6″, dd, *J* = 11.2, 5.8 Hz, 1H), 3.50–3.36 (H-2′, 3′ 4′ 5′, m, 4H). ^13^C NMR (126 MHz, Methanol-*d*_4_) δ 170.9 (C-7), 158.4 (C-5), 151.6 (C-2), 137.2 (C-1′), 129.9 (C-2′,6′), 129.7 (C-4′), 129.6 (C-3′), 129.5 (C-5′), 119.4 (C-3), 118.9 (C-6), 113.6 (C-1), 111.1 (C-1′), 103.8 (C-1″), 78.2 (C-5″), 78.0 (C-3″), 75.2 (C-2″), 71.5 (C-4″), 68.6 (C-7′), 63.2 (C-6″).

Adiadienedioic acid (**17**): White powder, HRESIMS *m*/*z* 141.0584 [M–H]^–^ (calcd for C_6_H_6_O_4_). ^1^H NMR (500 MHz, Methanol-*d*_4_) δ 7.88 (H-3, 4, d, *J* = 8.9 Hz, 2H), 6.78 (H-2, 5, d, *J* = 8.9 Hz, 2H). ^13^C NMR (126 MHz, Methanol-*d*_4_) δ 163.4 (C-1, 6), 133.2 (C-3, 4), 115.8 (C-2, 5).

(7*S*,8*R*)-3,3′,5-trimethoxy-4′,7-epoxy-8,5′-neolignan-4,9,9′-triol (**18**): White powder, calcd for C_22_H_26_O_7_. ^1^H NMR (500 MHz, Methanol-*d*_4_) δ 6.79 (H-2, 6, s, 2H), 6.60 (H-2′, s, 1H), 6.59 (H-6′, s, 1H), 5.59 (H-7, d, *J* = 5.6 Hz, 1H), 3.88 (H-3′-OMe, s, 3H), 3.86 (H-9, s, 1H), 3.84 (H-3, 5-OMe, s, 6H), 3.57 (H-9′, t, *J* = 6.5 Hz, 2H), 3.47–3.45 (H-8, m, 1H), 2.57 (H-7′, t, *J* = 6.7 Hz, 2H), 1.92 (H-8′, s, 2H). ^13^C NMR (126 MHz, Methanol-*d*_4_) δ 155.2 (C-3, 5), 147.2 (C-4′), 143.8 (C-3′), 140.7 (C-1), 137.6 (C-1′), 136.8 (C-4), 129.8 (C-5′), 117.6 (C-6′), 104.3 (C-2, 6), 88.8 (C-7), 65.8 (C-9), 62.6 (C-9′), 59.9 (C-3′-OMe), 57.1 (C-3, 5-OMe), 56.6 (C-8), 36.3 (C-8′), 33.2 (C-7′).

### 3.3. Antioxidative Activity of Secondary Metabolites

Plants serve as a significant reservoir of bioactive secondary metabolites, while reactive oxygen species and free radicals are primary contributors to oxidative stress [46]. Using biological activities (anti-inflammatory, antioxidative, and antimicrobial) to guide the isolation of compounds, we further isolated 18 compounds (**1**–**18**) from *C. fascicularis*. Since 17 compounds (**19**–**35**) had been previously isolated from *C. fascicularis* but were not tested for bioactivity, their activities were further explored. The chemical structure of the compounds (**19**–**67**) are shown in the Supporting Materials.

The in vitro antioxidative activity of all the isolates was evaluated using the ABTS assay, and the results are presented in Table 2. Compounds **1**, **9**–**11**, **28**, **30**, and **31** showed comparable antioxidative activity to Ascorbic acid (AA), whereas the other compounds showed weak or insignificant antioxidative activity. These findings confirm that flavonoids [47,48,49] and phenolic acid [50] compounds have good antioxidative activities.

Compounds **30**–**32** are flavonoid components. Compound **32** exhibited weaker antioxidative activity than compounds **30** and **31**. Furthermore, compounds **33**–**35** are flavonoid glycoside components. Compound **34** demonstrated significantly stronger antioxidative activity than compounds **33** and **35**. Comprehensive investigation into the structural properties of these six flavonoids revealed a close correlation between the number of hydroxyl groups in the B-rings of compounds and their efficacy in scavenging free radicals. Notably, the increased number of hydroxyl groups correspondingly increased their antioxidative activity. The alcohol hydroxyl group at position C-3 exhibited superior stability and lower electron sensitivity than the phenol hydroxyl group. This unique characteristic enhanced water solubility while preserving antioxidative activity. These findings align with established research on flavonoid antioxidants and further deepen our understanding of the intricate relationship between structure and function [51,52,53]. In addition, differences in the antioxidative capacities of compounds **33** and **35** can be mainly attributed to variations in the quantity and arrangement of hydroxyl groups and to the spatial resistance at glycoside sites [54]. Compounds **1** (CA) and **15**, are arbutin compounds and thus have strong antioxidative activity. CA, a derivative of arbutin, is the phenolic compound with the highest content with a caffeoyl group in *Vaccinium dunalianum*. Compared with *β*-arbutin, CA has stronger melanin inhibitory activity, lower toxicity and higher safety and can be used as an alternative to *β*-arbutin [28,55]. The present research extends the plant sources of CA. In previous studies on *C. fascicularis*, an analog (6-*O*-Acetylcorydaline) was also found [56].

Compounds **6**, **9**–**11**, **22**, and **26**–**29** are phenolic acids derived from hydroxybenzoic acid as the core structure. Of these, compound **27** exhibited significantly lower antioxidative activity than the other compounds. In contrast, the introduction of *O*-hydroxyl and *O*-methoxy groups markedly enhanced the antioxidative properties of this class of phenolic acids [57]. The suboptimal antioxidative activity of compound **27** can be primarily ascribed to the absence of essential active sites within its structure, thereby limiting its effectiveness in antioxidative reactions. Compounds **3**–**5**, **7**, **8,** and **19**–**21** are terpenoids, whereas compounds **3** and **19** are carotenoid metabolites, which are potential endogenous herbivore resistance inducers. Compounds **5**, **8**, and **20** are *β*-ionone derivatives that exhibit some antioxidative activity and may be aromatic components and plant physiological regulators unique to *C. fascicularis*. Compounds **4**, **7**, and **21** have interesting structures, although their antioxidative activity is not very prominent, and their role in *C. fascicularis* remains unclear. A similar tetraterpene compound, solalyratin B, which was isolated in a previous study, has good antioxidative and antimicrobial activity. *C. fascicularis* possibly contains such or similar active ingredients [5]. Compound **2** belongs to the group of triterpenoid saponins with poor antioxidative activity, probably because of the introduction of sugar groups and substitution of the active site, thereby reducing the activity of the compound.

### 3.4. Antibacterial Activity of Secondary Metabolites

Plant extracts and their secondary metabolites are highly effective and widely available natural antimicrobial agents with great application value in the food industry [58]. Polysaccharides, phenols, alkaloids, terpenes, and other compounds in plant secondary metabolites are rich in antibacterial activities, which are realized through various chemical structures. Their main antibacterial mechanisms include destruction of bacterial structure, regulation of gene expression, inhibition of metabolic activity and alteration of cell membrane potential [59,60]. The assessment of antimicrobial activity revealed that compounds **1**–**35** demonstrated varying degrees of inhibition against *S. aureus*, *E. coli*, and *P. aeruginosa* at a concentration of 500 µg/mL (Table 3). Notably, compounds **3**, **17**, **19**, and **25** exhibited lower antimicrobial activity than the other compounds. Compounds **1** and **23** showed more pronounced inhibition against *E. coli* at a concentration range of 250–500 µg/mL, whereas all other compounds showed varying degrees of inhibition. Compound **15** showed superior inhibition against *S. aureus* compared with the other compounds. Compounds **7**, **18**, **22**, and **27** exhibited inhibitory activity against *P. aeruginosa* comparable to that of tetracycline (MIC: 125 µg/mL) and superior to that of penicillin (MIC: 250 µg/mL). The antimicrobial activity of most polyphenols is likely to depend on the interaction between the polyphenol and the bacterial cell surface. Polyphenols, as essential defense components in plants, are crucial in mitigating external threats and damage from parasites and pathogenic bacteria and serve as the fundamental material basis for the plant’s self-protection mechanisms [61,62]. An increase in rhamnose content in the plant significantly enhances the antimicrobial properties of polysaccharides; this may have enhanced the antimicrobial activity exhibited by compounds **1** and **16** within a specific concentration range [63]. Studies have also shown that the glycoside component is not important for antibacterial efficacy [64]. Compound **7** inhibited the growth of *P. aeruginosa* better than other terpene components. It may be that the formation of cyclic terpenes exhibits better antimicrobial activity than non-formed terpenes and is significantly affected by various substituents [65].

Flavonoids, phenols, and terpenoids were the major antimicrobial secondary metabolites of *C. fascicularis*. The antimicrobial performances of compounds **1**–**35** were in high agreement with the results of the antimicrobial activity screening experiments, especially the inhibitory effect on *P. aeruginosa*, which was more significant than that on *E. coli* or *S. aureus*. Notably, this method only provides an approximation of the MIC. While microscopic and scanning electron microscopic observations of bacterial structures would have provided deeper insights, these could not be performed because the bioactivity tests had exhausted all samples of the compounds. The SAR of secondary metabolites is complex, involving chemical structure, functional groups, substituents, molecular skeleton, configuration, size, and polarity. The antibacterial mechanism includes destroying bacterial structure, inhibiting metabolism and regulating gene expression. It is of great significance to study the antibacterial SAR of secondary metabolites of *C. fascicularis* to promote the development of antibacterial drugs, enrich the types of antibacterial agents, and promote the development of the food industry.

### 3.5. Anti-Inflammatory and Hypoglycemic Molecular Docking of Secondary Metabolites

Unfortunately, the samples of most of the compounds were depleted while evaluating antioxidative and antimicrobial activities; thus, their anti-inflammatory and hypoglycemic properties could only be predicted by molecular docking binding. Despite these limitations, molecular docking of the secondary metabolites of *C. fascicularis* with the proteins **8ET0** and **3A4A** is an innovative exploration that is expected to provide valuable reference data for future research. Computer-simulated molecular docking was performed to elucidate the interaction between the enzyme and potential ligand and determine the possible interaction mechanisms. Compounds **1**–**67** were molecularly docked for their anti-inflammatory and hypoglycemic binding energy (BE) (Table 4). Anti-inflammatory molecular docking revealed that the BEs of compounds **1**, **2**, **18**, **26**, **33**–**41**, **43**, **46**, and **63**–**67** were less than that of DXMS (−6.14 kcal/mol), whereas those of compounds **5**, **12**–**14**, **27**, **30**–**32**, **42**, **44**, **45**, and **57**–**60** were less than that of DSEC (−5.12 kcal/mol), signifying that these compounds exhibit good anti-inflammatory ability. Molecular docking of hypoglycemic molecules showed that the BEs of compounds **2**, **36**, and **65** were less than that of acarbose (−7.56 kcal/mol), indicating hypoglycemic activity superior to that of the positive control. In practice, BEs are often used to assess the degree of receptor–ligand affinity. In general, a BE less than –4.25, –5.0, or –7.0 kcal/mol indicates some good or strong ligand–receptor binding, respectively. 

**Figure 4:**
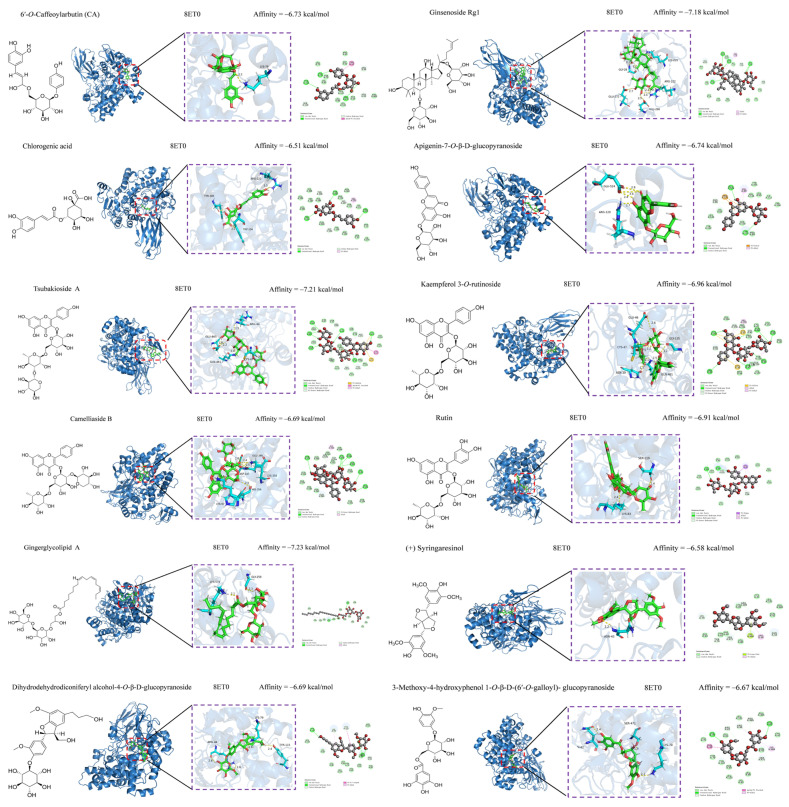
Anti-Inflammatory COX-2 (8ET0) molecular docking map (1).

**Figure 5:**
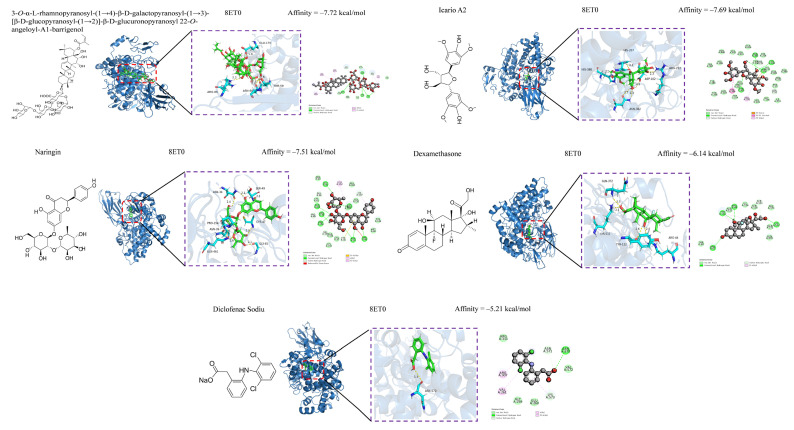
Anti-Inflammatory COX-2 (8ET0) molecular docking map (2).

**Figure 6:**
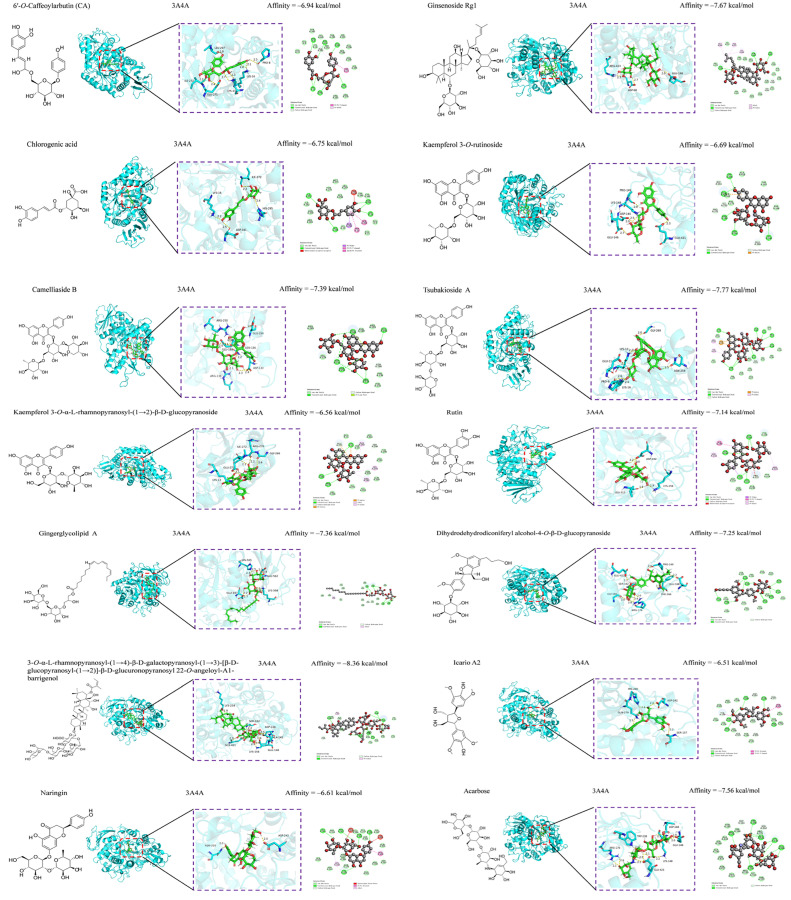
Hypoglycemic (3A4A) molecular docking map.

Oxidative stress, which involves free radicals and reactive metabolites, often results from elevated levels of free radicals or decreased levels of antioxidants [66]. Oxidation can adversely affect biomolecules, including DNA, lipids, and proteins, and is closely linked to various diseases, such as inflammation, aging, cancer, diabetes, Parkinson’s disease, and atherosclerosis [67,68]. Natural hypoglycemic components include polysaccharides, flavonoids, and alkaloids. We investigated the antioxidative and antimicrobial activities of compounds **36**–**67** and further explored their anti-inflammatory and hypoglycemic abilities in combination with compounds **1**–**35** using molecular docking (Table 4). A low BE indicates a high likelihood of receptor–ligand binding, high affinity, and greater stability. Compounds with BE > −6.5 kcal/mol were selected for anti-inflammatory and hypoglycemic molecular docking visualization, and after screening, anti-inflammatory had compounds **1**, **2**, **26**, **33**, **36**–**38**, **40**, **41**, **43**, and **63**–**67**, and hypoglycemic compounds had (**1**, **2**, **26**, **37**–**41**, and **63**–**67**). The results of the anti-inflammatory visualization are shown in Figure 4 and Figure 5, and the results of the hypoglycemic visualization are shown in Figure 6.

Hydroxyl groups (–OH) play a crucial role in the anti-inflammatory properties of flavones. The –OH at the C-5 and C-4′ positions enhance the activity of flavones, whereas the –OH at the C-6, C-7, C-8, and C-3′ positions attenuate it. Moreover, the C2-C3 single bond and –OH at the C-3 and B-ring positions undermine the activity of flavone aglycones [69,70,71]. The present study found that the flavonoid *O*-glycosides exhibited comparable binding affinities to COX-2, although they are lower in content than C-glycosides; this finding was consistent with that of previous studies [72,73]. The phenyl propionyl group is generally an anti-inflammatory group. The phenol antelope side chain double bond on the benzene ring enhances the anti-inflammatory ability of the phenyl propionyl group. Furthermore, its anti-inflammatory mechanism is mainly activated through the pathway acting on LO, COX, and proinflammatory cytokines [74,75]. Lignans are important components of plant phenolics. Compounds with a dioxolane ring moiety have higher anti-inflammatory ability in terms of conformational relationships [76,77]. The introduction of acyl groups to the parent nucleus of terpenoids improves anti-inflammatory activity and increases with the number of acyl groups and length of the acyl carbon chain. In addition, the type of substituents on the C ring of polycyclic terpenoids is the main source of the anti-inflammatory ability, which also explains the stronger anti-inflammatory ability of compound **65** than that of compound **62**. Other terpenoids have moderate anti-inflammatory ability [78,79]. Other types of compounds exert weaker anti-inflammatory ability. The results of the present study showed that active secondary metabolites, such as flavonoids, lignans, phenolic acids, saponins, and terpenoids, were the main anti-inflammatory components of *C. fascicularis*.

Compounds **2**, **36**, **38**, **40**, **41**, and **63** have comparable hypoglycemic effects to the positive control (acarbose, −7.56 kcal/mol), whereas that of compound **65** (−8.36 kcal/mol) is higher than that of the positive control. Constitutive relationships demonstrated that in terms of the hypoglycemic BE of the phenolic acid constituents, the additional methoxy group within the vicinity of the para-hydroxyl group of benzoic acid enhanced the inhibition of α-glucosidase (3A4A) but reduced the antioxidative activity, except in the para position [80]. The molecular docking of flavonoids and flavonoid glycosides revealed that adding sugar groups altered their physicochemical properties, increasing molar mass, polar surface area and volume, and molecular flexibility due to increased rotational and hydrogen-bonding sites. While such changes promoted favorable interactions with the α-glucosidase active site and enhanced the inhibitory potential, they weakened interactions with the flavonoid core (A, B, and C rings) and reduced the binding stability due to excessive flexibility [81,82]. Saponins are natural active substances that have multipath way and multitarget pharmacological characteristics. This study is the inaugural report on the hypoglycemic effect of compound **65**. Terpenoid and lignan constituents have a good hypoglycemic effect, which has also been reported previously [83,84,85]. Molecular docking showed that saponins, flavonoids and flavonoid glycosides, lignans, terpenoids, and phenolics were the major hypoglycemic constituents of *C. fascicularis.*

Docking is typically unable to rule out the possibility that a compound binds a target because the binding pocket could rearrange in a manner that is difficult to anticipate. This is acceptable given the typical goals of virtual screening but makes the direct application of docking difficult in identifying selective ligands that do not bind to other proteins related to the target [15]. Using docking to identify ligands that bind only to one conformational state of a target is also difficult [16]. In vitro and in vivo studies of the anti-inflammatory and hypoglycemic properties of the compounds identified would confirm our findings. Regrettably, all secondary metabolites were exhausted during the bioactivity assay, preventing any further analysis.

## 4. Conclusions

The present study investigated the bioactivity-guided isolation of active constituents from *C. fascicularis*. The results indicated that the anti-inflammatory, antioxidative, and antimicrobial active components were mainly found in Fr. B*, E, A, and H; Fr. A–G; and Fr. D–I, respectively. Bioactivity-guided isolation identified 18 secondary metabolites. Compounds **1, 3**–**5, 7**, and **15**–**18** were isolated from the genus *Camellia* for the first time in this study, whereas the other compounds were also isolated from this plant for the first time. Compounds **1**, **9**–**11**, **28**, **30**, and **31** demonstrated antioxidative activities comparable to those of ascorbic acid, whereas the other remaining compounds exhibited lower antioxidative activity. Compounds **7**, **18**, **22**, and **27** exerted antimicrobial inhibition against *P. aeruginosa* with a potency similar to that of tetracycline (MIC: 125 µg/mL). The other compounds showed moderate to weak inhibitory effects against *E. coli* and *S. aureus* (MIC: 250–500 µg/mL). Molecular docking revealed that compounds **2**, **36**, **41**, and **65** bound strongly to **8ET0**, whereas compounds **2**, **36**, **38**, **40**, **63,** and **65** bound strongly to **3A4A**. The results further indicated that *C. fascicularis* has good antioxidative, antibacterial, anti-inflammatory, and hypoglycemic effects. This research has clarified the various secondary metabolites of *C. fascicularis*, laying a material foundation for further research on the edible and medicinal value of this plant so that this precious resource can be better developed and utilized in the future.

## Figures and Tables

**Table 1:** Anti-Inflammatory activity in (Fr. A–I) of *C. fasciculata*.

Anti-Inflammatory Activity (DSEC μg/mL)				
**A**	B*	**D**	**E**	**F**
55.07 ± 9.75 ^a^	227.85 ± 31.48 ^e^	515.13 ± 38.24 ^h^	81.91 ± 10.64 ^b^	307.57± 38.62 ^f^
**G**	**H**	**I**	**Fr. II–III**	**EtOAc**
191.46 ± 26.54 ^d^	86.90 ± 13.76 ^bc^	341.93 ± 34.62 ^g^	489.26 ± 51.13 ^h^	529.63 ± 54.70 ^i^

“B*” was derived from the combination of fractions **B** and **C**. Values marked with different letters are statistically significant (*p* ≤ 0.05) (n = 5).

**Table 2:** ABTS of compounds **1**–**35** in *C. fascicularis*.

Compounds	ABTS ^B^ Assay (%)			
	500 µg/mL	100 µg/mL	50 µg/mL	10 µg/mL
**1**	98.68 ± 0.17 ^a^	–	90.85 ± 3.33 ^a^	24.22 ± 2.33 ^a^
**2**	9.11 ± 1.17 ^n^	–	–	–
**3**	61.78 ± 4.92 ^hi^	–	–	–
**4**	63.90 ± 2.37 ^h^	–	–	–
**5**	45.68 ± 2.34 ^k^	–	–	–
**6**	96.66 ± 0.56 ^b^	83.17 ± 4.27 ^d^	48.52 ± 1.27 ^f^	4.14 ± 0.82 ^e^
**7**	59.92 ± 2.82 ^hi^	–	–	–
**8**	52.34 ± 3.21 ^j^	–	–	–
**9**	100.28 ± 0.13 ^a^	99.87 ± 0.28 ^a^	71.04 ± 2.89 ^b^	18.78 ± 1.39 ^c^
**10**	99.30 ± 0.12 ^a^	93.67 ± 0.86 ^bc^	47.25 ± 2.54 ^f^	15.59 ± 2.23 ^d^
**11**	95.83 ± 0.32 ^b^	92.87 ± 1.57 ^bc^	57.46 ± 2.73 ^e^	16.15 ± 2.50 ^d^
**12**	24.20 ± 2.57 ^m^	–	–	–
**13**	46.38 ± 1.14 ^k^	–	–	–
**14**	42.63 ± 3.53 ^l^	–	–	–
**15**	89.01 ± 3.32 ^cd^	79.22 ± 1.97 ^de^	25.19 ± 2.02 ^g^	–
**16**	72.34 ± 1.69 ^f^	22.64 ± 3.69 ^j^	–	–
**17**	8.47 ± 1.11 ^n^	–	–	–
**18**	–	–	–	–
**19**	57.90 ± 1.37 ^i^	–	–	–
**20**	53.83 ± 2.69 ^j^	11.49 ± 1.66 ^m^	–	–
**21**	65.92 ± 3.92 ^g^	–	–	–
**22**	89.18 ± 1.97 ^cd^	–	–	–
**23**	92.12 ± 3.53 ^c^	15.29 ± 1.30 ^l^	–	–
**24**	86.55 ± 3.31 ^e^	36.25 ± 2.54 ^h^	–	–
**25**	8.81 ± 1.39 ^n^	–	–	–
**26**	99.16 ± 1.02 ^a^	70.73 ± 3.47 ^f^	21.52 ± 1.83 ^gh^	–
**27**	94.40 ± 1.18 ^b^	66.52 ± 2.62 ^g^	14.49 ± 2.16 ^i^	–
**28**	100.27 ± 0.63 ^a^	100.20 ± 0.11 ^a^	70.65 ± 1.94 ^b^	25.34 ± 1.57 ^a^
**29**	99.41 ± 1.69 ^a^	–	–	23.13 ± 1.61 ^ab^
**30**	99.52 ± 0.38 ^a^	93.67 ± 0.68 ^bc^	46.59 ± 0.72 ^f^	15.07 ± 2.90 ^d^
**31**	100.38 ± 0.47 ^a^	95.61 ± 2.13 ^b^	60.95 ± 2.00 ^d^	19.43 ± 1.84 ^c^
**32**	94.12 ± 0.99 ^b^	36.36 ± 1.64 ^h^	–	–
**33**	72.60 ± 2.58 ^f^	18.83 ± 2.45 ^k^	–	–
**34**	99.73 ± 0.59 ^a^	29.79 ± 2.54 ^i^	–	–
**35**	59.86 ± 2.91 ^hi^	18.92 ± 3.24 ^k^	–	–
**^A^** **AA**	-	98.35 ± 1.03 ^a^	65.12 ± 3.35 ^c^	23.00 ± 2.86 ^ab^

“A” means positive control; “B” means inhibition ratio; “–” indicates that the experiment has not been performed. Values marked with different letters are statistically significant (*p* ≤ 0.05) (n = 3).

**Table 3:** Antibacterial activity of compounds **1**–**35** in *C. fascicularis*.

Components	MIC ^b^ µg/mL		
	*E. coli*	*S*. *aureus*	*P. aeruginosa*
**1**	250.00	500.00	250.00
**2**	500.00	500.00	250.00
**3**	>500.00	>500.00	500.00
**4**	500.00	500.00	250.00
**5**	500.00	500.00	250.00
**6**	500.00	500.00	250.00
**7**	500.00	500.00	125.00
**8**	500.00	500.00	250.00
**9**	500.00	500.00	250.00
**10**	500.00	500.00	250.00
**11**	500.00	500.00	250.00
**12**	500.00	500.00	–
**13**	500.00	500.00	–
**14**	500.00	500.00	–
**15**	500.00	250.00	250.00
**16**	500.00	500.00	–
**17**	>500.00	>500.00	500.00
**18**	500.00	500.00	125.00
**19**	>500.00	>500.00	500.00
**20**	500.00	500.00	250.00
**21**	500.00	500.00	250.00
**22**	500.00	500.00	125.00
**23**	250.00	500.00	250.00
**24**	500.00	500.00	250.00
**25**	>500.00	>500.00	250.00
**26**	500.00	500.00	250.00
**27**	500.00	500.00	125.00
**28**	500.00	500.00	250.00
**29**	500.00	500.00	250.00
**30**	500.00	500.00	250.00
**31**	500.00	500.00	250.00
**32**	500.00	500.00	250.00
**33**	500.00	500.00	250.00
**34**	500.00	500.00	250.00
**35**	500.00	500.00	250.00
**^a^** **Penicillin**	62.50	62.50	250.00
**^a^** **Tetracycline**	15.62	31.25	125.00

“a” means positive control; “b” means MIC; “–” indicates that the experiment has not been performed.

**Table 4:** BE (kcal/mol) for compounds **1**–**67**.

Compounds	^1^ BE (kcal/mol)	^2^ BE (kcal/mol)	Compounds	^1^ BE (kcal/mol)	^2^ BE (kcal/mol)
**1**	−6.73	−6.94	**35**	−6.28	−6.22
**2**	−7.18	−7.67	**36**	−7.21	−7.77
**3**	-	−4.24	**37**	−6.96	−6.69
**4**	-	−4.37	**38**	−6.69	−7.39
**5**	−5.13	−4.81	**39**	−6.33	−6.56
**6**	-	−4.24	**40**	−6.91	−7.14
**7**	−4.03	−4.62	**41**	−7.23	−7.36
**8**	−4.91	−3.07	**42**	−5.82	−6.05
**9**	−4.35	−4.82	**43**	−6.58	−6.09
**10**	-	−4.82	**44**	−5.97	−6.18
**11**	-	−5.73	**45**	−5.83	−5.64
**12**	−5.53	−5.89	**46**	−6.15	−6.39
**13**	−5.51	−5.92	**47**	−4.59	−4.94
**14**	−5.21	−5.75	**48**	−4.25	−4.33
**15**	−5.17	−5.52	**49**	−2.85	−3.53
**16**	−5.72	−5.43	**50**	-	−3.89
**17**	−3.13	−3.61	**51**	−3.15	−4.65
**18**	−6.14	−6.39	**52**	−4.61	−4.47
**19**	−4.11	−4.94	**53**	−4.85	−4.69
**20**	−4.63	−4.88	**54**	−5.70	−5.78
**21**	−4.05	−4.65	**55**	−4.84	−4.74
**22**	−4.00	−4.70	**56**	−5.09	−4.99
**23**	-	-	**57**	−5.35	−5.71
**24**	-	−3.97	**58**	−5.36	−5.99
**25**	-	-	**59**	−5.86	−5.73
**26**	−6.51	−6.75	**60**	−5.67	−5.67
**27**	−5.19	−4.10	**61**	-	−5.35
**28**	−4.40	−4.47	**62**	−3.88	−5.12
**29**	-	−4.40	**63**	−6.66	−7.25
**30**	−5.17	−5.21	**64**	−6.67	−6.53
**31**	−5.54	−6.09	**65**	−7.72	−8.36
**32**	−5.71	−5.46	**66**	−7.69	−6.51
**33**	−6.74	−6.42	**67**	−6.51	−6.61
**34**	−6.38	−6.26			
**^A^** **(DXMS and DSEC)**	−6.14 and −5.12				
**^A^** **(Acarbose)**	−7.56				

“A” means positive control; “1” means **8ET0**; “2” means **3A4A**; “-” indicates that the experiments were not performed and no molecular docking results.

## Data Availability

The original contributions presented in the study are included in the article and Supplementary Materials, further inquiries can be directed to the corresponding authors.

## Supplementary Materials

The following supporting information can be downloaded at https://www.mdpi.com/article/10.3390/foods13213435/s1.
