# Supplementary material for: Bioactivity-Guided Isolation of Secondary Metabolites from Camellia fascicularis: Antioxidative Antibacterial Activities and Anti-Inflammatory Hypoglycemic Molecular Docking
Source: Foods. 2024 Oct 28;13(21):3435. doi: 10.3390/foods13213435 (PMC11545720; doi:10.3390/foods13213435)

Bioactivity-Guided Isolation of Secondary Metabolites from *Camellia fascicularis*: Antioxidative, Antibacterial, Activities, and Anti-inflammatory, Hypoglycemic Molecular Docking

Jiandong Tang [1,†], Jingjing Li [1,†], Boxiao Wu [1], Ruonan Li [1], Junrong Tang [1], Huan Kan [1], Ping Zhao [1], Yingjun Zhang [2], Weihua Wang [3,*] and Yun Liu [1,*]

[1], Key Laboratory of Forest Resources Conservation and Utilization in the Southwest Mountains of China Ministry of Education, Southwest Forestry University, Kunming 650224, China; hdyknctjd23@swfu.edu.cn (J.D.T.); kiki0908222@swfu.edu.cn (J.L.); wbx1437@swfu.edu.cn (B.W.); lrn@swfu.edu.cn (R.L.); tjrzy2016@swfu.edu.cn (J.R.T.); kanhuan@swfu.edu.cn (H.K.); hypzhao2023@163.com (P.Z.)

[2], State Key Laboratory of Phytochemistry and Plant Resources in West China, Kunming Institute of Botany, Chinese Academy of Sciences, Kunming, 650224 China; zhangyj@mail.kib.ac.cn (Y.Z.)

[3], Yunnan Key Laboratory of Gastrodia and Fungi Symbiotic Biology, Zhaotong University, Zhaotong 657000, Yunnan China

AUTHOR INFORMATION

Corresponding Authors.

*E-mail: 4016@ztu.edu.cn (W.W.); liuyun@swfu.edu.cn (Y.L.)

Structure of the compounds **19−67** [1–3].

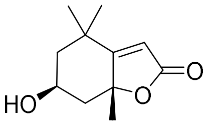

19

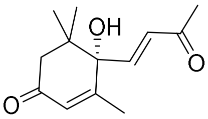

20

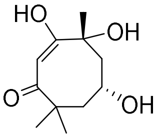

21

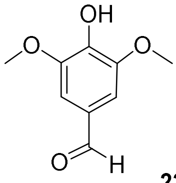

22

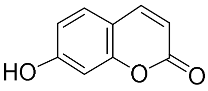

23

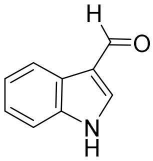

24

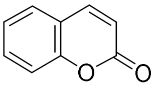

25

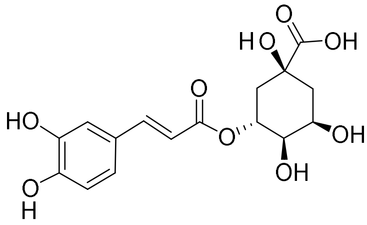

26

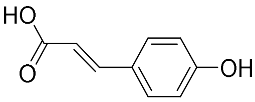

27

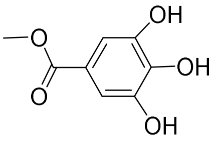

28

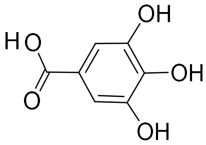

29

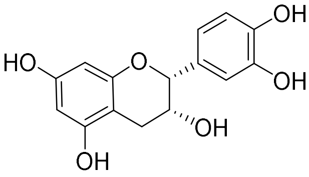

30

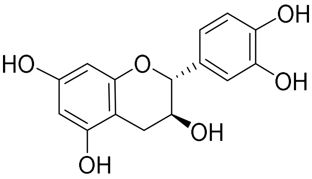

31

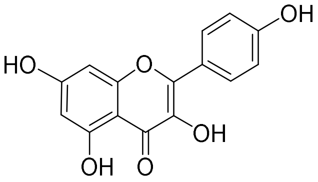

32

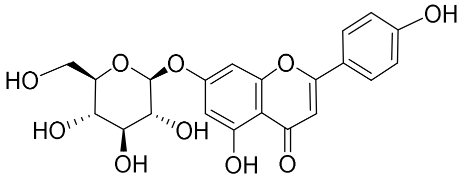

33

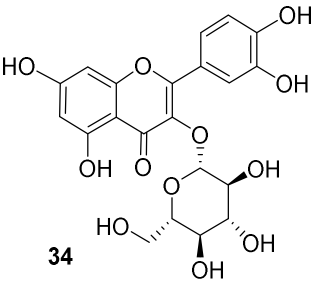

34

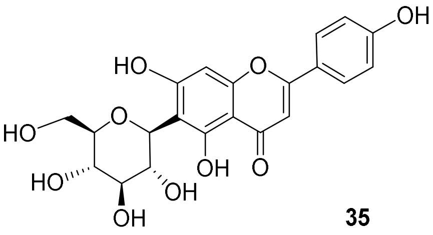

35

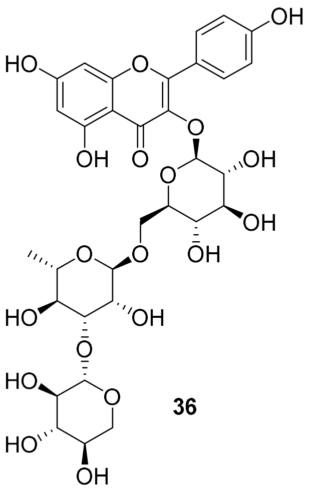

36

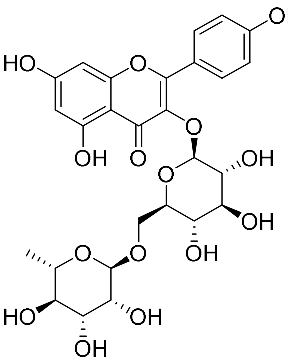

37

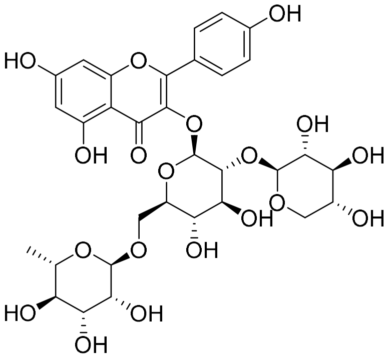

38

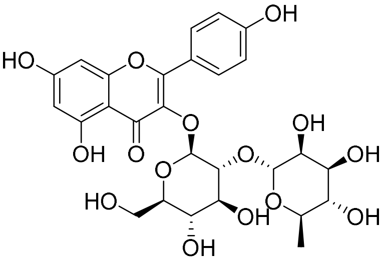

39

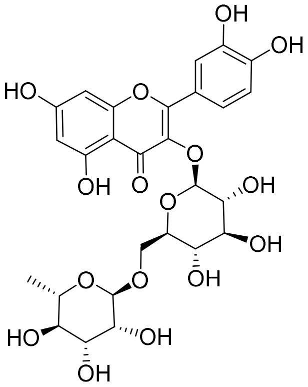

40

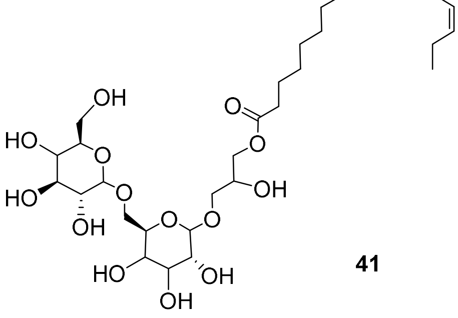

41

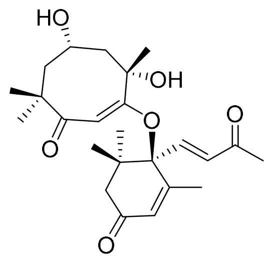

42

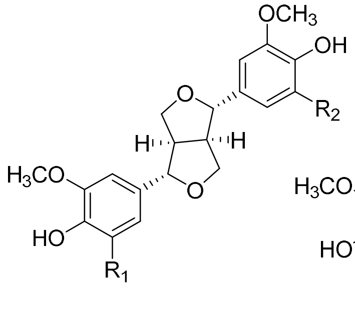

**43**	R_1=OCH_3	R_2=OCH_3
**44**	R_1=H	R_2=OCH_3
**45**	R_1=H	R_2=H

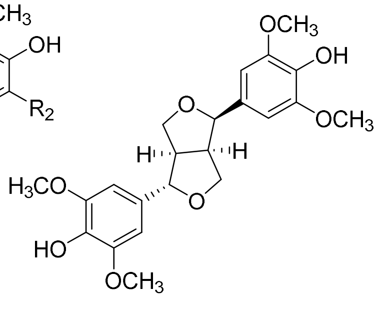

46

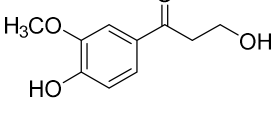

47

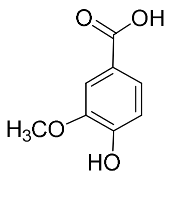

48

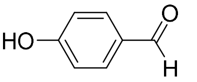

49

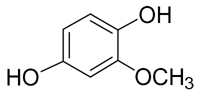

50

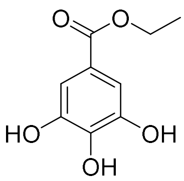

51

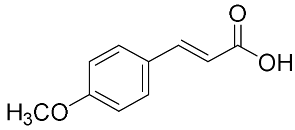

52

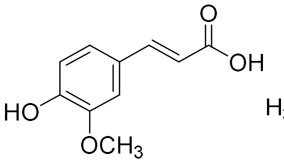

53

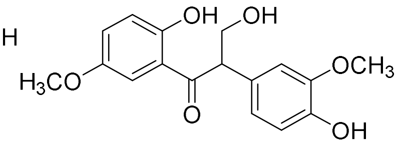

54

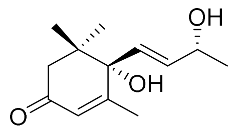

55

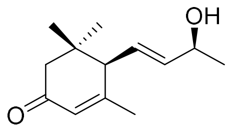

56

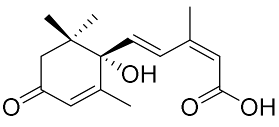

57

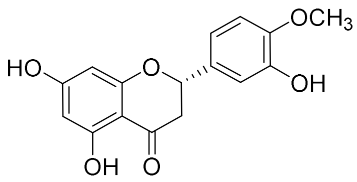

58

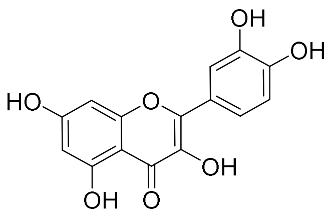

59

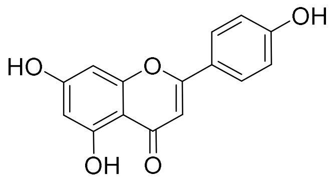

60

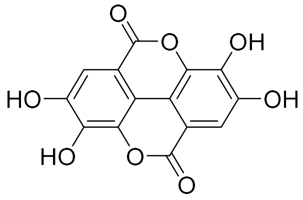

61

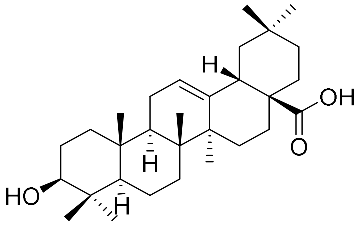

62

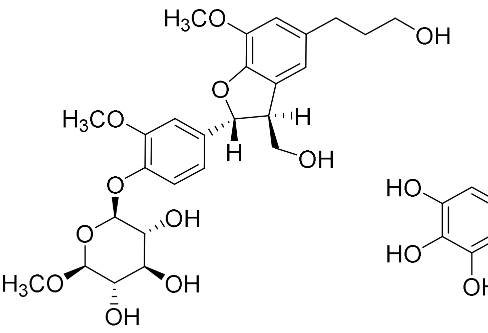

63

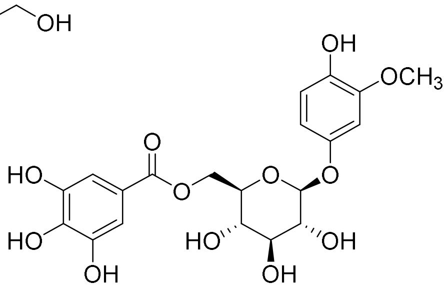

64

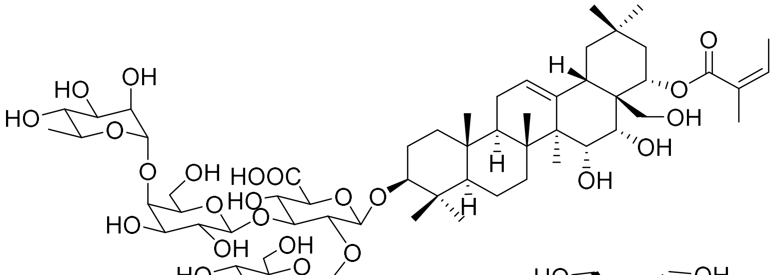

65

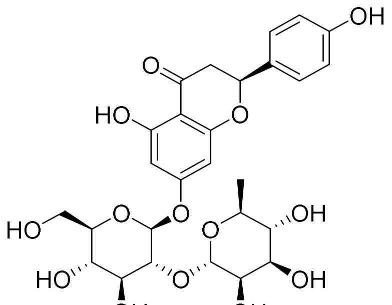

67

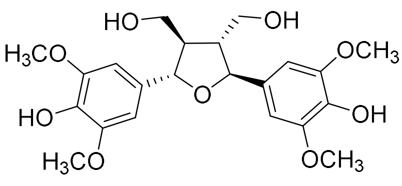

66

References

1. Tang, J.D.; Liu, Y.; Wang, W.H.; Wu, B.X.; Tang, J.R.; Zhang, Y.J.; Zhang, G.L.; Kan, H.; Zhao, P. Chemcical consitiuents from the leaves of *Camellia fascicularis*. *J. Southwest For. Univ. (Natural Sciences)*, **2025**, *45*, 1–6. https://doi.org/10.11929/j.swfu.202401058
2. Tang, J.D.; Li, R.L.; Wu, B.X.; Tang, J.R.; Kan, H.; Zhao, P.; Zhang, Y.J.; Wang, W.H.; Liu, Y. Secondary metabolites with antioxidant and antimicrobial activities from *Camellia fascicularis*. *Curr. Issues Mol. Biol.* **2024**, *46*, 6769–6782. https://doi.org/10.3390/cimb46070404
3. Li, R.L.; Tang, J.D.; Li, J.J.; Wu, B.X.; Tang, J.R.; Kan, H.; Zhao, P.; Zhang, Y.J.; Wang, W.H., Liu, Y. Bioactivity-guided isolation of secondary metabolites with antioxidant and antimicrobial activities from *Camellia fascicularis*. *Foods* **2024**, *13*, 2266. https://doi.org/10.3390/foods13142266

Bioactivity-Guided Isolation of Secondary Metabolites from *Camellia fascicularis*: Antioxidative, Antibacterial, Activities, and Anti-inflammatory, Hypoglycemic Molecular Docking

Jiandong Tang [1,†], Jingjing Li [1,†], Boxiao Wu [1], Ruonan Li [1], Junrong Tang [1], Huan Kan [1], Ping Zhao [1], Yingjun Zhang [2], Weihua Wang [3,*] and Yun Liu [1,*]

[1], Key Laboratory of Forest Resources Conservation and Utilization in the Southwest Mountains of China Ministry of Education, Southwest Forestry University, Kunming 650224, China; hdyknctjd23@swfu.edu.cn (J.D.T.); kiki0908222@swfu.edu.cn (J.L.); wbx1437@swfu.edu.cn (B.W.); lrn@swfu.edu.cn (R.L.); tjrzy2016@swfu.edu.cn (J.R.T.); kanhuan@swfu.edu.cn (H.K.); hypzhao@swfu.edu.cn (P.Z.)

[2], State Key Laboratory of Phytochemistry and Plant Resources in West China, Kunming Institute of Botany, Chinese Academy of Sciences, Kunming, 650224 China; zhangyj@mail.kib.ac.cn (Y.Z.)

[3], Yunnan Key Laboratory of Gastrodia and Fungi Symbiotic Biology, Zhaotong University, Zhaotong 657000, Yunnan China

AUTHOR INFORMATION

Corresponding Authors.

*E-mail: 4016@ztu.edu.cn (W.W.); liuyun@swfu.edu.cn (Y.L.)

Supporting Information

Table of content 1
Figure S1. 1H NMR spectrum of compound 1 in methanol-*d*4.................................1
Figure S2. 13C NMR spectrum of compound 1 in methanol-*d*4................................2
Figure S3. 1H NMR spectrum of compound 2 in methanol-*d*4.................................3
Figure 4S. 13C NMR spectrum of compound 2 in methanol-*d*4................................4
Figure S5. 1H NMR spectrum of compound 3 in methanol-*d*4.................................5
Figure S6. 1H NMR spectrum of compound 3 in methanol-*d*4.................................6
Figure S7. 1H NMR spectrum of compound 4 in Acetone-*d*6....................................7
Figure S8. 13C NMR spectrum of compound 4 in acetone-*d*6....................................8
Figure S9. 1H NMR spectrum of compound 5 in methanol-*d*4.................................9
Figure S10. 1H NMR spectrum of compound 5 in methanol-*d*4..............................10
Figure S11. 1H NMR spectrum of compound 6 in methanol-*d*4..............................11
Figure S12. 13C NMR spectrum of compound 6 in methanol-*d*4.............................12
Figure S13. 1H NMR spectrum of compound 7 in methanol-*d*4..............................13
Figure S14. 13C NMR spectrum of compound 7 in methanol-*d*4.............................14
Figure S15. 1H NMR spectrum of compound 8 in methanol-*d*4..............................15
Figure S17. 1H NMR spectrum of compound 9 in methanol-*d*4..............................17
Figure S19. 1H NMR spectrum of compound 10 in methanol-*d*4............................19
Figure S20. 13C NMR spectrum of compound 10 in methanol-*d*4...........................20
Figure S21. 1H NMR spectrum of compound 11 in methanol-*d*4............................21
Figure S22. 13C NMR spectrum of compound 11 in methanol-*d*4...........................22
Figure S23. 1H NMR spectrum of compound 12 in methanol-*d*4............................23
Figure S24. 13C NMR spectrum of compound 12 in methanol-*d*4...........................24
Figure S25. 1H NMR spectrum of compound 13 in methanol-*d*4............................25
Figure S26. 13C NMR spectrum of compound 13 in methanol-*d*4...........................26

Figure S27. ^{1}**H NMR spectrum of compound 14 in methanol-*d*4.**............................27

Figure S28. ^{13}**C NMR spectrum of compound 14 in methanol-*d*4.**...........................28

Figure S29. ^{1}**H NMR spectrum of compound 15 in methanol-*d*4.**............................29

Figure S30. ^{13}**C NMR spectrum of compound 15 in methanol-*d*4.**...........................30

Figure S31. ^{1}**H NMR spectrum of compound 16 in methanol-*d*4.**............................31

Figure S32. ^{13}**C NMR spectrum of compound 16 in methanol-*d*4.**...........................32

Figure S33. ^{1}**H NMR spectrum of compound 17 in methanol-*d*4.**............................33

Figure S34. ^{13}**C NMR spectrum of compound 17 in methanol-*d*4.**...........................34

Figure S35. ^{1}**H NMR spectrum of compound 18 in methanol-*d*4.**............................35

Figure S36. ^{13}**C NMR spectrum of compound 18 in methanol-*d*4.**...........................36

Figure S1. ^{1}H NMR spectrum of compound 1 in methanol-d_4

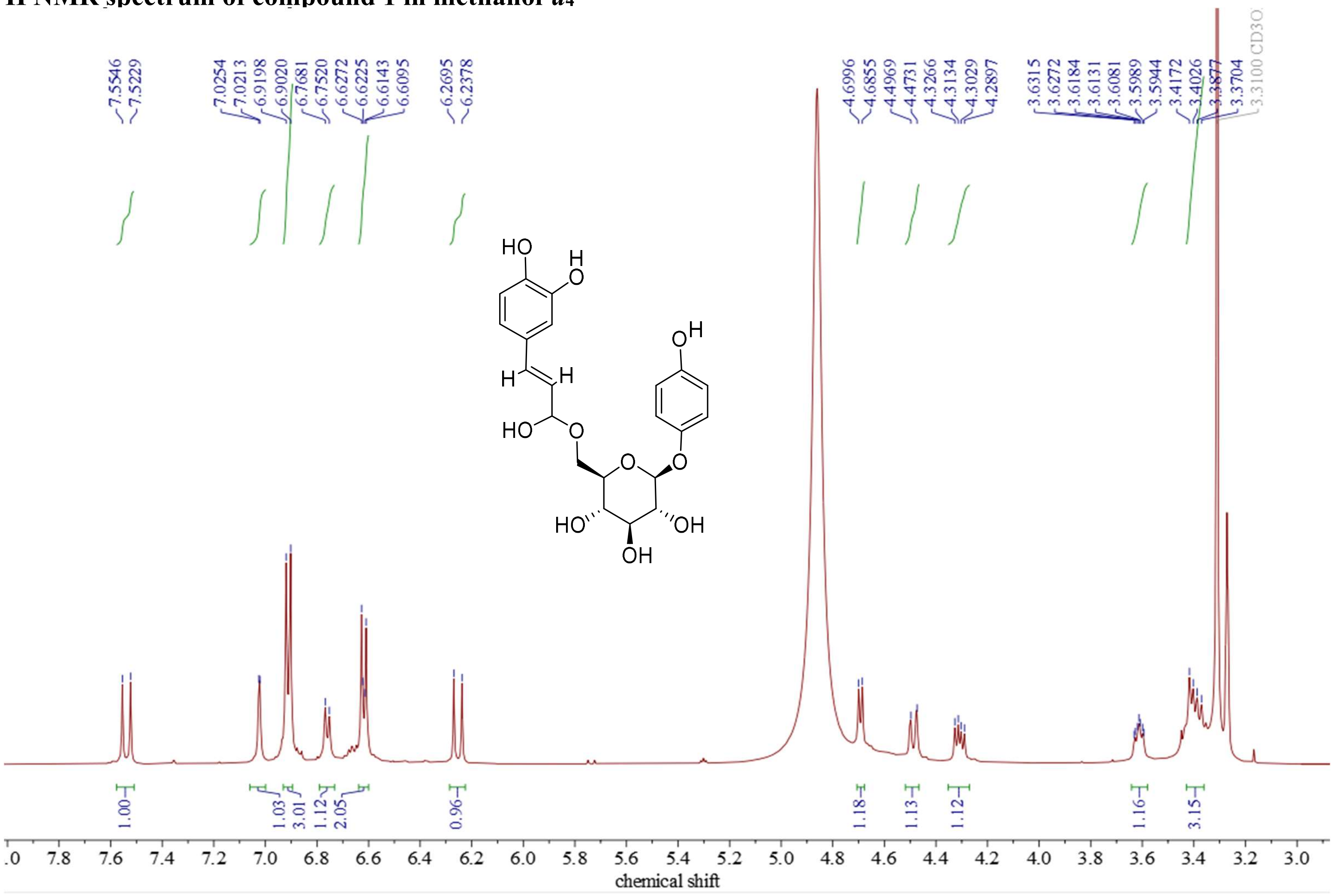

Figure S2. ^{13}C NMR spectrum of compound 1 in methanol-d_4

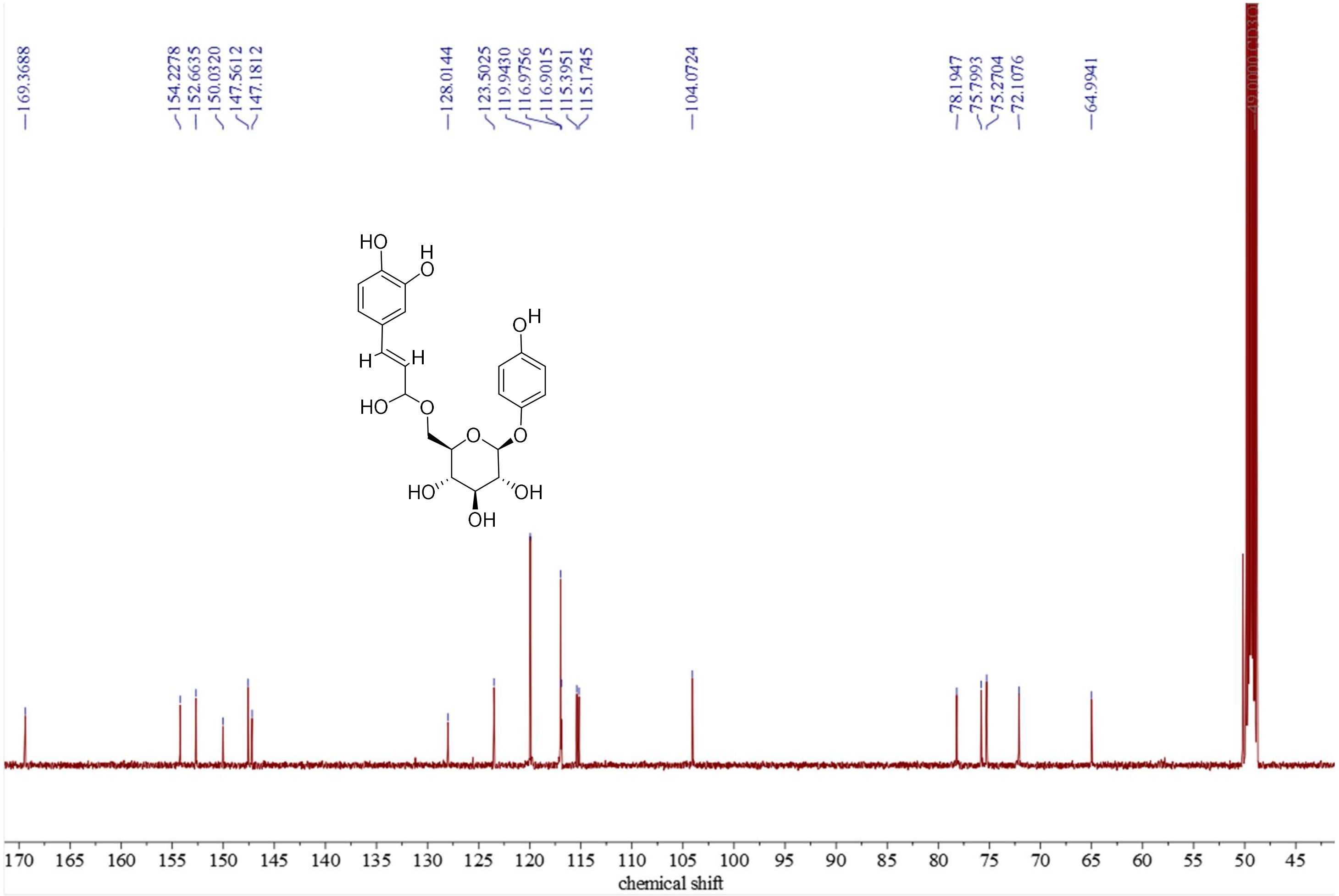

Figure S3. ^{1}H NMR spectrum of compound 2 in methanol-d_4

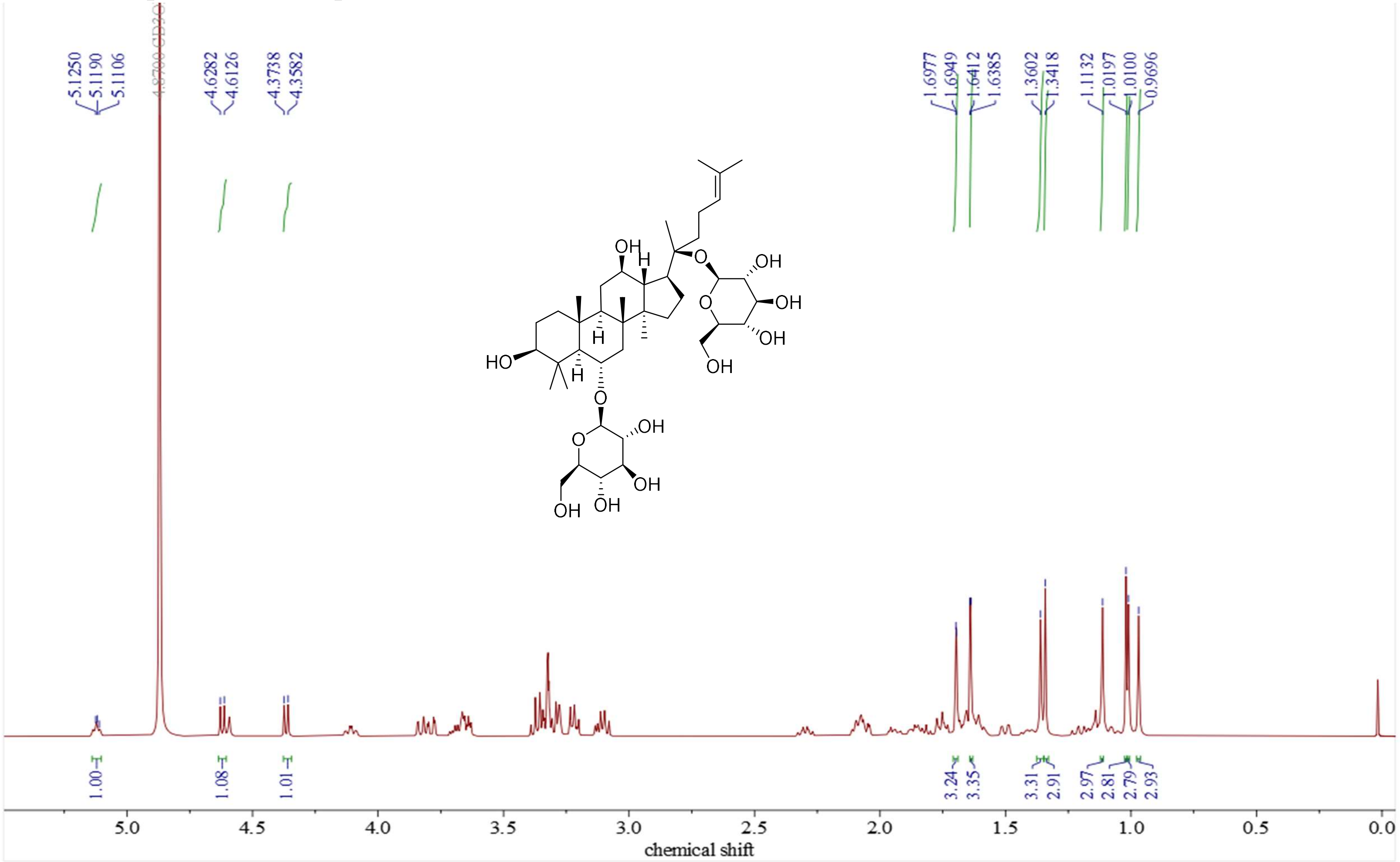

Figure S4. ^{13}C **NMR spectrum of compound 2 in methanol-*d*$_4$**

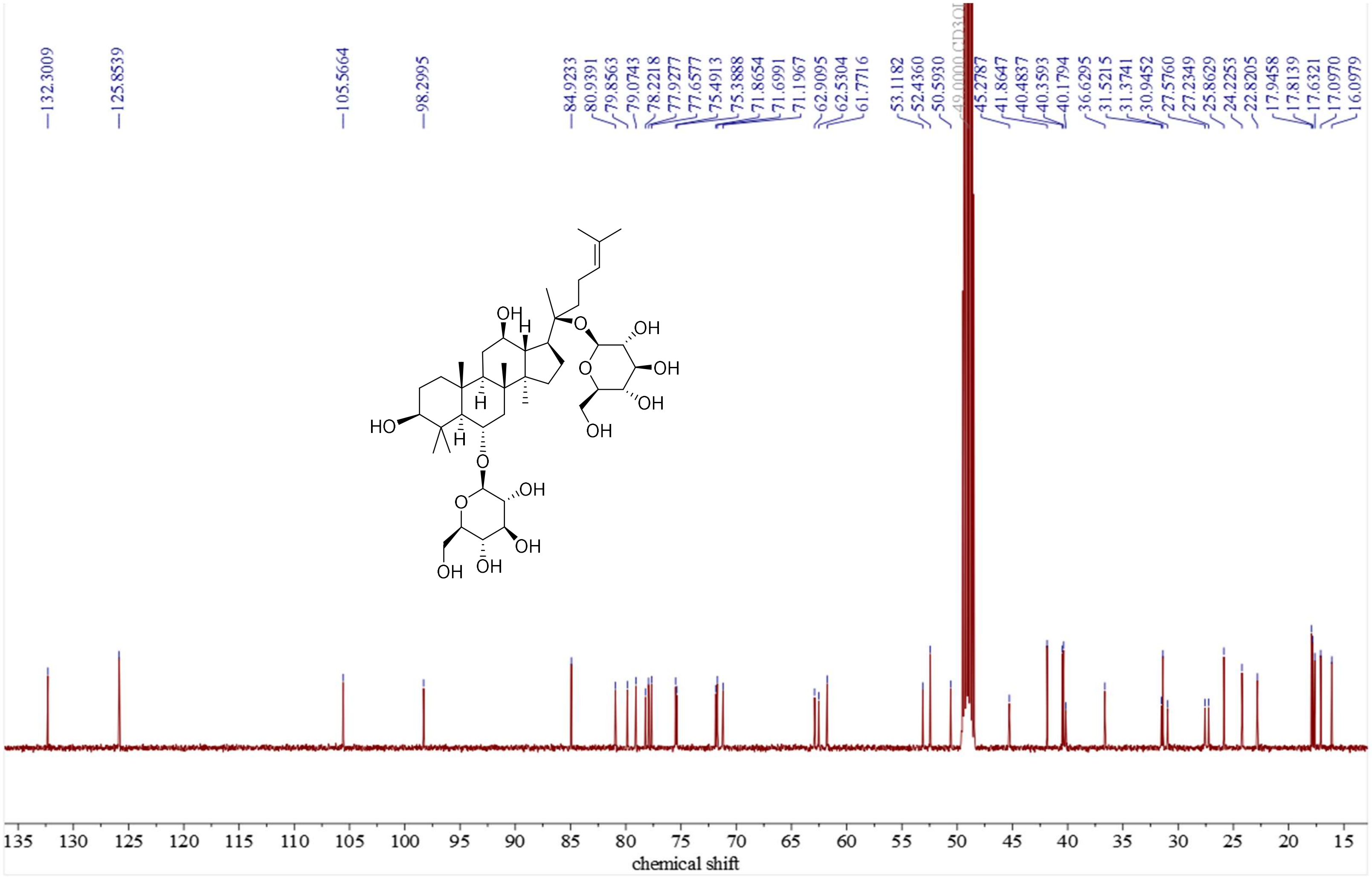

Figure S5. ^{1}H NMR spectrum of compound 3 in methanol-*d*$_4$

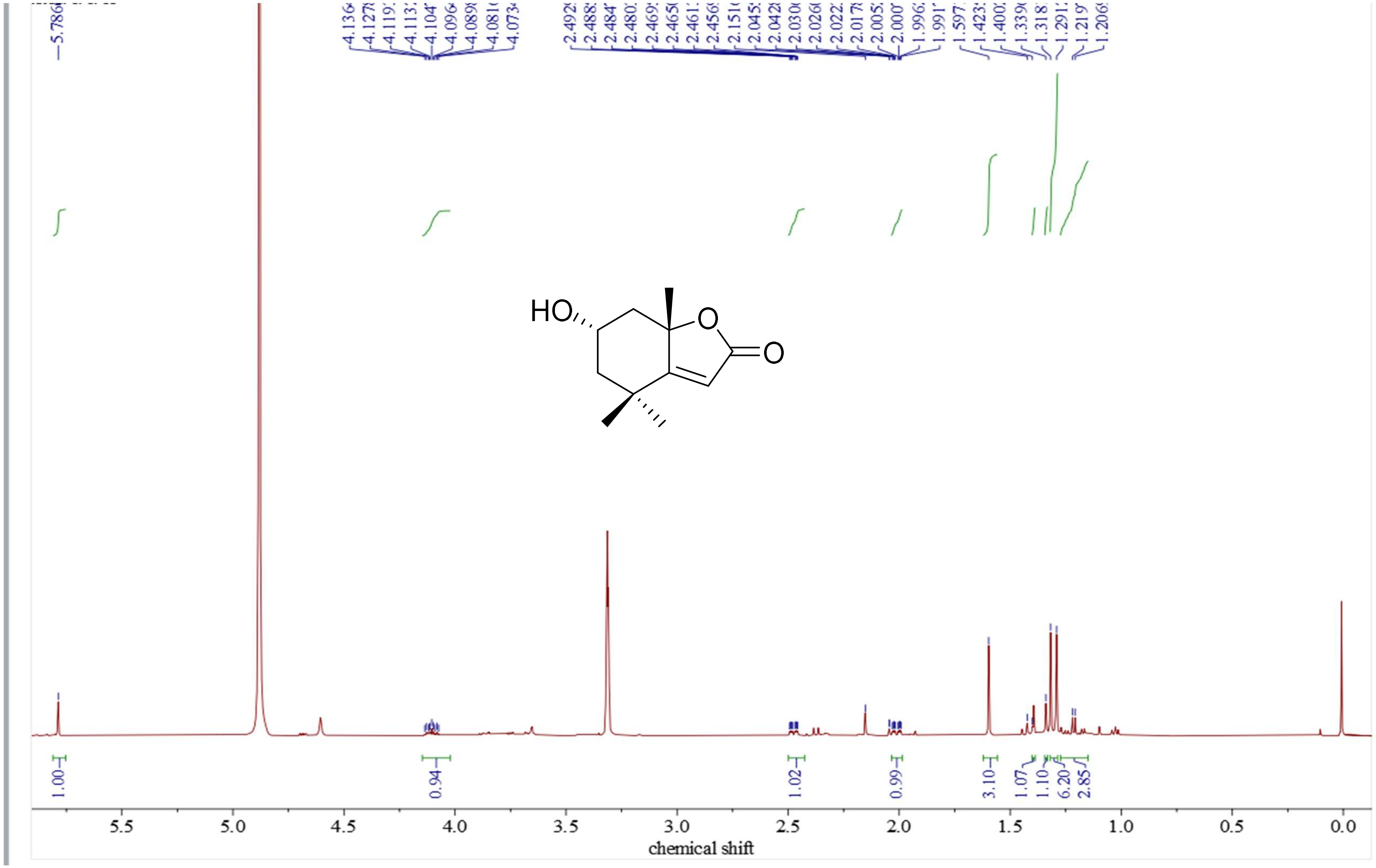

Figure S6. ^{1}H NMR spectrum of compound 3 in methanol-d_4

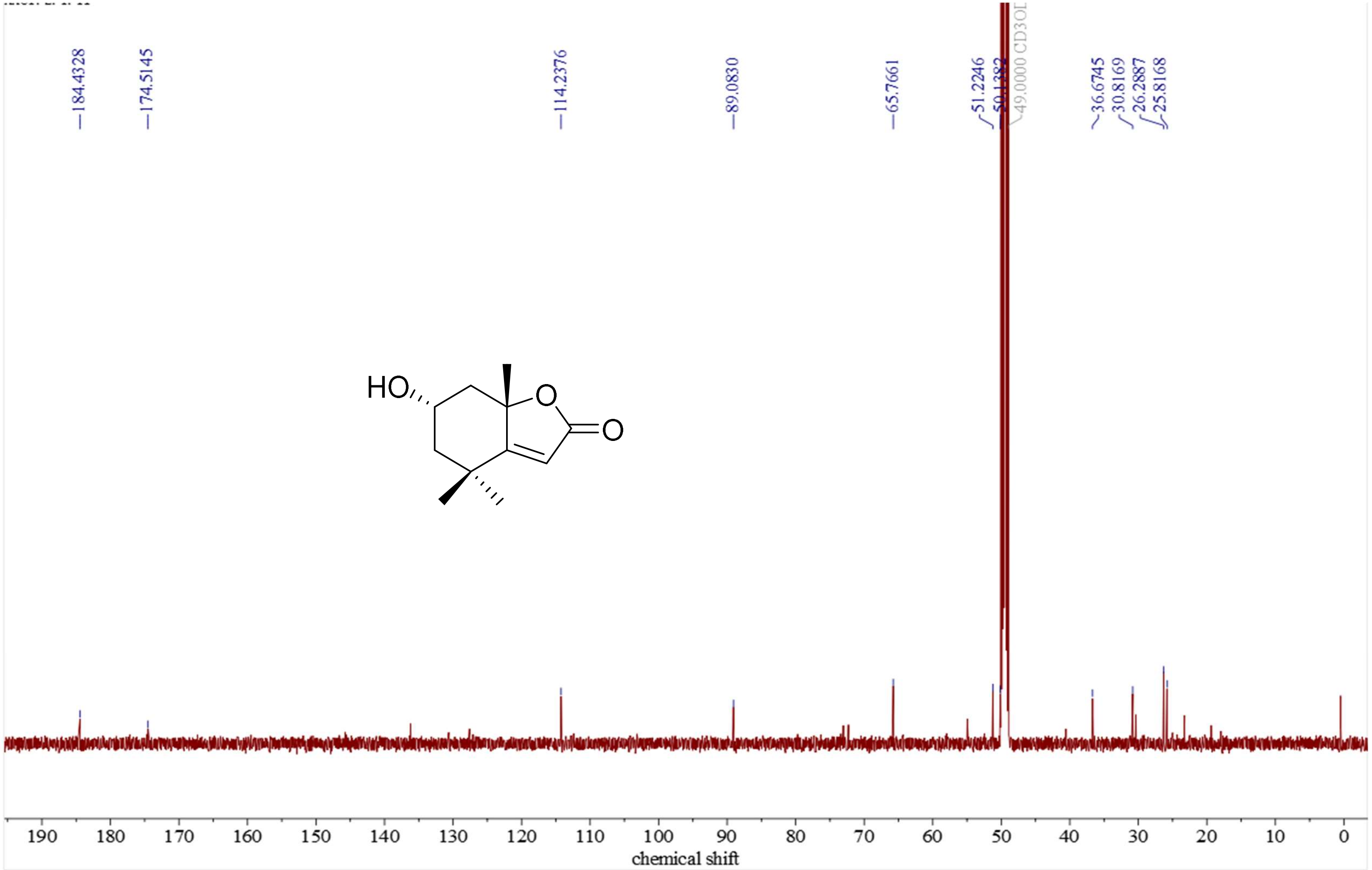

Figure S7. ^{1}H NMR spectrum of compound 4 in methanol-*d*4

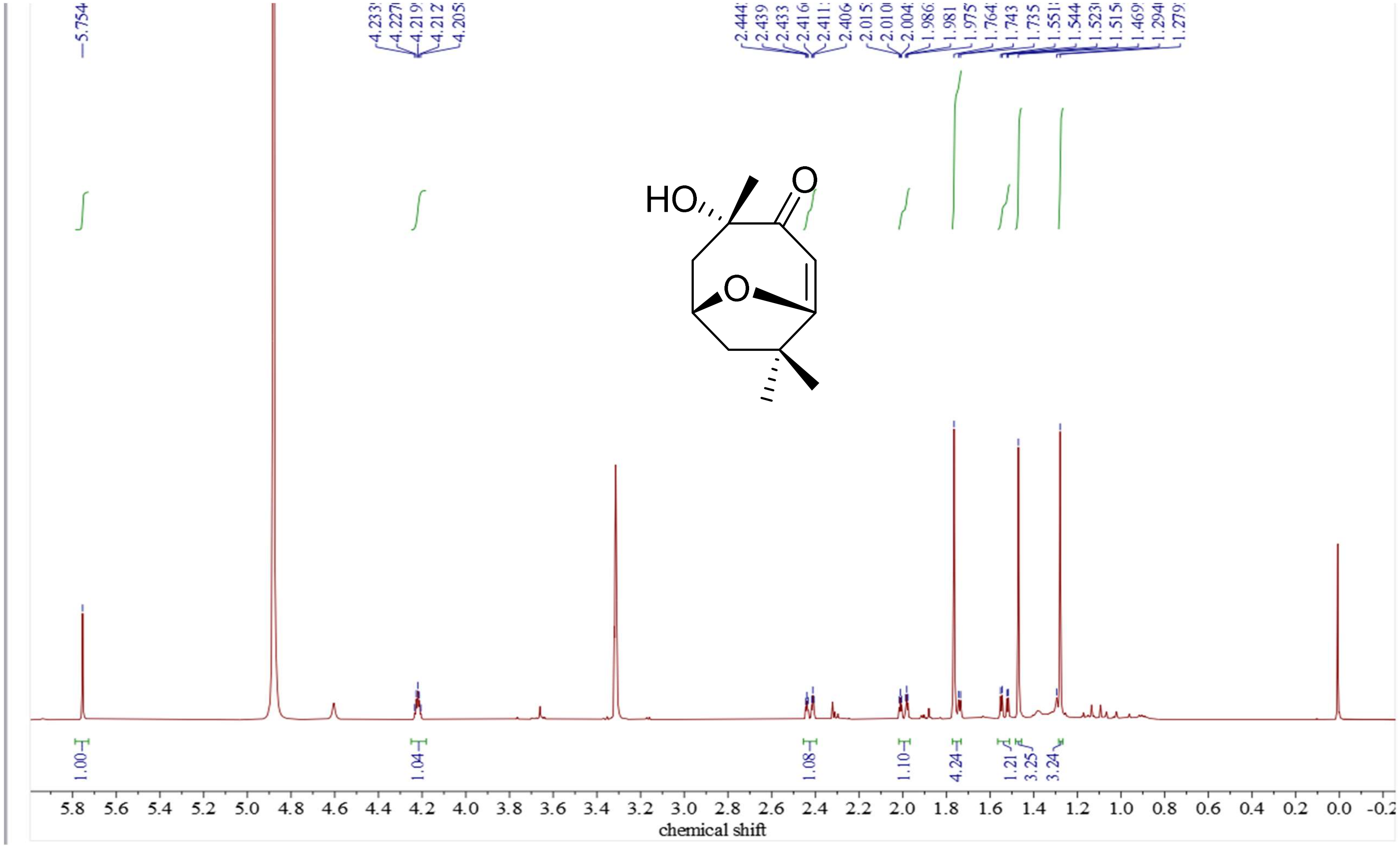

Figure S8. ^{13}C **NMR spectrum of compound 4 in methanol-*d*$_4$**。

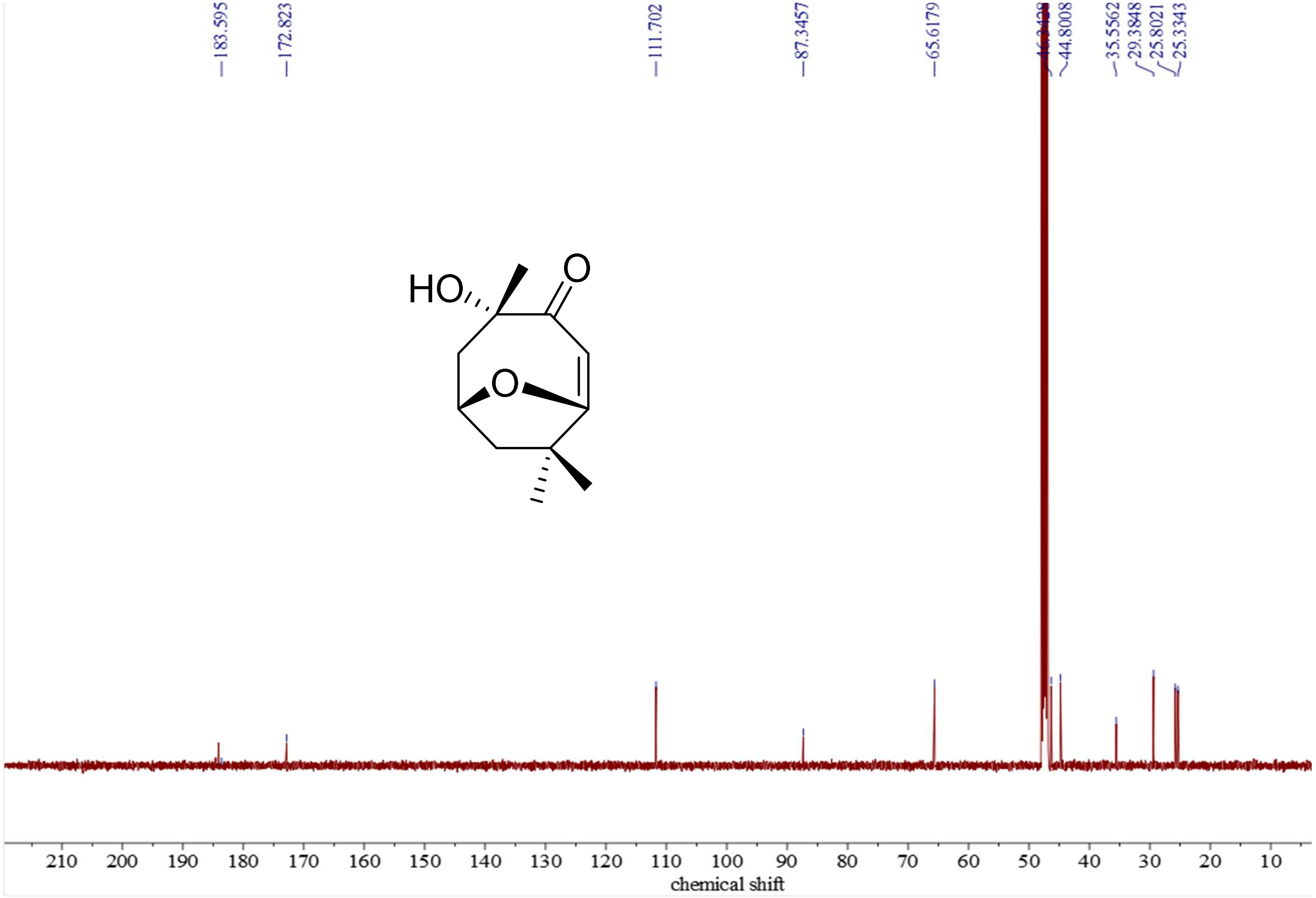

Figure S9. [1]**H NMR spectrum of compound 5 in methanol-*d*4.**

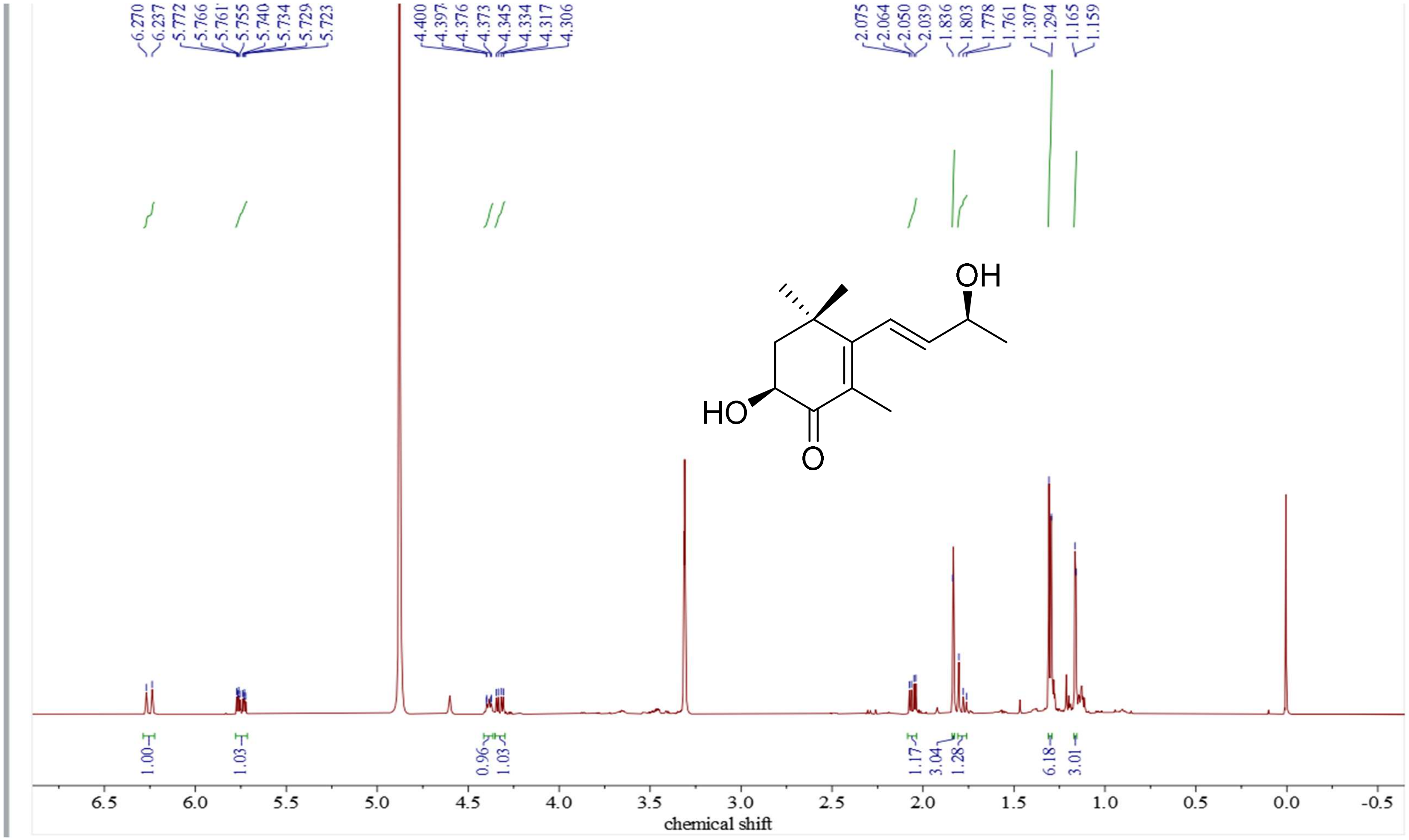

Figure S10. **^{1}H NMR spectrum of compound 5 in methanol-*d_4*.**

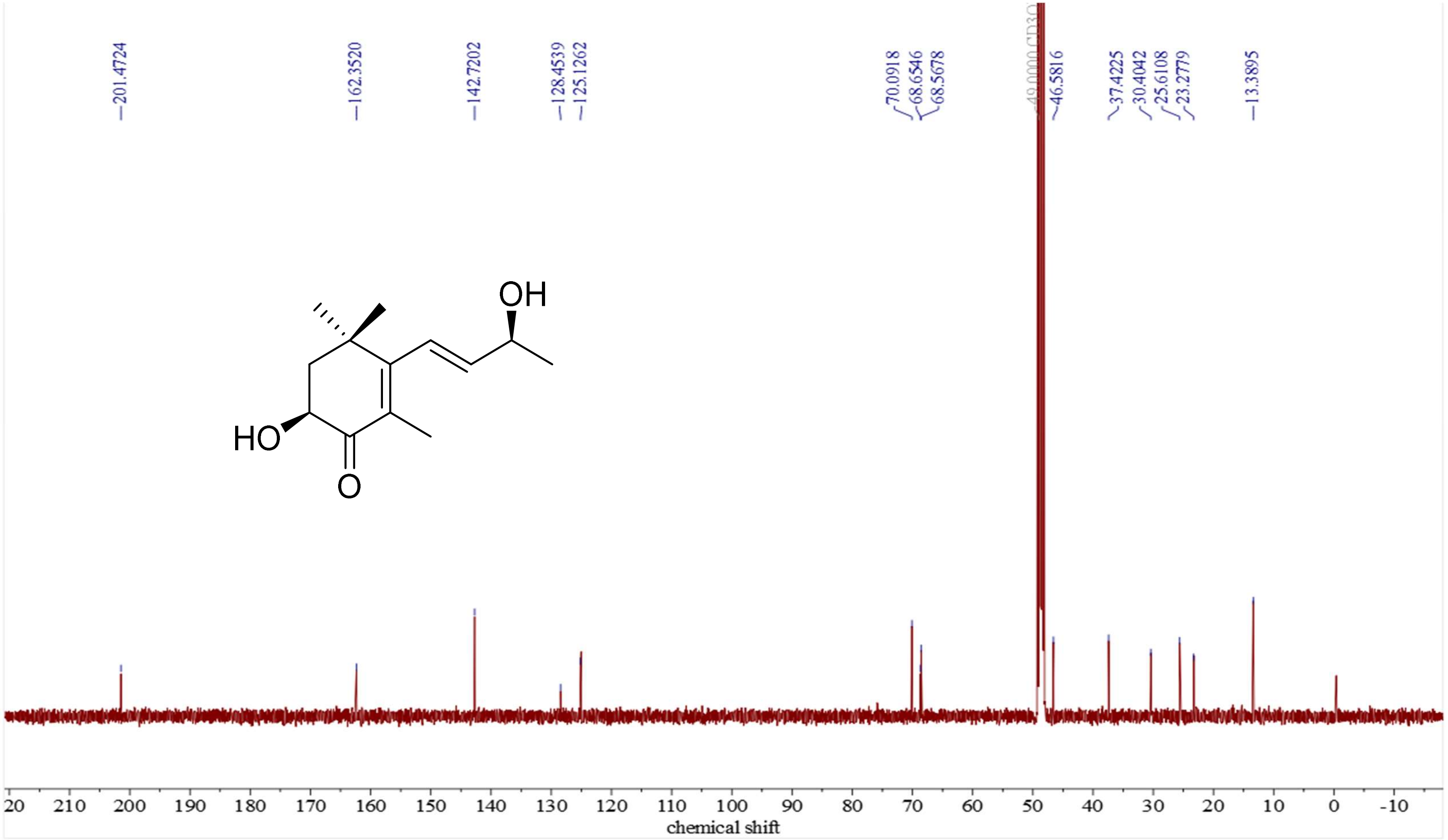

Figure S11. [1]**H NMR spectrum of compound 6 in methanol-*d*4.**

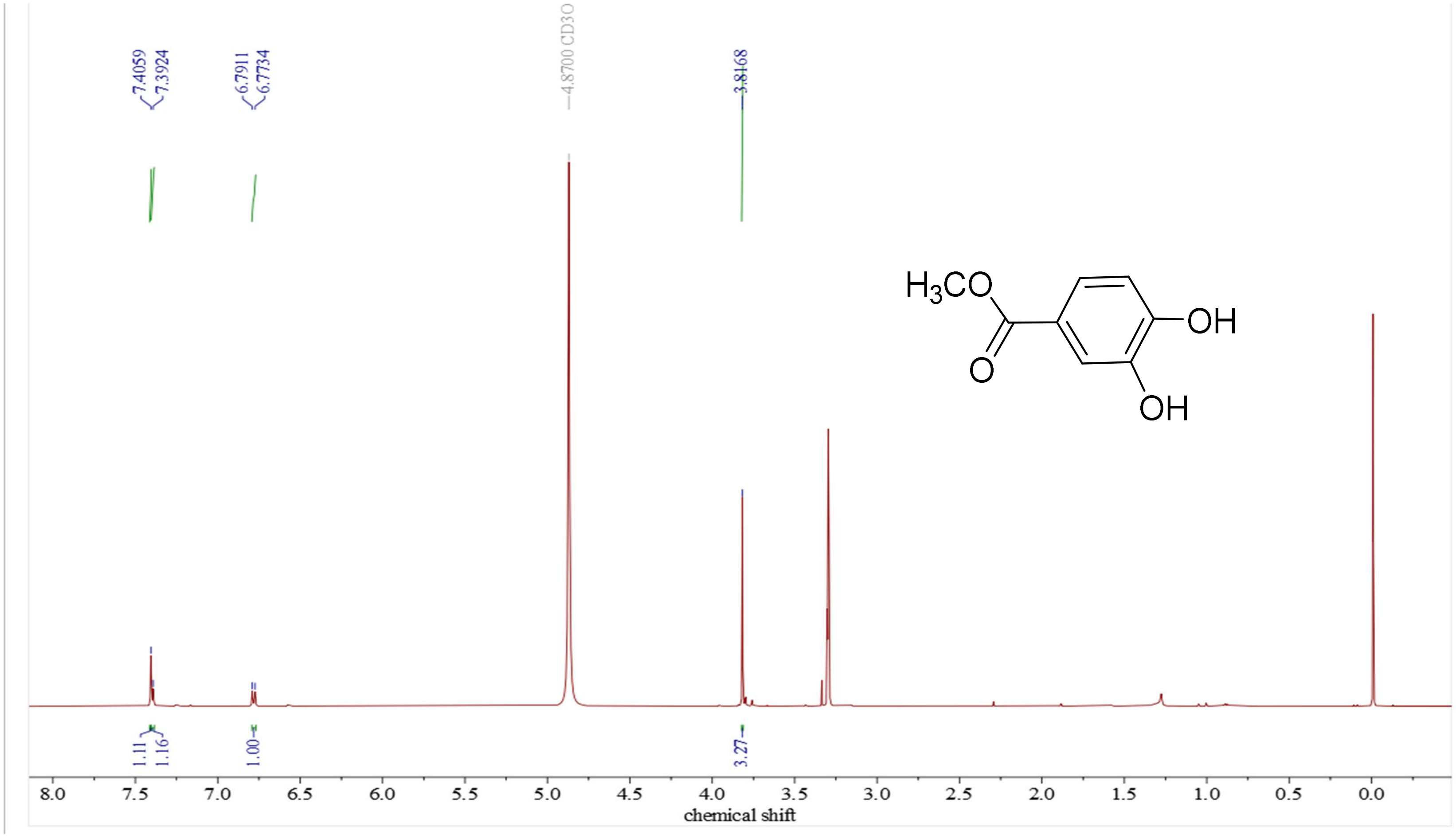

Figure S12. ^{13}C NMR spectrum of compound 6 in methanol-*d*$_4$.

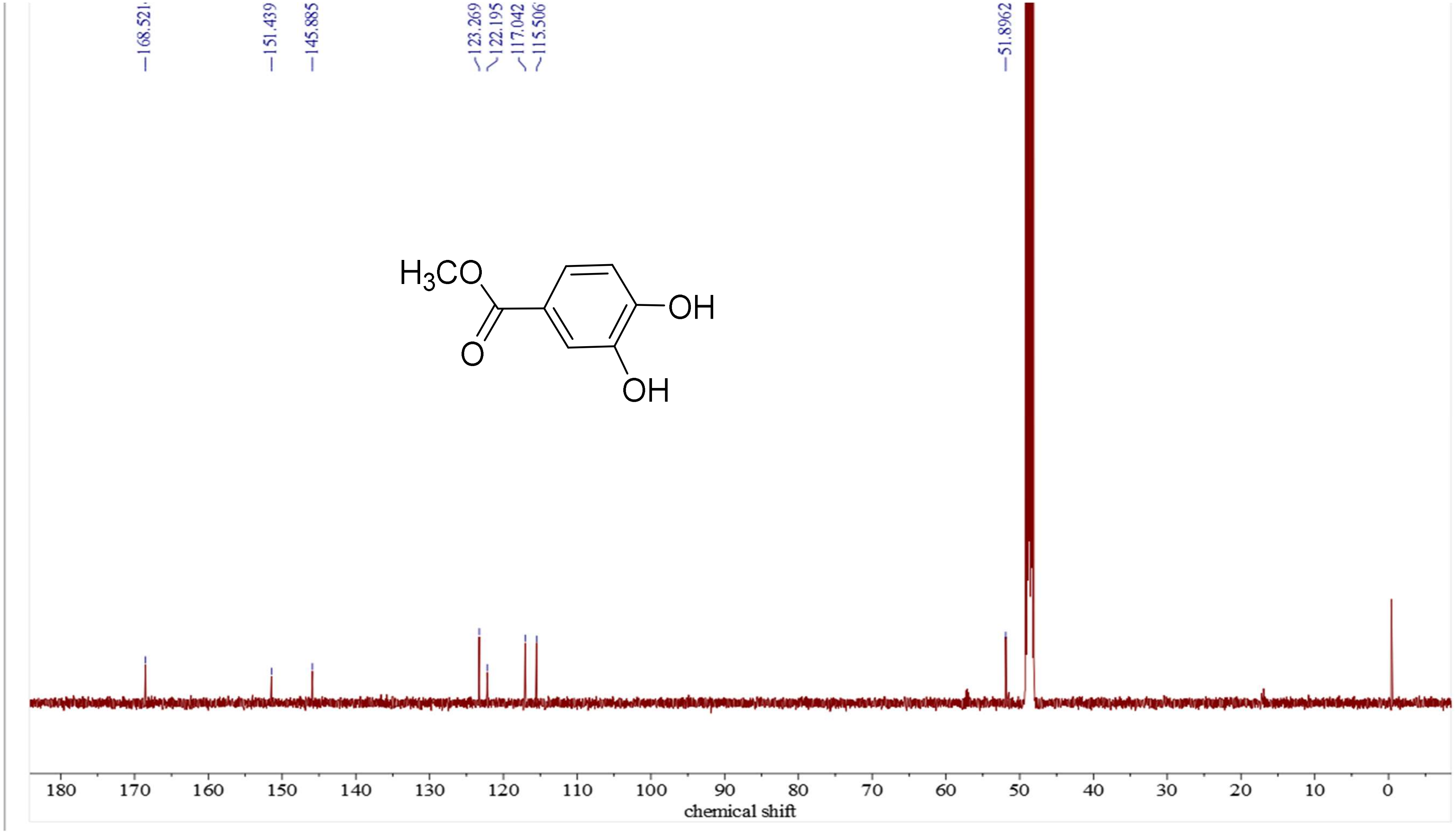

Figure S13. ^{1}H NMR spectrum of compound 7 in methanol-*d*$_4$.

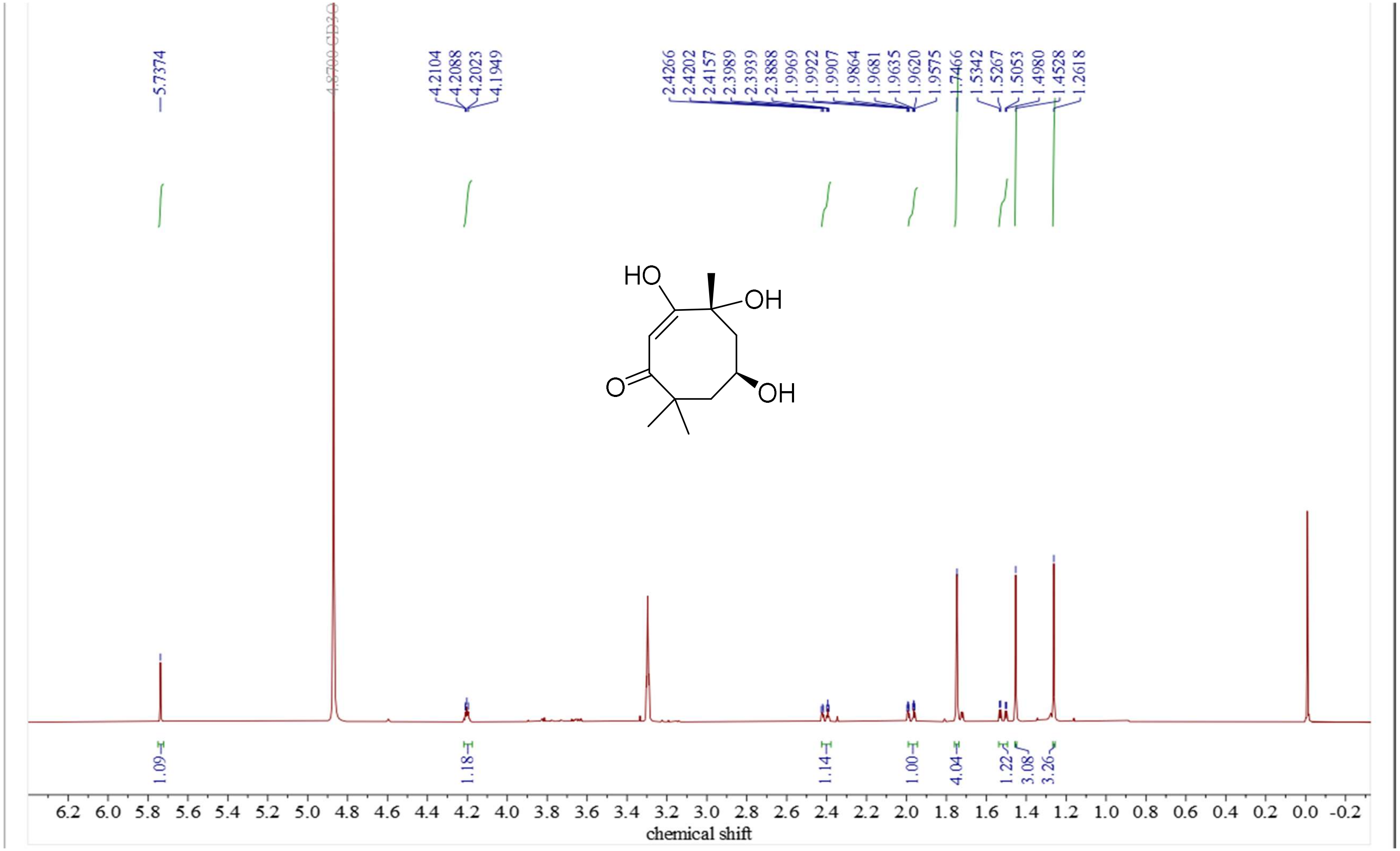

Figure S14. ^{13}**C NMR spectrum of compound 7 in methanol-*d*$_4$.**

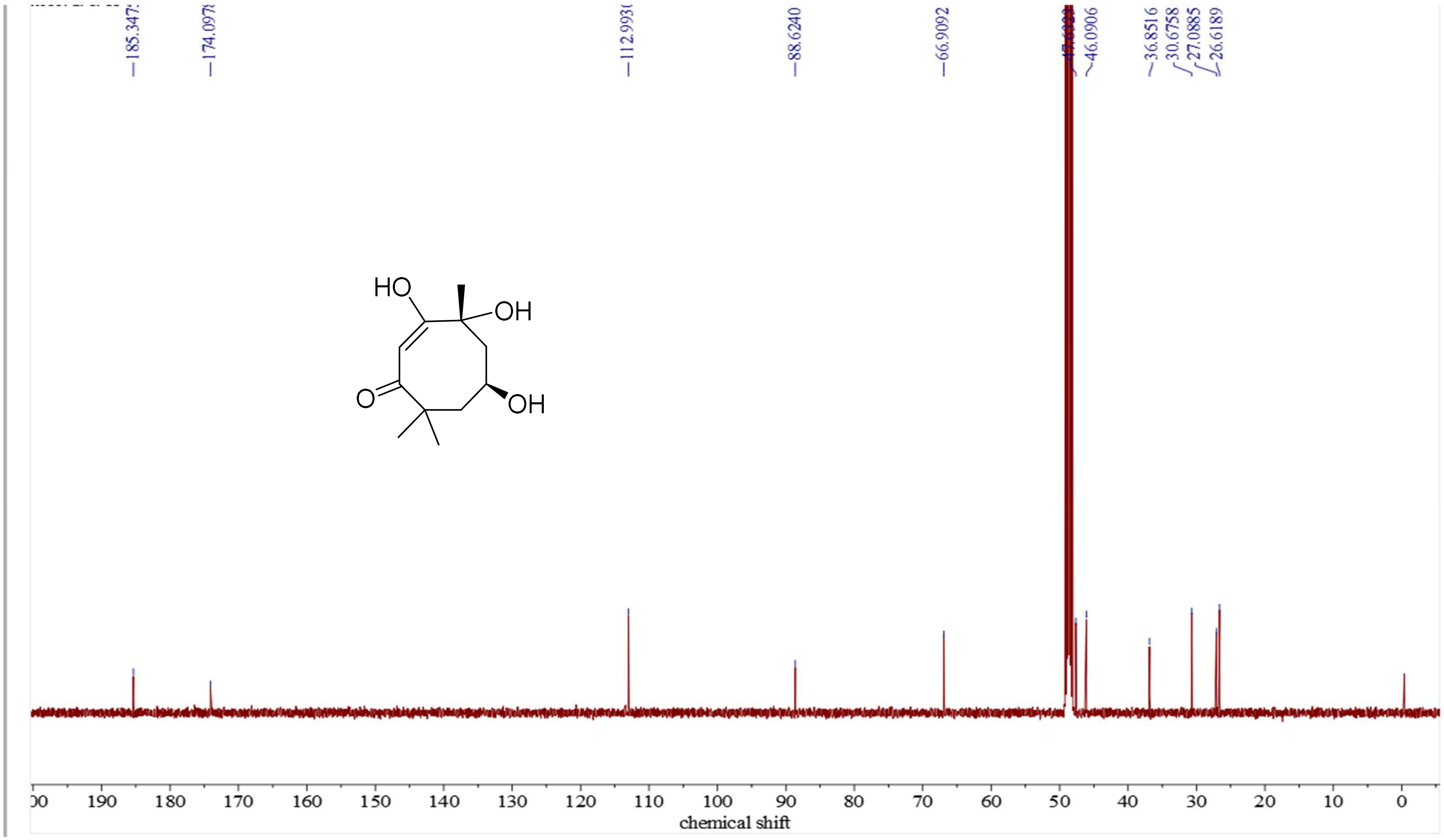

Figure S15. [1]**H NMR spectrum of compound 8 in methanol-*d*4.**

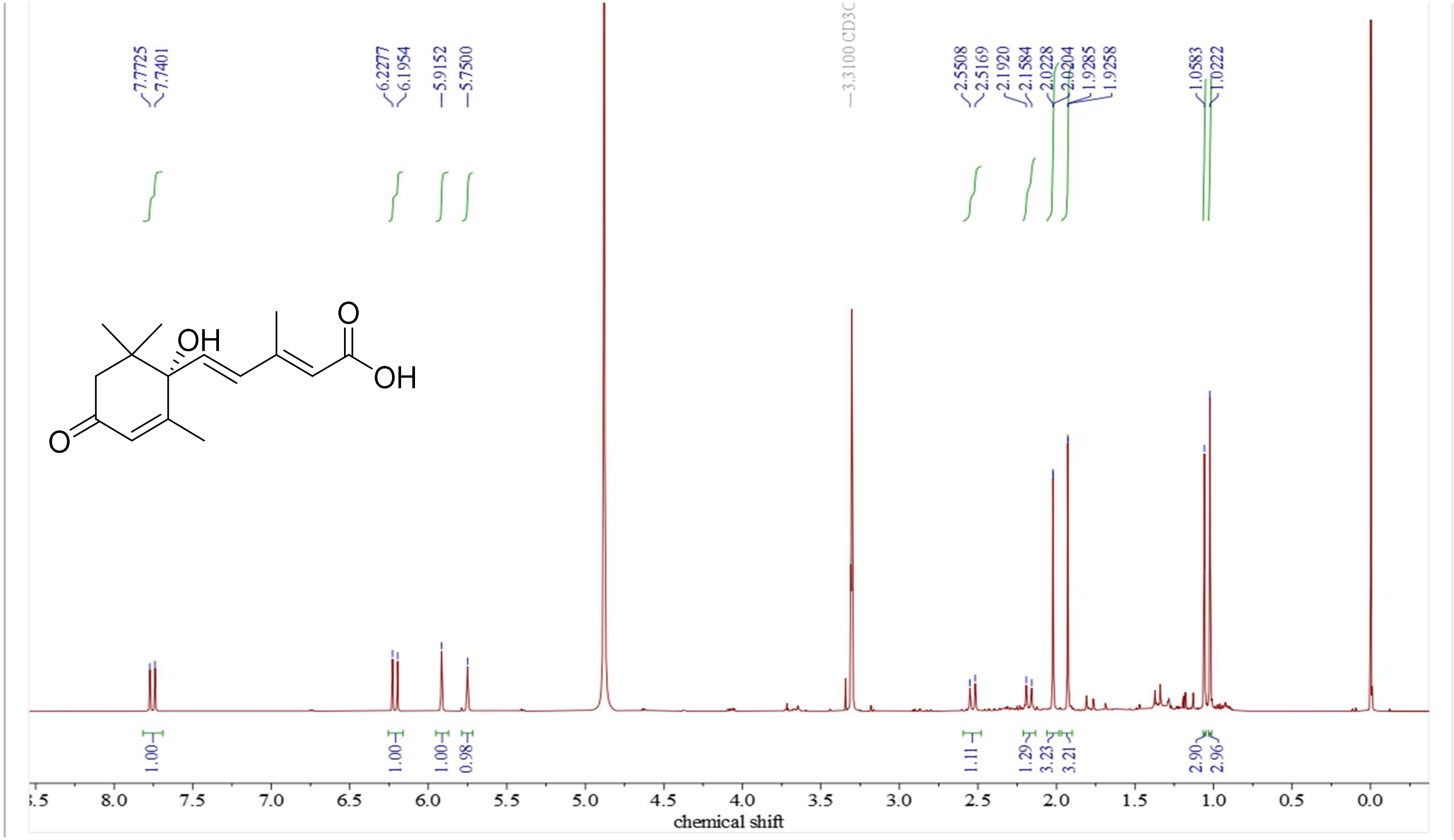

Figure S16. ^{13}C NMR spectrum of compound 8 in methanol-d_4.

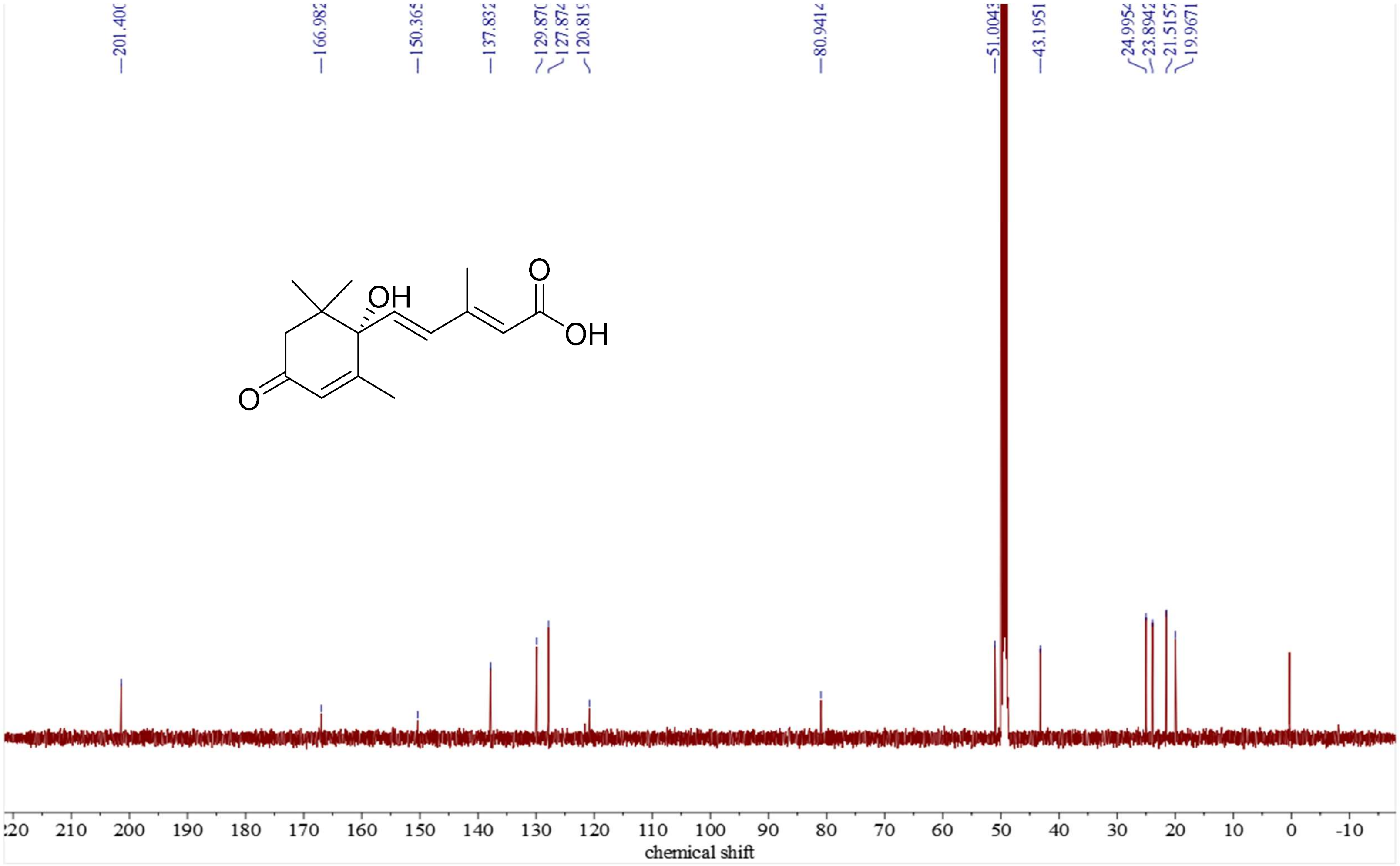

Figure S17. ^{1}H NMR spectrum of compound 9 in methanol-*d*$_4$.

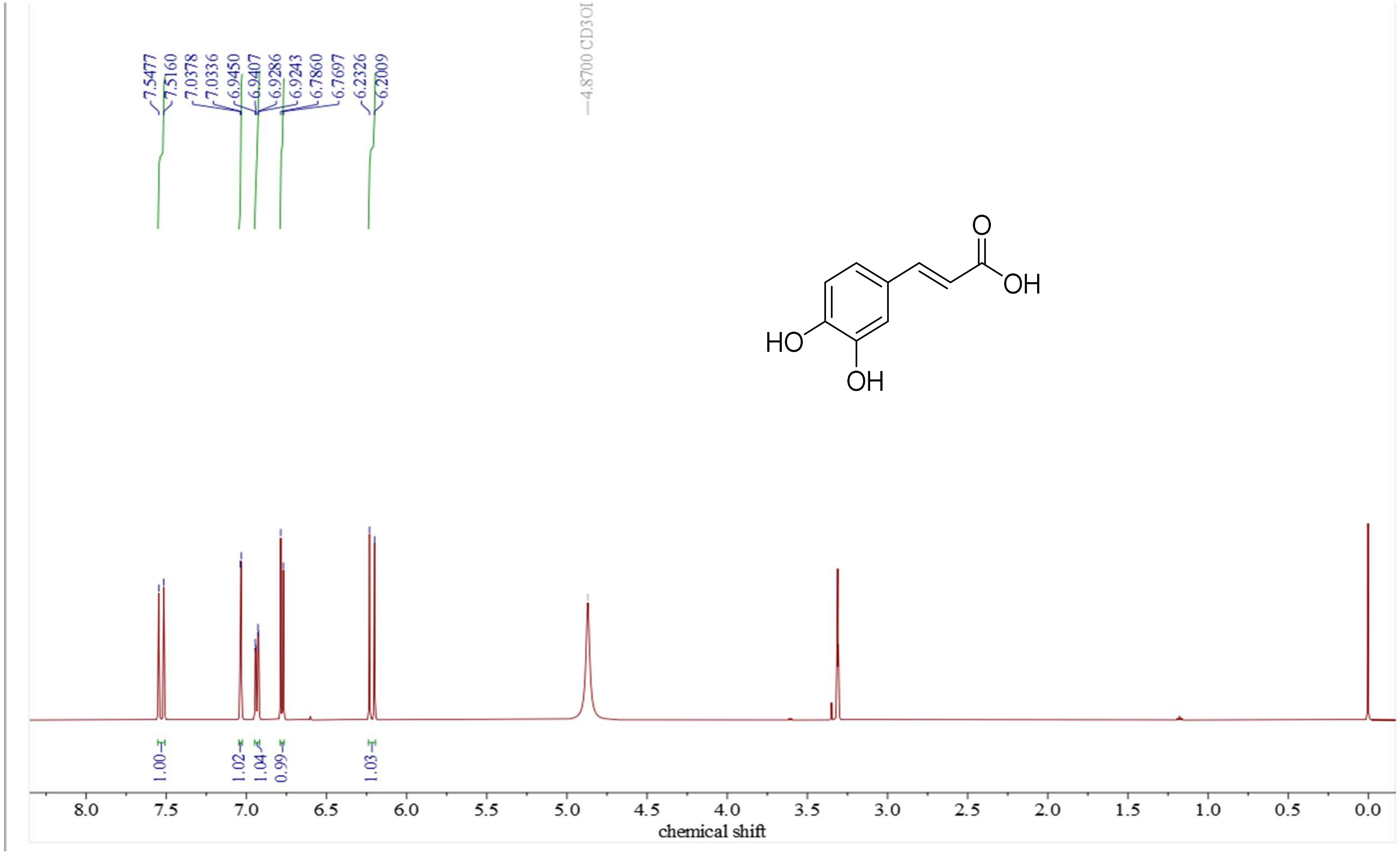

Figure S18. ^{13}C NMR spectrum of compound 9 in methanol-d_4.

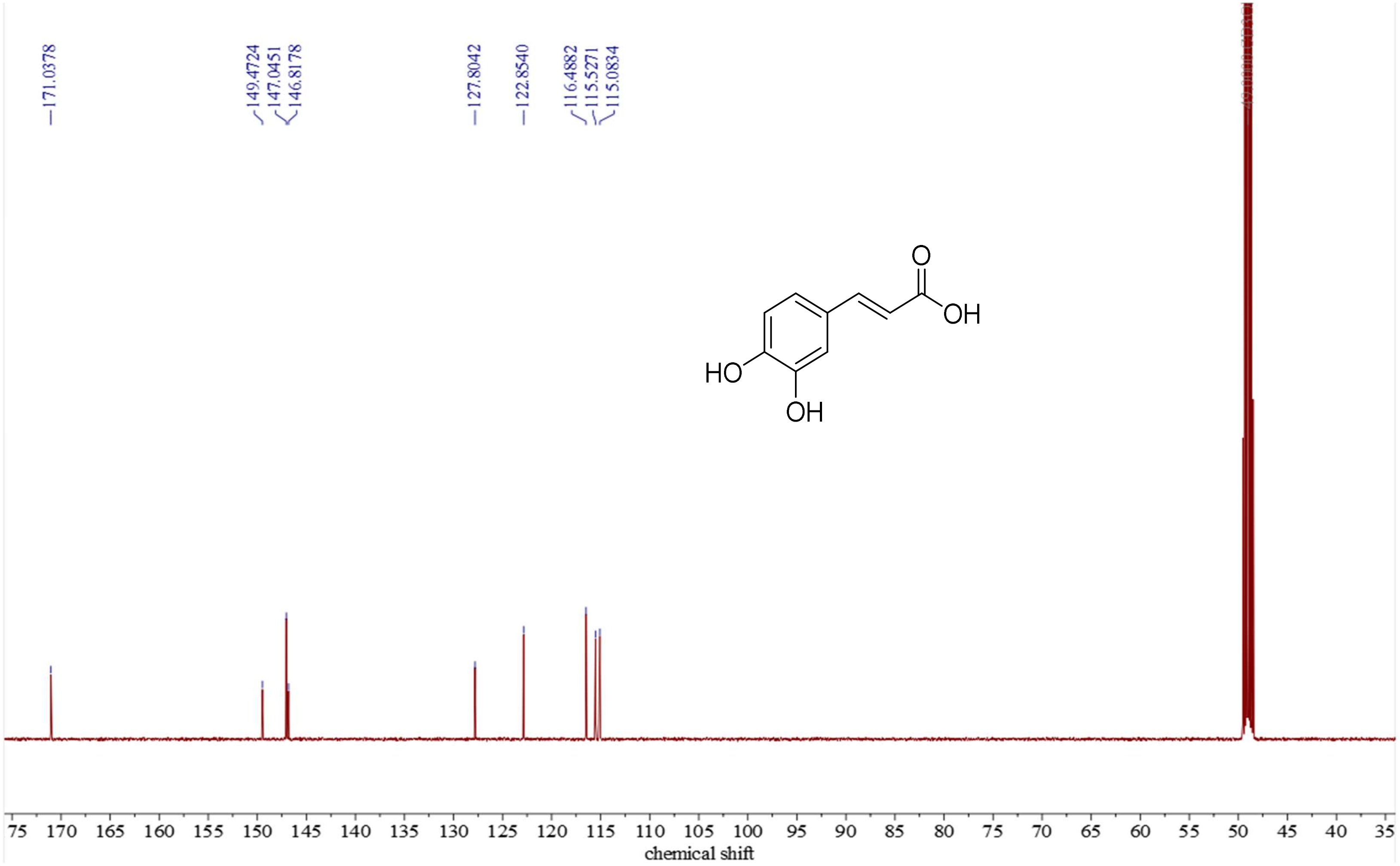

Figure S19. ^{1}H NMR spectrum of compound 10 in methanol-d_4.

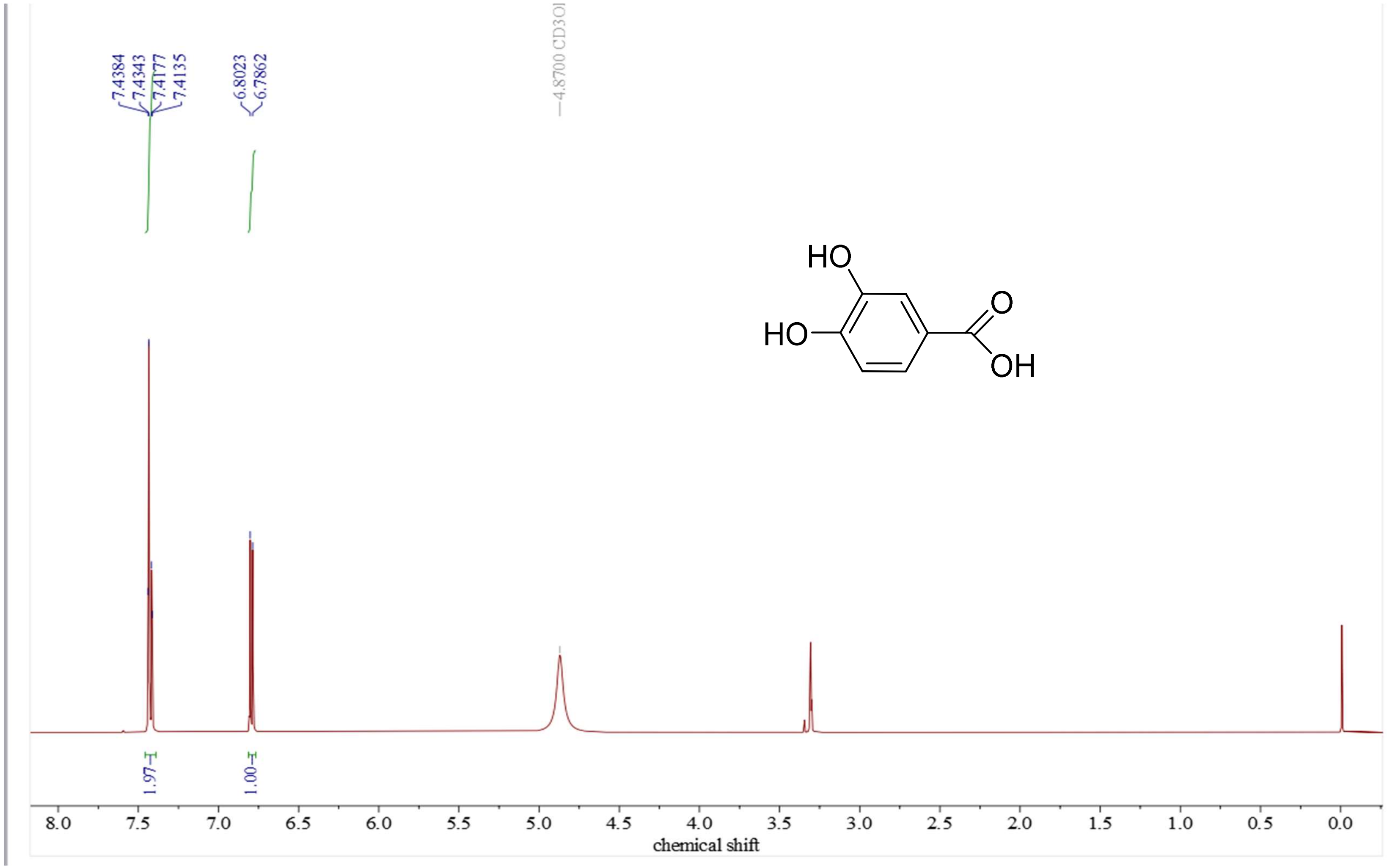

Figure S20. ^{13}**C NMR spectrum of compound 10 in methanol-*d*$_4$.**

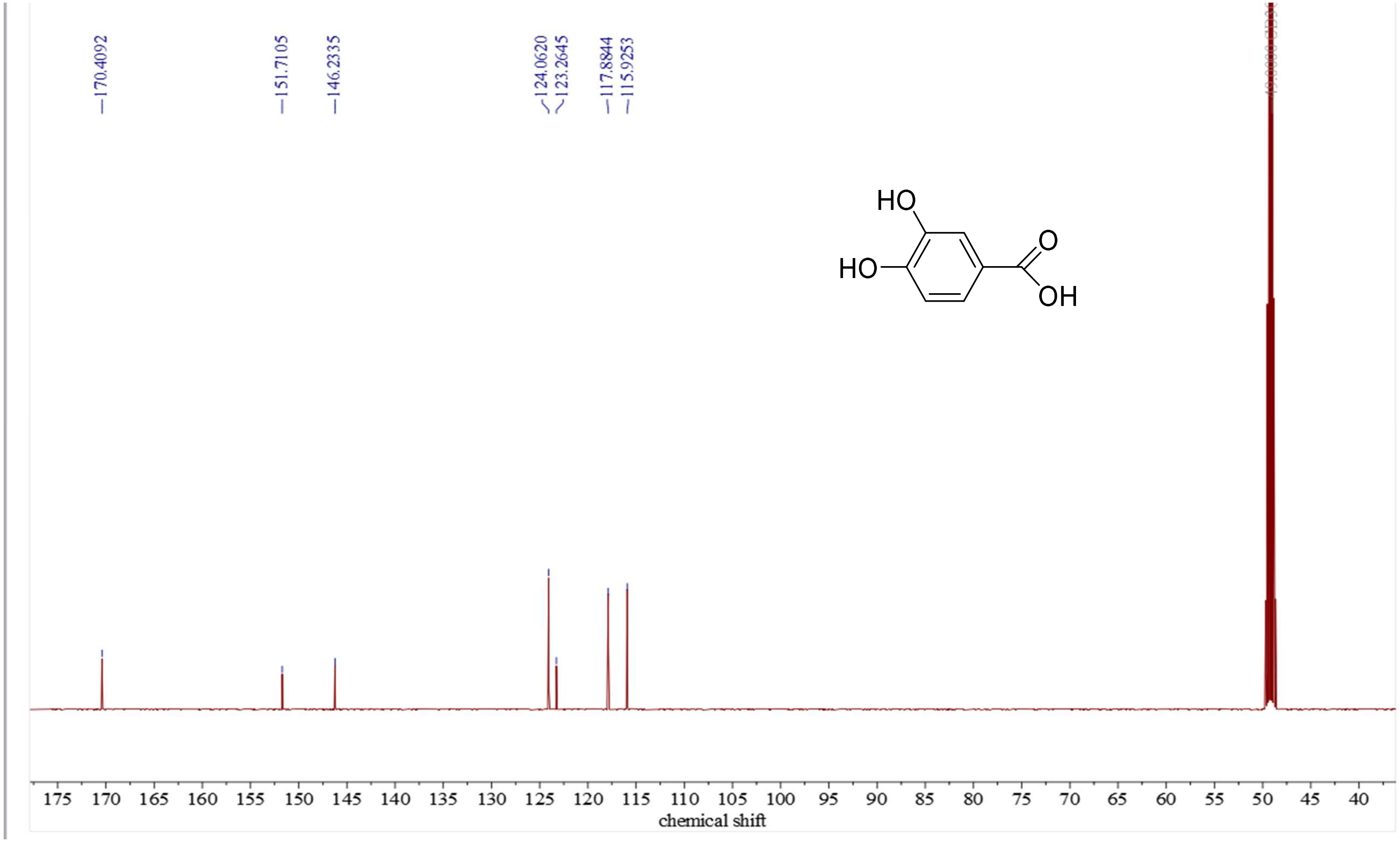

Figure S21. ^{1}H NMR spectrum of compound 11 in methanol-d_4.

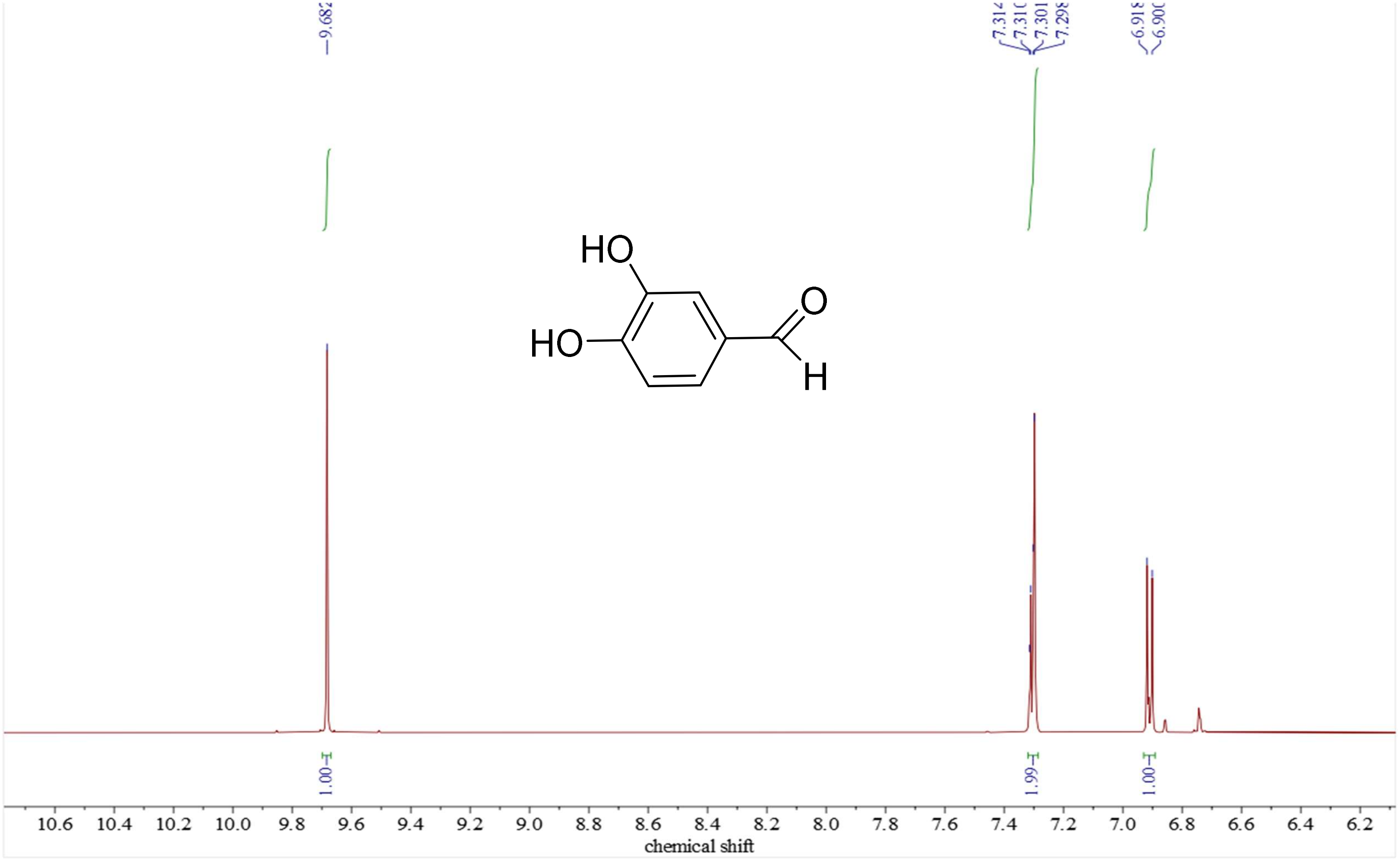

Figure S22. ^{13}C NMR spectrum of compound 11 in methanol-d_4.

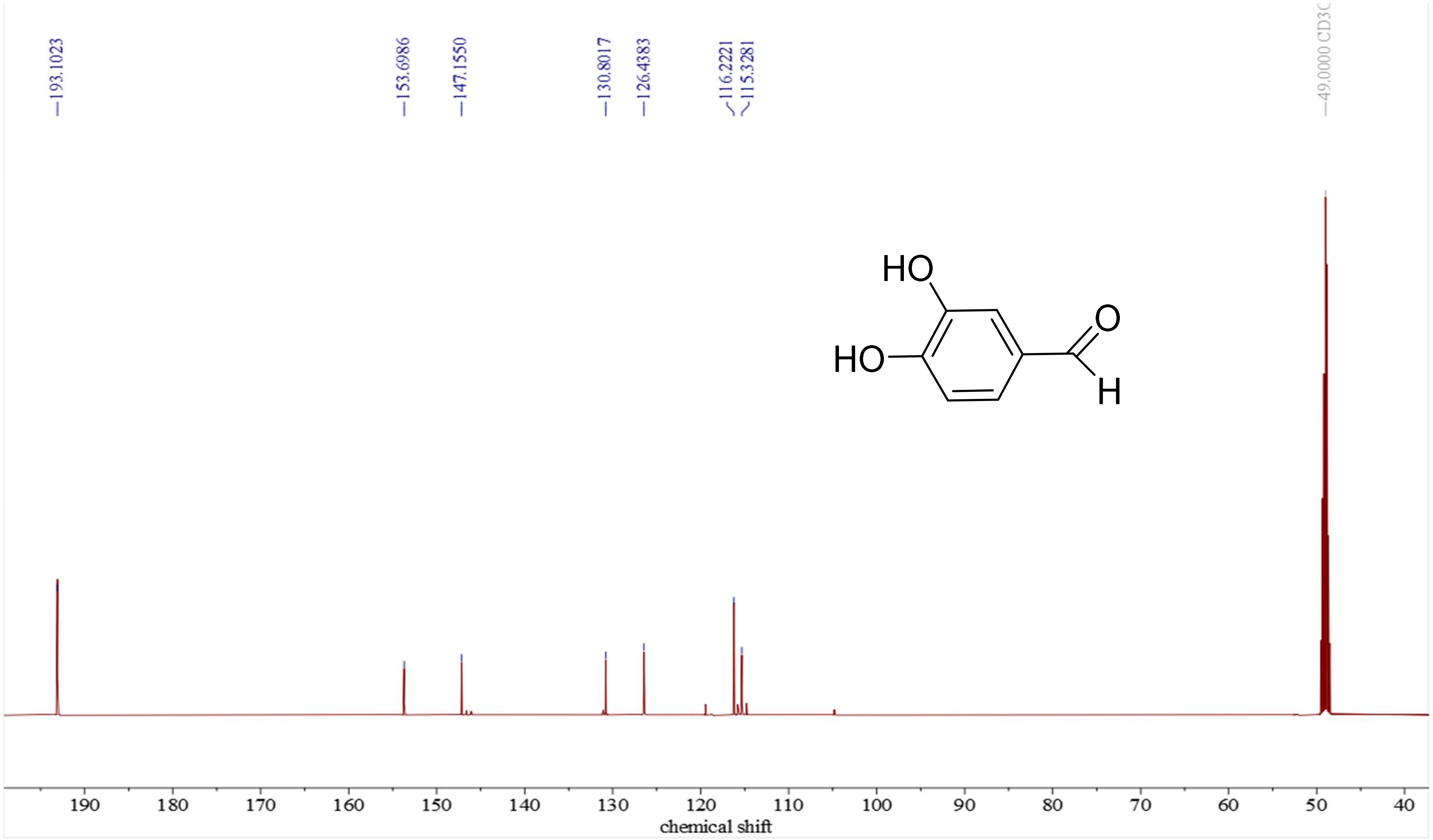

Figure S23. ^{1}**H NMR spectrum of compound 12 in methanol-*d*$_4$.**

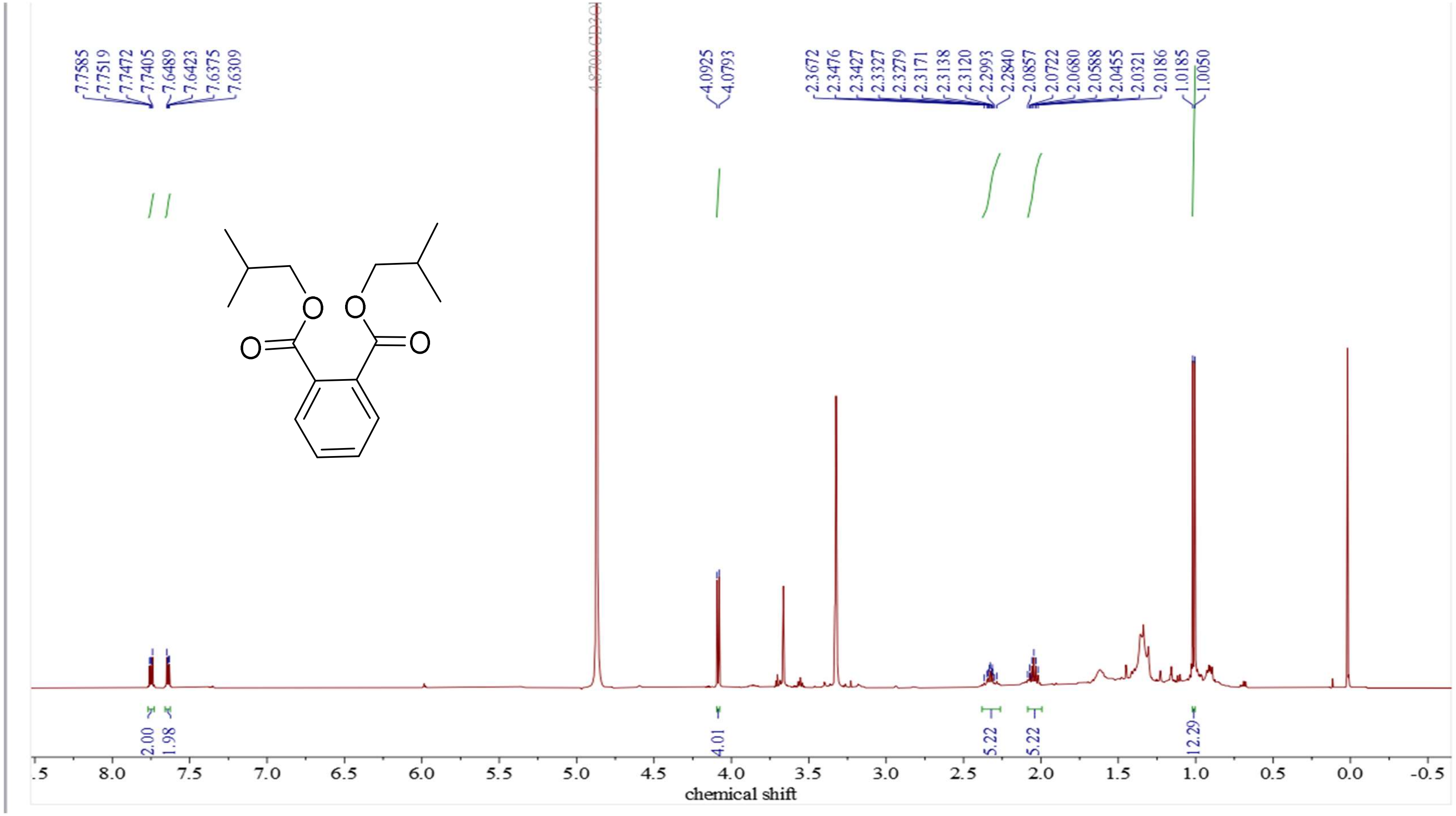

Figure S24. ^{13}C NMR spectrum of compound 12 in methanol-d_4.

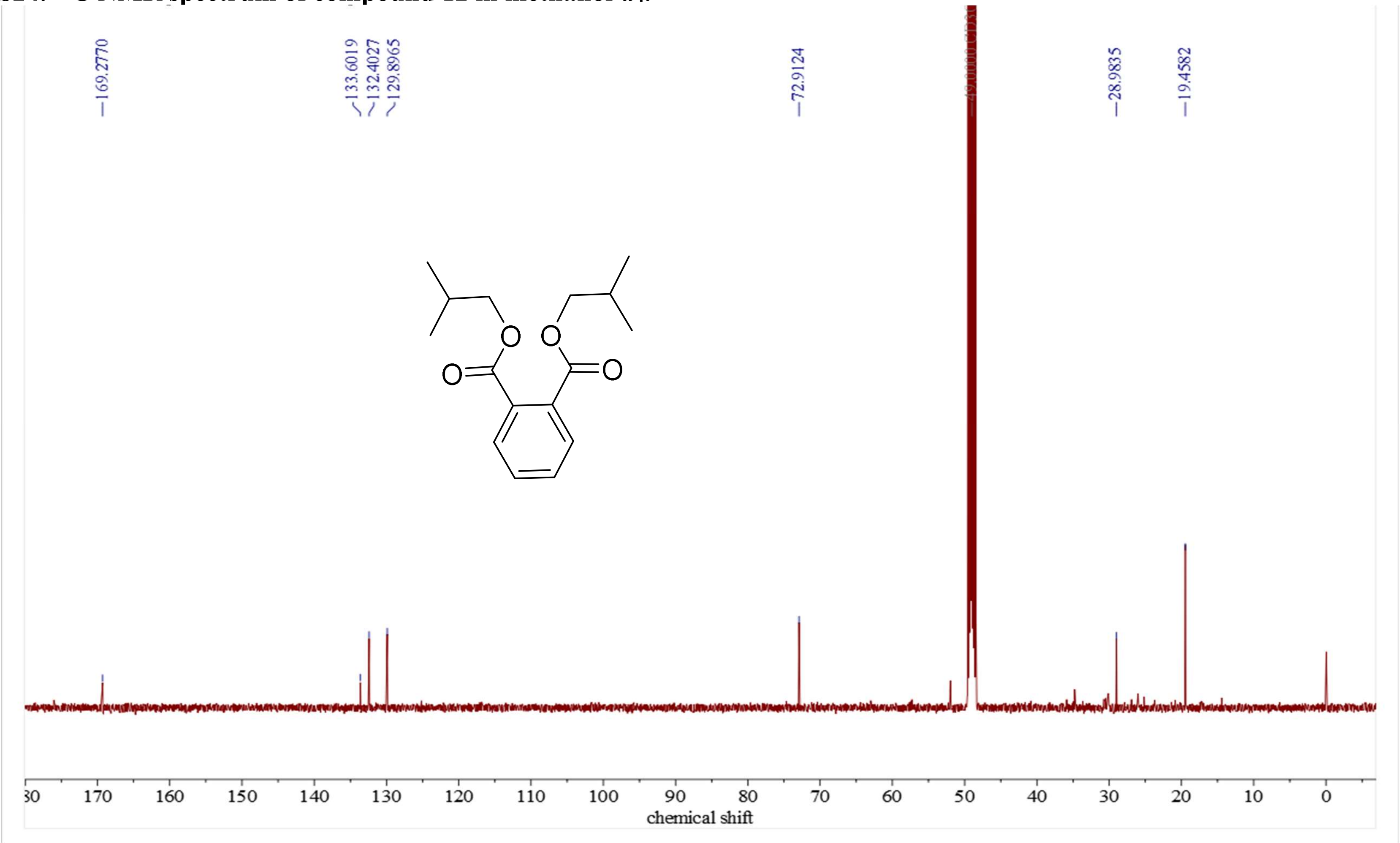

Figure S25. ^{1}H NMR spectrum of compound 13 in methanol-d_4.

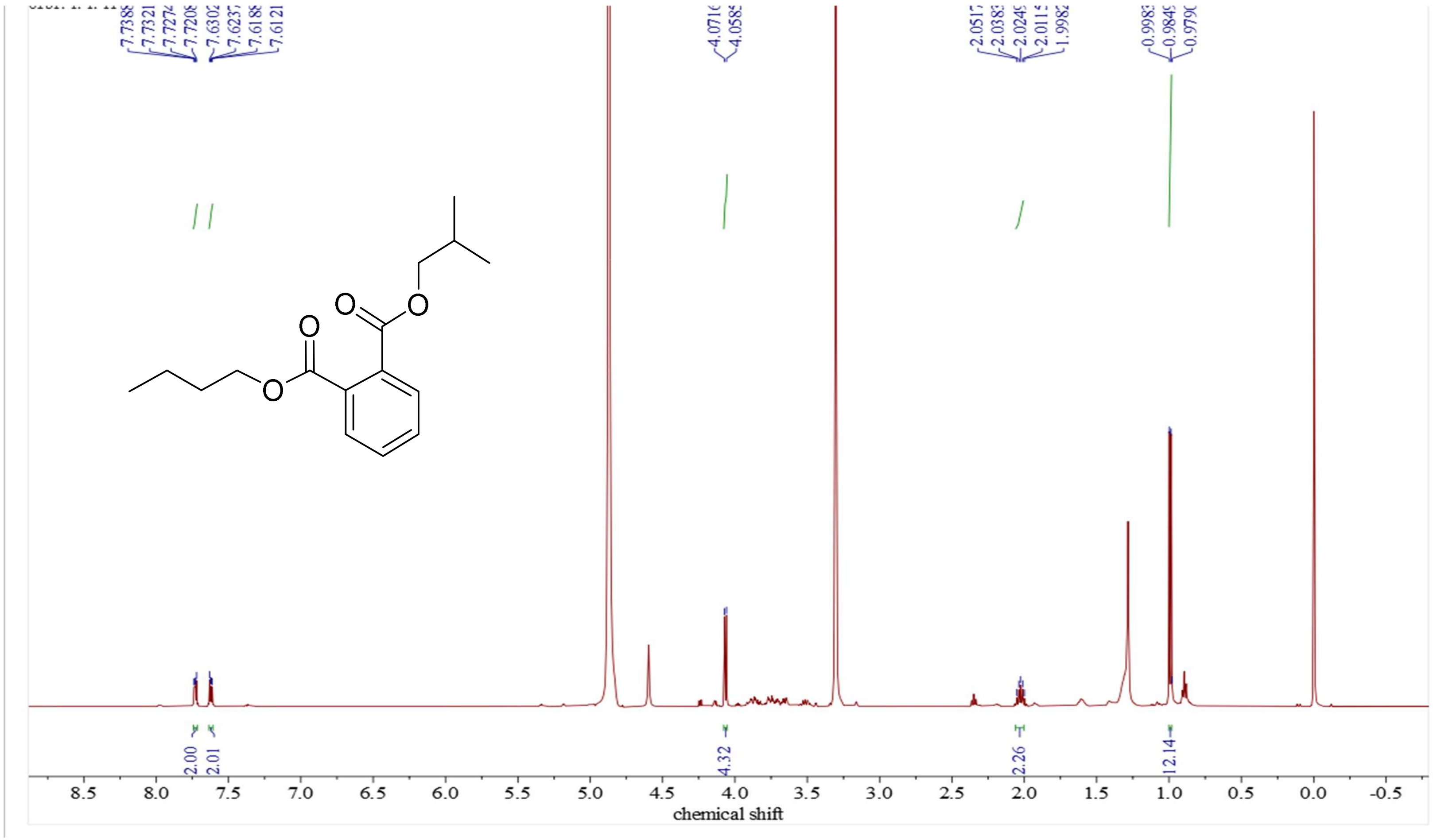

Figure S26. ^{13}C NMR spectrum of compound 13 in methanol-d_4.

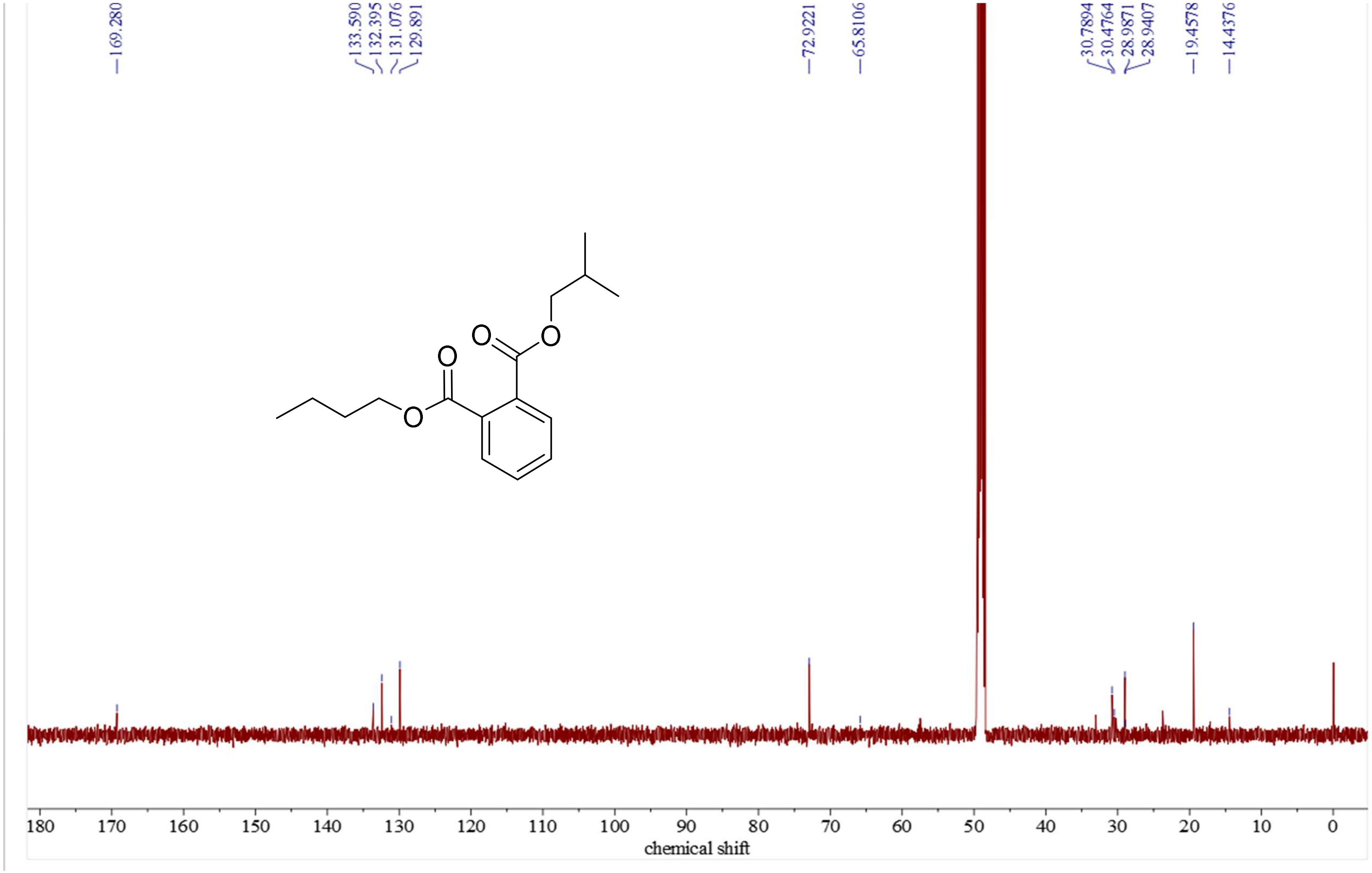

Figure S27. ^{1}H NMR spectrum of compound 14 in methanol-d_4.

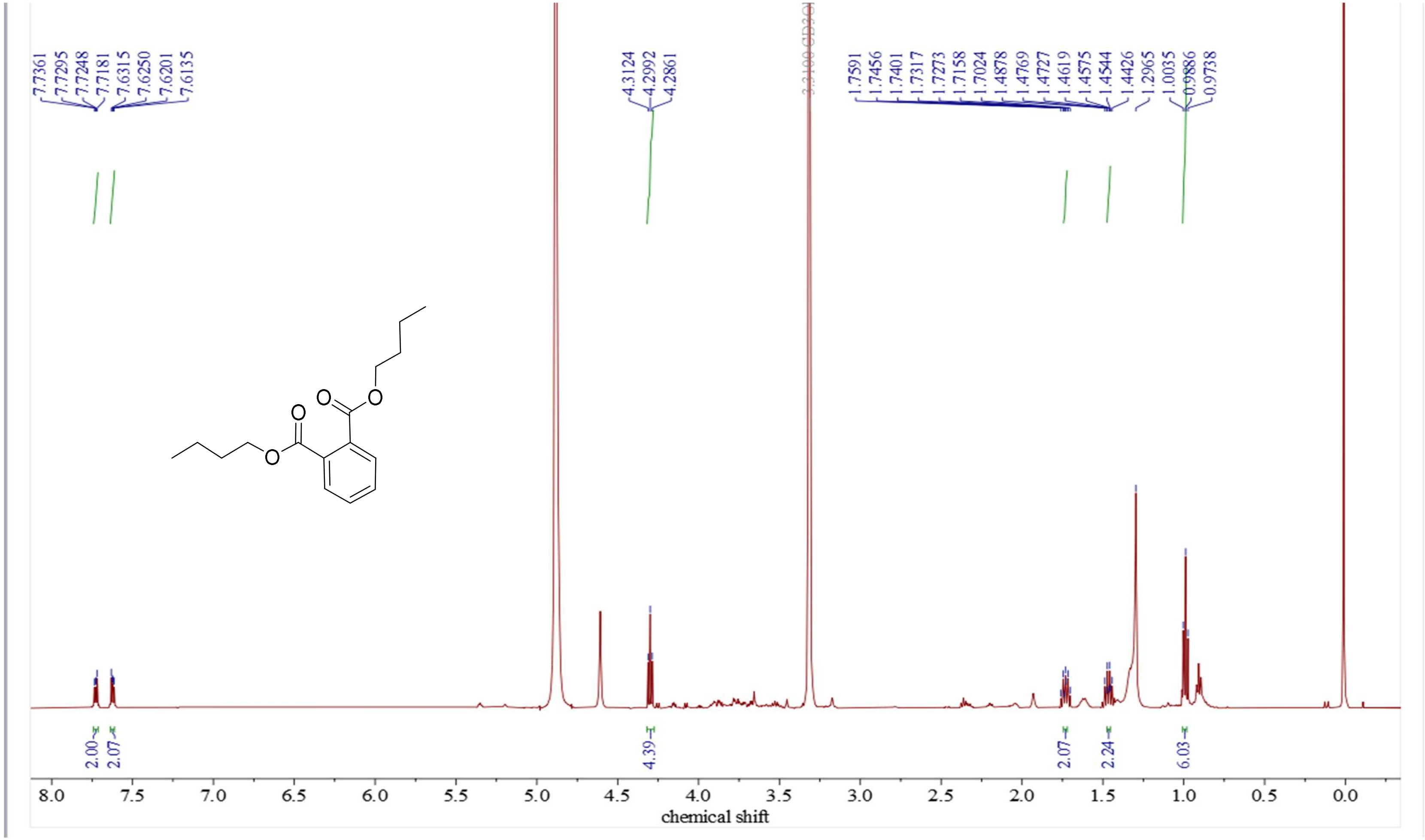

Figure S28. ^{13}C NMR spectrum of compound 14 in methanol-d_4.

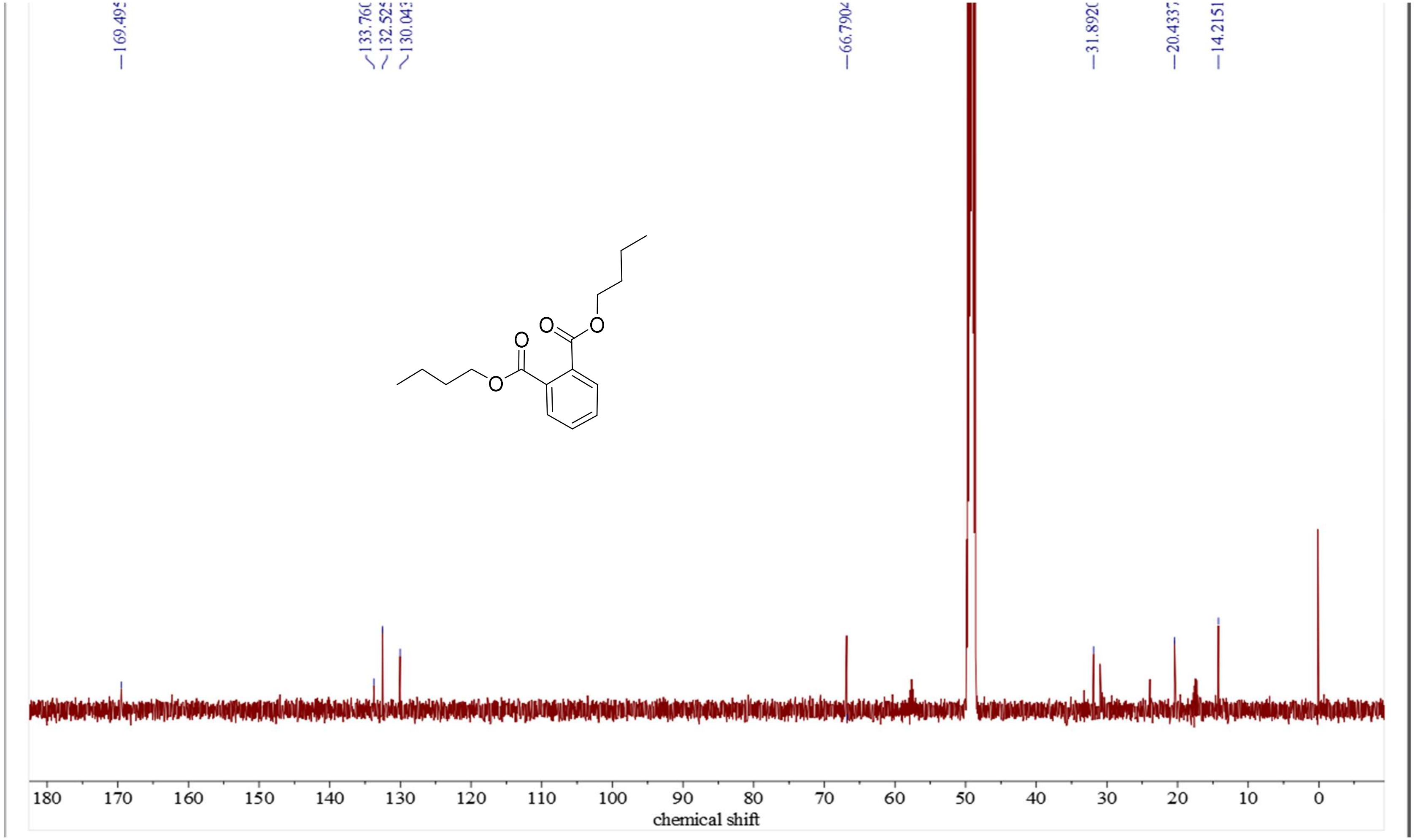

Figure S29. ^{1}H NMR spectrum of compound 15 in methanol-d_4.

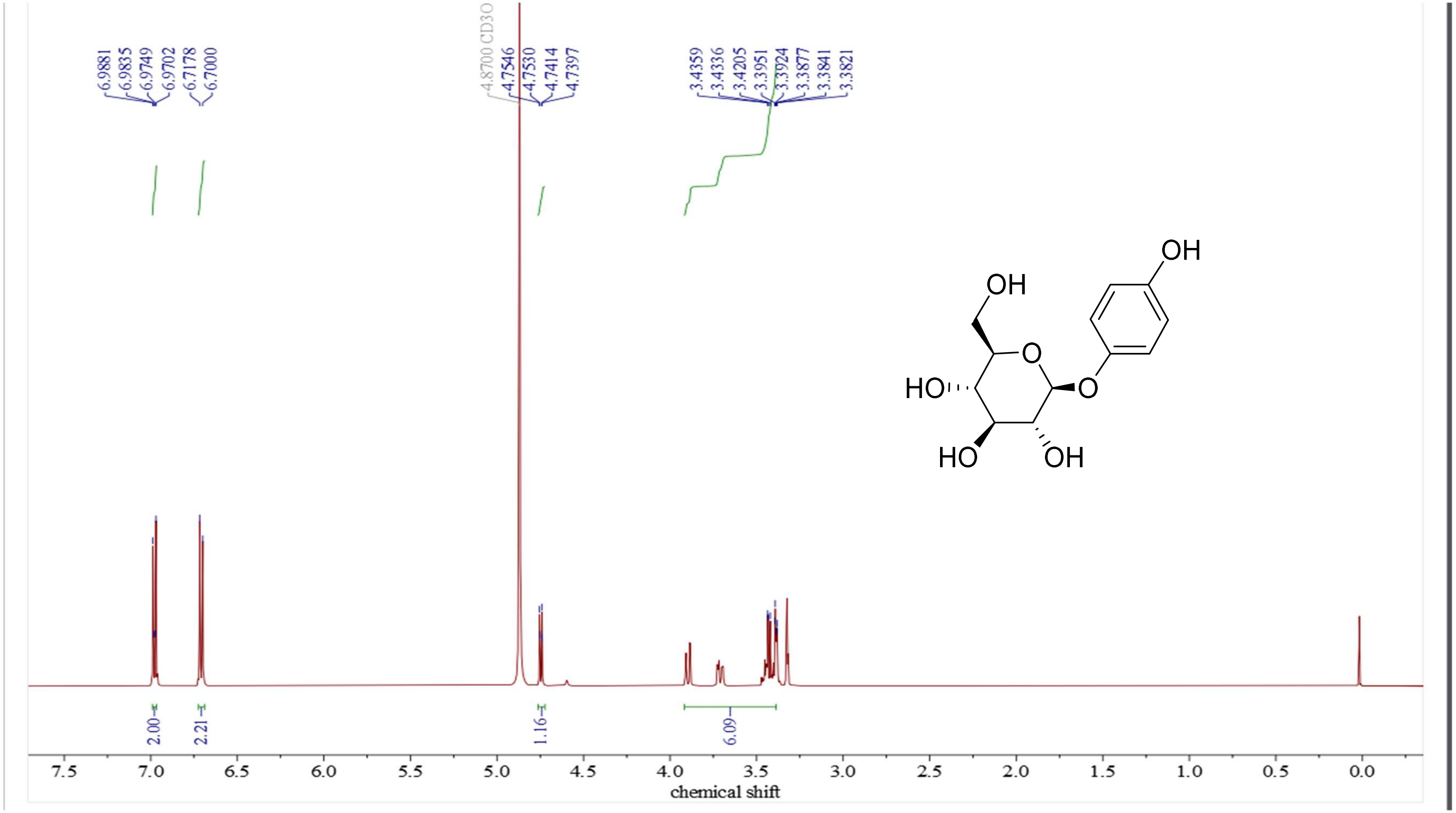

Figure S30. ^{13}C **NMR spectrum of compound 15 in methanol-*d*$_4$.**

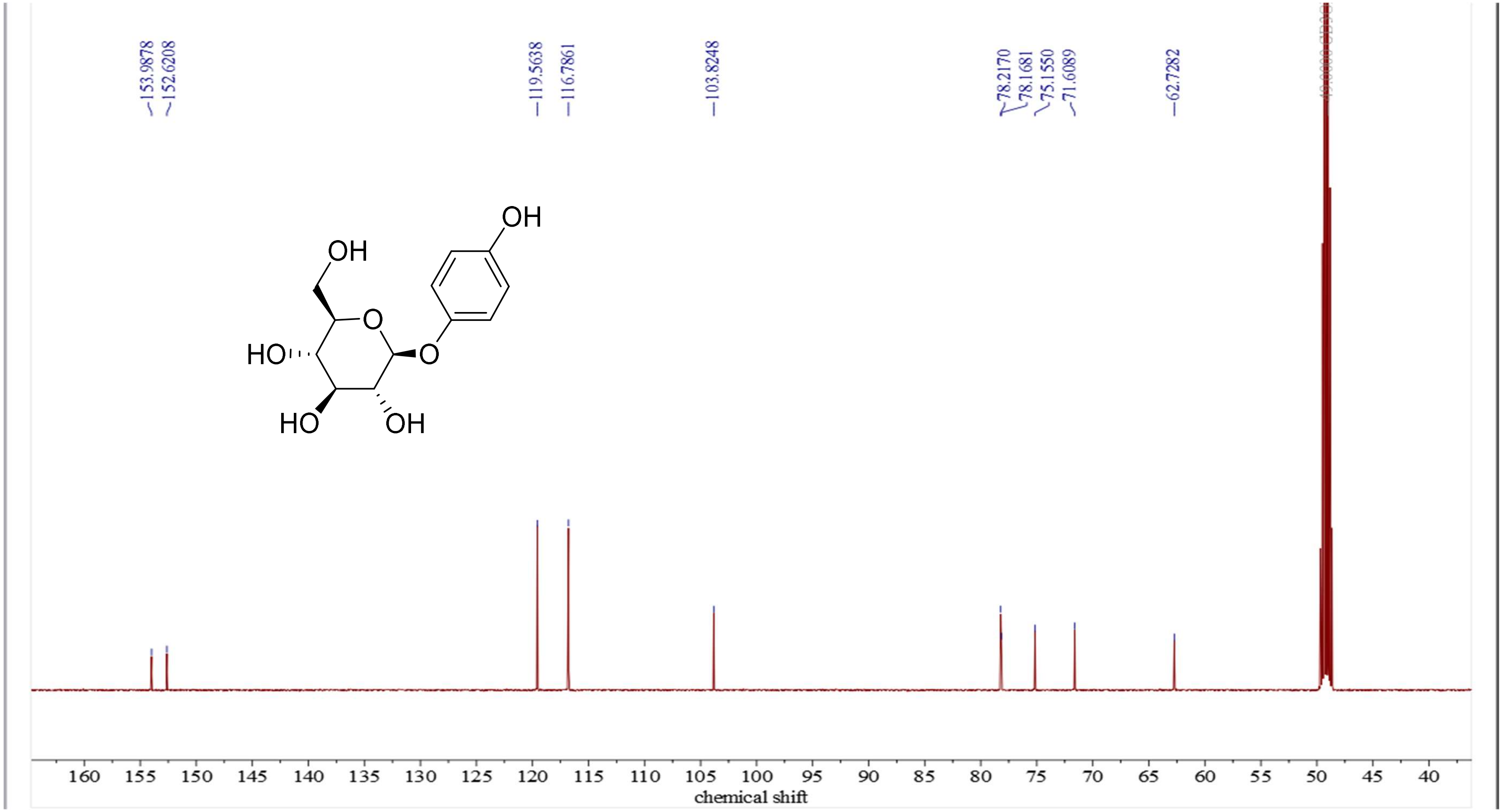

Figure S31. **^{1}H NMR spectrum of compound 16 in methanol-*d*4.**

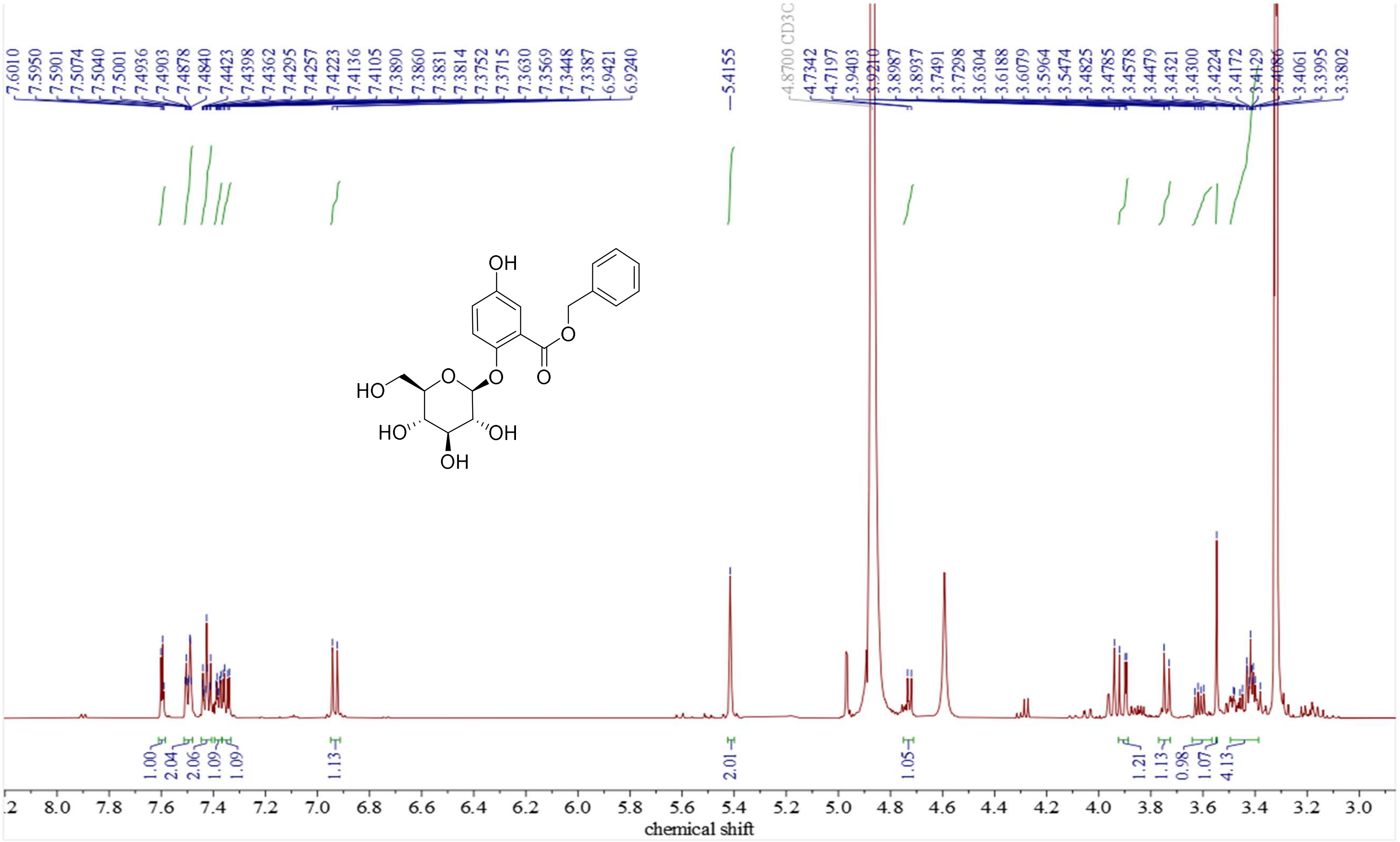

Figure S32. ^{13}C NMR spectrum of compound 16 in methanol-d_4.

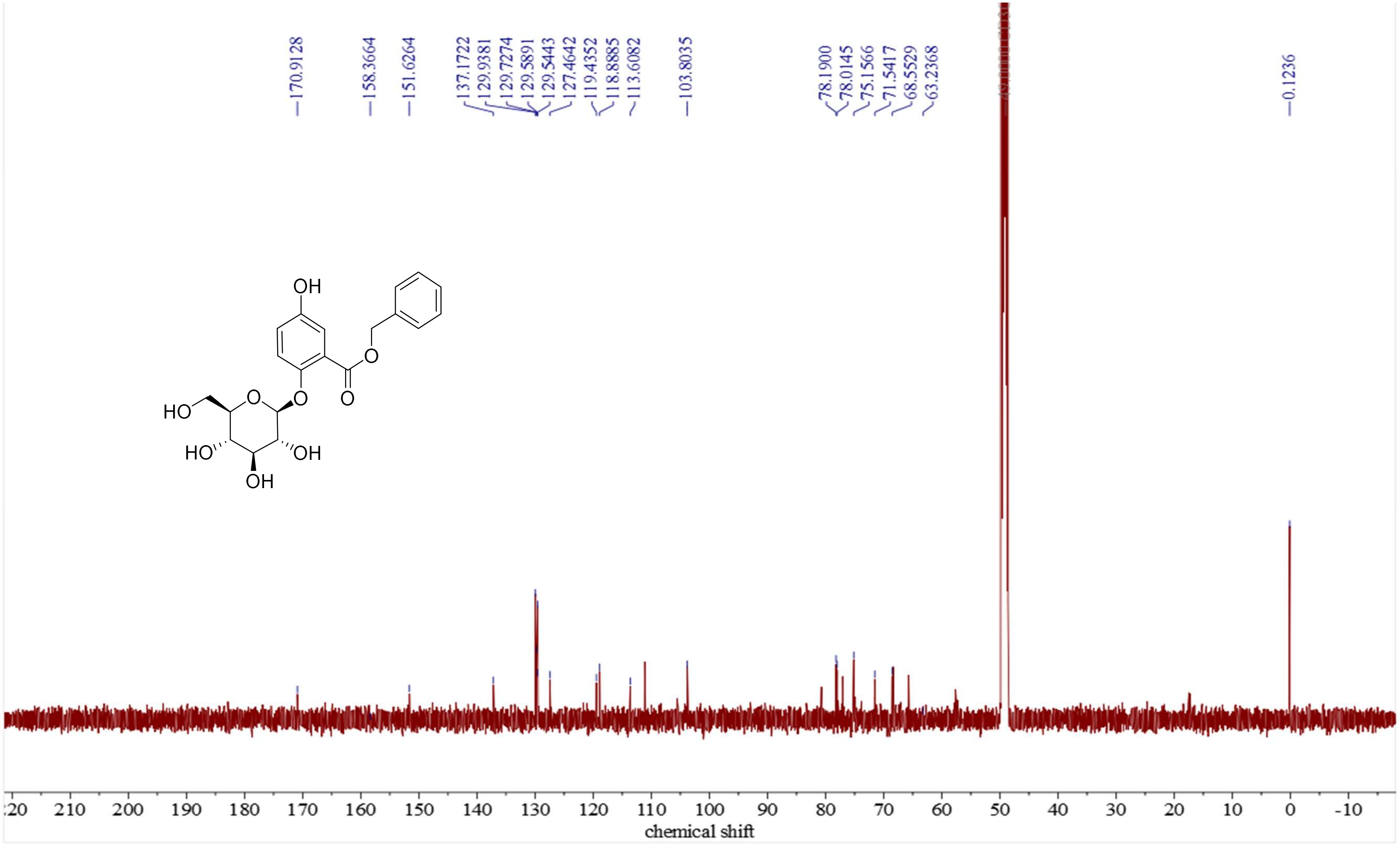

Figure S33. ^{1}H NMR spectrum of compound 17 in methanol-*d*$_4$.

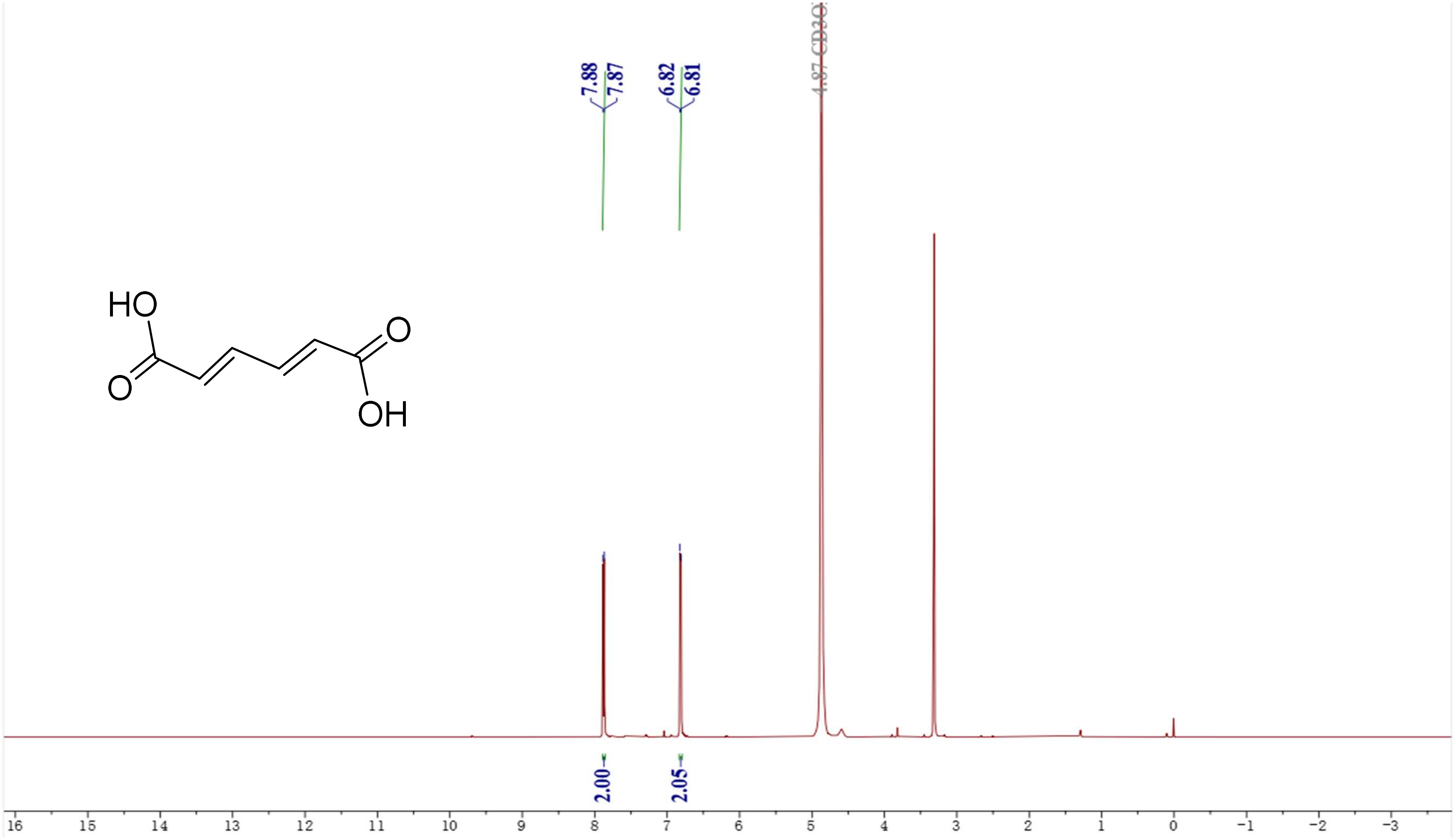

Figure S34. ^{13}**C NMR spectrum of compound 17 in methanol-*d*₄.**

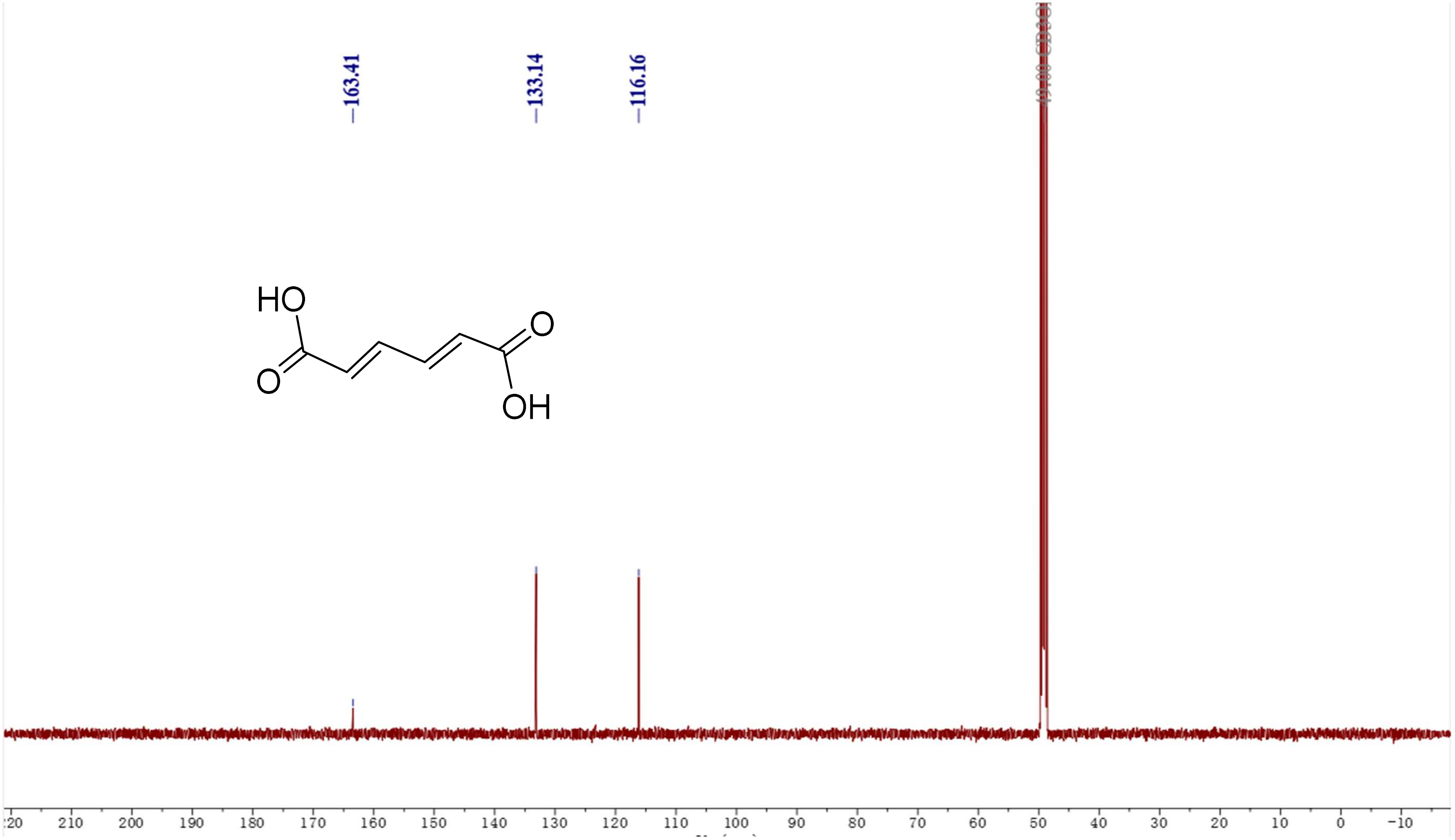

Figure S35. ^{1}H NMR spectrum of compound 18 in methanol-*d*$_4$.

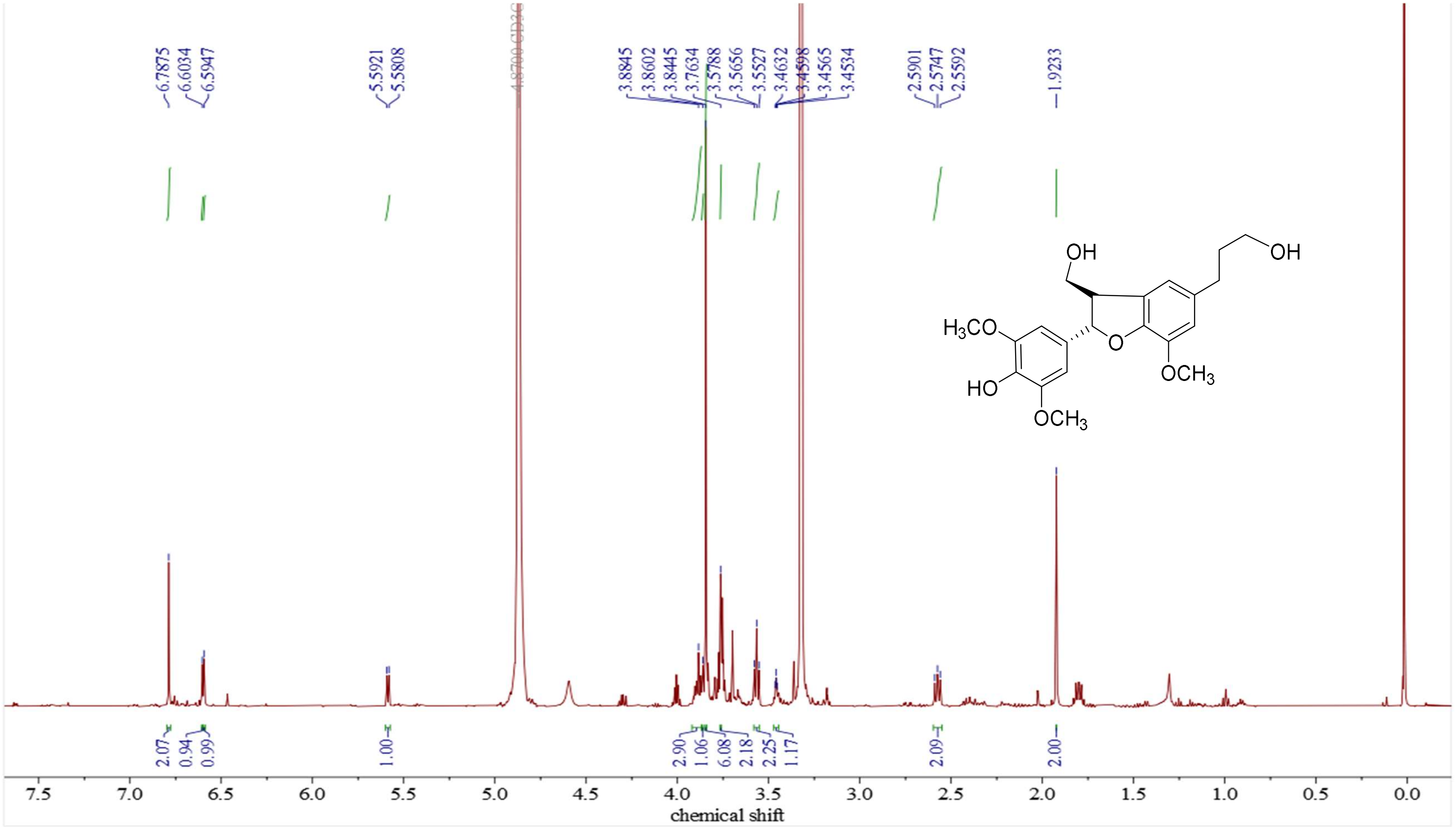

Figure S36. ^{13}C NMR spectrum of compound 18 in methanol-d_4.

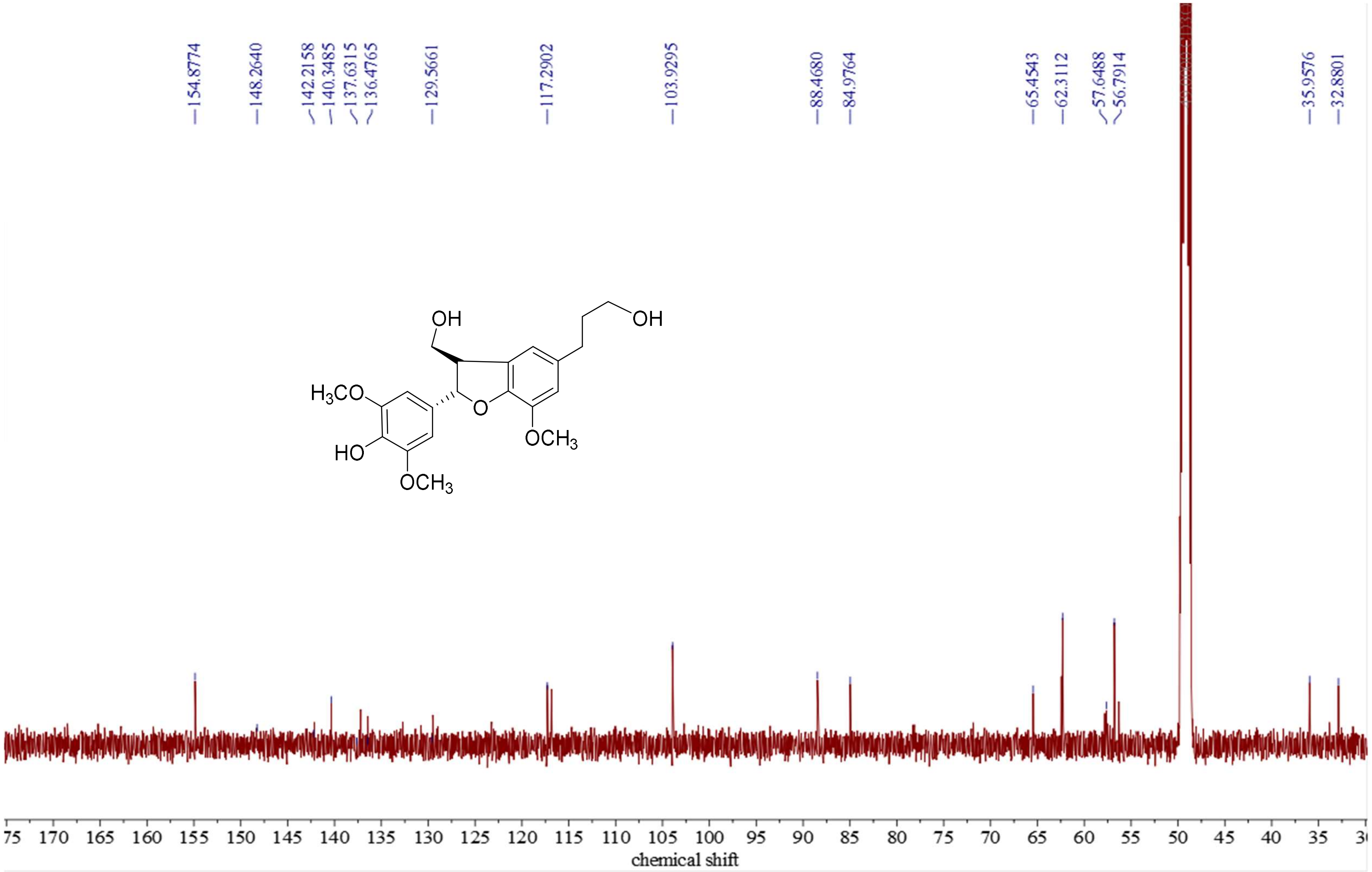

Supplement: Supplementary file 1 [file foods-13-03435-s001.zip › foods-3209463-supplementary.pdf]
